# Differential gene expression patterns between the head and thorax of *Gynaephora aureata* are associated with high-altitude adaptation

**DOI:** 10.3389/fgene.2023.1137618

**Published:** 2023-04-18

**Authors:** Jia-Rui Zhao, Shi-Yun Hu, Li-Jun Zhang, Li Zhang, Xing-Zhuo Yang, Ming-Long Yuan

**Affiliations:** ^1^ State Key Laboratory of Herbage Improvement and Grassland Agro-ecosystems, Lanzhou University, Lanzhou, China; ^2^ Key Laboratory of Grassland Livestock Industry Innovation, Ministry of Agriculture and Rural Affairs, Lanzhou, China; ^3^ Engineering Research Center of Grassland Industry, Ministry of Education, Lanzhou, China; ^4^ College of Pastoral Agriculture Science and Technology, Lanzhou University, Lanzhou, China

**Keywords:** grassland caterpillars, Lymantriinae, RNA-Seq, Qinghai-Tibetan Plateau, high-altitude adaptation, pigment-associated genes, olfactory-related genes

## Abstract

Grassland caterpillars (Lepidoptera: Erebidae: *Gynaephora*) are important pests in alpine meadows of the Qinghai-Tibetan Plateau (QTP). These pests have morphological, behavioral, and genetic adaptations for survival in high-altitude environments. However, mechanisms underlying high-altitude adaptation in QTP *Gynaephora* species remain largely unknown. Here, we performed a comparative analysis of the head and thorax transcriptomes of *G. aureata* to explore the genetic basis of high-altitude adaptation. We detected 8,736 significantly differentially expressed genes (sDEGs) between the head and thorax, including genes related to carbohydrate metabolism, lipid metabolism, epidermal proteins, and detoxification. These sDEGs were significantly enriched in 312 Gene Ontology terms and 16 KEGG pathways. We identified 73 pigment-associated genes, including 8 rhodopsin-associated genes, 19 ommochrome-associated genes, 1 pteridine-associated gene, 37 melanin-associated genes, and 12 heme-associated genes. These pigment-associated genes were related to the formation of the red head and black thorax of *G. aureata*. A key gene, *yellow-h,* in the melanin pathway was significantly upregulated in the thorax, suggesting that it is related to the formation of the black body and contributed to the adaptation of *G. aureata* to low temperatures and high ultraviolet radiation in the QTP. Another key gene, *cardinal*, in the ommochrome pathway was significantly upregulated in the head and may be related to red warning color formation. We also identified 107 olfactory-related genes in *G. aureata*, including genes encoding 29 odorant-binding proteins, 16 chemosensory proteins, 22 odorant receptor proteins, 14 ionotropic receptors, 12 gustatory receptors, 12 odorant degrading enzymes, and 2 sensory neuron membrane proteins. Diversification of olfactory-related genes may be associated with the feeding habits of *G. aureata*, including larvae dispersal and searching for plant resources available in the QTP. These results provide new insights into high-altitude adaptation of *Gynaephora* in the QTP and may contribute to the development of new control strategies for these pests.

## Introduction

The genus *Gynaephora* (Lepidoptera: Erebidae: Lymantriinae) includes 15 species worldwide, 8 of which are endemic to the Qinghai-Tibetan Plateau (QTP) (Yan, 2006; Zhang and Yuan, 2013). *Gynaephora* species and the most destructive insect pests in the alpine meadows of the QTP and have caused serious feed shortages and grassland degradation (Yan, 2006; Zhang and Yuan, 2013). The QTP environment is characterized by hypoxia, low temperatures, and high ultraviolet (UV) radiation (Yu et al., 2016). The spread of QTP *Gynaephora* species in the harsh environment of the QTP can be explained by various morphological and behavioral adaptations (Yan, 2006; Zhang and Yuan, 2013; Barrio et al., 2014). In terms of morphology, black body hair can resist high UV radiation and a red head can protect against natural enemies in larvae of QTP *Gynaephora* species (Yan, 2006; Zhang and Yuan, 2013). The feeding habits of *Gynaephora groenlandica* and *Gynaephora rossii* in the Arctic differ from those of QTP *Gynaephora* species (Barrio et al., 2014; Cao, 2022). Our recent research on gut bacteria, transcriptome profiles, and mitochondrial genomes has contributed to our understanding of the high-altitude adaptation of QTP *Gynaephora* species (Zhang L. et al., 2017; Zhang Q. L. et al., 2017; Yuan et al., 2018; Cao et al., 2021). However, the molecular mechanisms underlying high-altitude adaptation in QTP *Gynaephora* species are still largely unknown.

QTP *Gynaephora* species are also known as red-headed black caterpillars. Different from other grassland caterpillars, the heads of QTP *Gynaephora* species are bright red, and the thorax and abdomen are covered with black body hair; these morphological features are associated with high-altitude adaptation. The skin and hair color of humans (such as Tibetans on the QTP), mammals (e.g., *Psammodromus algirus* and *Sus scrofa domesticus*), amphibians, and reptiles in high-altitude areas are significantly darker than the skin of those in low-altitude areas (Wang et al., 2000; Brenner and Hearing, 2008; Jablonski and Chaplin, 2010; Li et al., 2013; Reguera et al., 2014; Visscher, 2017; Gao et al., 2019; Zhang et al., 2021). Organisms at high-altitude areas show a deeper body color to resist high UV radiation, which reduces the damage to cells and DNA structure caused by the excessive production of reactive oxygen species (Rinnerthaler et al., 2015). In addition to protecting against UV radiation damage, a deeper body color also plays an important role in regulating the body temperature (DeJong et al., 1996; Carothers et al., 1998; Geen and Johnston, 2014; Gao et al., 2019). Black areas in the abdomens of *Phrynocephalus* in the QTP are significantly larger than those of individuals in low-altitude areas, and these areas are conducive to raising the body temperature (Jin and Liao, 2015; Gao et al., 2019). Dense black body hair on the thorax and abdomen of the larvae of QTP *Gynaephora* species may be an adaptation to the QTP environment (high UV levels and low temperatures). In many insects, bright colors, as warning colors, appear together with black, which has important ecological functions (Sun M. X. et al., 2020). The red heads of QTP *Gynaephora* species may be an adaptation to resist natural enemies. The formation of insect body color is closely related to pigment-related genes in the rhodopsin, ommochrome, pteridine, melanin, and heme biosynthesis pathways (Sugumaran et al., 1990; Ferreira, 1995; Ziegler et al., 2000; Tripoli et al., 2005; Di Pietro et al., 2006; Harris et al., 2011; Croucher et al., 2013; Sun M. X. et al., 2020). However, pigment pathway-associated genes and the molecular mechanism underlying the formation of body color in QTP *Gynaephora* species are still unknown.

QTP *Gynaephora* species are typical polyphagous pests. *G. aureata*, *Gynaephora menyuanensis,* and *Gynaephora alpherakii* can feed on 77 species of plants in 18 families, mainly herbaceous plants, such as Cyperaceae and Gramineae (Cao, 2022). Compared with *G. groenlandica* and *G. rossii*, which mainly feed on Arctic willow (*Salix arctica*) and avoid eat grasses and sedges (MacLean and Jensen, 1985; Barrio et al., 2014), the feeding habits of QTP *Gynaephora* species differ substantially. The change in feeding habits of QTP *Gynaephora* species is an adaptation to the abundant plant resources available in the QTP. Host finding relies on the olfactory system in insects to recognize environmental signals, such as volatiles from plants (Gols et al., 2012; Suh et al., 2014; Ballesteros et al., 2019). Insects can adapt to changes in host plants by adjusting their olfactory system (Linz et al., 2013; Claudianos et al., 2014; Ballesteros et al., 2019). Therefore, the change in the feeding habits of QTP *Gynaephora* is an olfactory system adaptation to the host plants. Insects have different types of olfactory proteins, with different roles in host preferences (Ballesteros et al., 2019). Odorant-binding proteins (OBPs) and chemosensory proteins (CSPs) in the sensillar lymph of insect antennae can bind and transport lipophilic odorant molecules (Wanner et al., 2005; Pelosi et al., 2006; Guo et al., 2011; Dippel et al., 2014; Pelosi et al., 2014; Pelosi et al., 2018; Liu et al., 2020). Ionotropic receptors (IRs) play a key role in sensing different odors, such as acids, salts, ammonia, and aldehyde (Rytz et al., 2013; Chen et al., 2015; Enjin et al., 2016; Knecht et al., 2016; Frank et al., 2017; Zhang et al., 2019). IRs and odorant receptor proteins (ORs) expressed on the olfactory receptor neuron membrane detect transported odorant molecules (Benton et al., 2009; Abuin et al., 2011). Gustatory receptors (GRs) mainly detect sugar, bitterness, and pheromones and have important functions in finding hosts (Clyne et al., 2000; Park and Kwon, 2011; Mang et al., 2016). Odorant degrading enzymes (ODEs) are involved in the degradation of odorant molecules (Leal, 2013). Sensory neuron membrane protein genes (SNMPs) play an important role in the identification and detection of pheromones (Clyne et al., 1999; Mang et al., 2016). However, genes in the olfactory system of QTP *Gynaephora* species have not been characterized.

In this study, the first transcriptomic analyses of *G. aureata* were conducted *via* high-throughput sequencing. Significantly differentially expressed genes (sDEGs) were identified and analyzed *via* Gene Ontology (GO) and Kyoto Encyclopedia of Genes and Genomes (KEGG) pathway analyses. To explore the genetic basis of high-altitude adaptation in QTP *Gynaephora* species, 73 pigment-pathway associated and 107 olfactory-related genes in *G. aureata* were identified. The study results improve our understanding of high-altitude adaptation in QTP *Gynaephora* species and may guide the development of pest control strategies.

## Materials and methods

### Sample collection and RNA extraction

Specimens of 4th instar larvae (red head and black thorax) of G. aureata were collected from Azi Town, Maqu County, Gansu Province, China (3,400 m above sea level, 101°52′E, 33°40′N). Larvae were dissected under a dissecting microscope to collect the head and thorax into separate tubes. Six samples, including three biological replicates for the head (MQAZT1, MQAZT2, and MQAZT3) and thorax (MQAZX1, MQAZX2, and MQAZX3), were immediately frozen in liquid nitrogen and stored at −80°C before RNA extraction.

Total RNA was extracted using the RNA Extraction Kit RNeasy Mini Kit (Qiagen, Beijing, China) according to the manufacturer’s instructions. Residual genomic DNA was digested with RNase-free DNase (Qiagen, Hilden, Germany). The integrity and concentration of RNA were measured using 1.5% agarose gel electrophoresis and the NanoDrop ND1000 spectrophotometer (Thermo Scientific, Waltham, MA, United States), respectively.

### RNA sequencing and cDNA library construction

cDNA library construction was performed using the NEBNext Ultra RNA Library Prep Kit for Illumina (New England Biolabs, Ipswich, MA, United States). Messenger RNAs (mRNAs) were enriched from total RNAs of *G. aureata* using oligo (dT) magnetic beads and then fragmented into short nucleotides using the fragmentation buffer solution. The cleaved mRNA was transcribed into first-strand cDNA using random hexamer primers and buffer solution. Second-strand cDNA was subsequently synthesized using buffer solution, deoxyribonucleotide triphosphate, DNA polymerase I, and Ribonuclease H. After end-repairing, dA-tailing, and adaptor ligation, the products were amplified by PCR and purified using the QIAquick PCR Purification Kit (Qiagen, Valencia, CA, United States) to create the final sequencing library. Finally, the Illumina HiSeq2,500 platform (Illumina, San Diego, CA, United States) was used for sequencing (PE150). High-throughput sequencing of head and thorax RNA of *G. aureata* was completed by Annoroad Gene Technology Co., Ltd., Beijing, China.

### *De novo* assembly and functional annotation

To ensure analysis quality, the adapters, reads with >15% low-quality bases (*Q* value ≤ 19), and reads with unknown nucleotide (N) ratios greater than 5% were removed before *de novo* assembly. *De novo* transcriptome assembly was performed using Trinity (version 20140717) with default parameters (Grabherr et al., 2011), and unigene libraries of the high-quality head and thorax transcriptomes of *G. aureata* were obtained.

After assembly and evaluation, TransDecoder (version 20140717) was used to identify open reading frames (ORF). Trinotate (version 20140717) was used to predict protein signals and annotate unigenes in various databases, including the non-redundant (Nr) database at the National Center for Biotechnology Information (NCBI), the nucleotide (Nt) database at NCBI, protein family (Pfam) database, evolutionary genealogy of genes: Non-supervised Orthologous Groups (eggNOG) database, Gene Ontology (GO) database, Eukaryotic Orthologous Groups (KOG) database, and Kyoto Encyclopedia of Genes and Genomes (KEGG) database, with a cut-off *E*-value of 10^–5^ (Anderson and Brass, 1998).

### Analysis of differentially expressed genes

Reads per kilobase million mapped reads (RPMK) were used to quantify gene expression levels (Mortazavi et al., 2008). DESeq2 (version 1.4.5) (Nunes et al., 2016) was adopted to analyze differentially expressed genes (DEGs). A gene with |Log_2_ Fold Change| ≥ 1 and FDR (corrected *p*-value) < 0.05 was considered DEGs and a gene with |Log_2_ Fold Change| ≥ 1 and FDR < 0.01 was considered an sDEG. The sDEGs were mapped to GO annotations using Blast2GO and to KEGG pathways using KOBAS. GO terms and pathways with *Q* ≤ 0.05 were regarded as significantly enriched in this study.

To verify the expression of head and thorax transcriptomes of *G. aureata*, quantitative real-time PCR (qRT-PCR) was performed for six DEGs. Primer Premier 5.0 was used to design qRT-PCR primers for DEGs (Supplementary Table S1). Elongation factor 1 alpha (*EF-1α*) was used as an internal reference gene. cDNA was synthesized from total RNA using the PrimeScript RT Reagent Kit with gDNA Eraser (Takara Bio Inc., Kusatsu, Japan) according to the recommended protocol. All qRT-PCR analyses were performed in an ABI7500 real-time PCR system (Applied Biosystems, United States) using TB Green^®^ *Premix Ex Taq* ^*TM*^ Ⅱ (Takara Bio Inc.), according to the recommended protocol. The PCR system and reaction conditions were conducted as described previously (Zhang L. et al., 2017). Relative gene expression levels were calculated using the 2^−ΔΔCT^ method.

### Identification and analysis of pigment pathway and olfactory-related genes

To identify candidate genes associated with the adaptation of *Gynaephora,* genes in major pigment pathways and olfactory-related genes were comprehensively screened. For pigment-pathway associated genes, referring to a previously described method, pigment-pathway associated proteins (n = 109) of *Drosophila melanogaster* were selected from the AmiGO database under the category “Pigment Metabolic Process” (GO: 0042440) (Croucher et al., 2013). The sequences (n = 109) of pigment-pathway associated proteins of *D. melanogaster* were downloaded from the NCBI database and searched by the BLASTP algorithm (*E* value < 10–5) against BLAST databases constructed from the head and thorax transcriptomes of *G. aureata* (Supplementary Table S2). Then, “significant” BLAST hits were extracted and compared by BLASTP (*E* value < 10–5) against the Nr database to obtain the pigment-pathway associated genes of *G. aureata* (Supplementary Table S2).

“OBP and odorant binding protein,” “CSP and chemosensory protein,” “OR and odorant receptor protein,” “IR and ionotropic receptor,” “GR and gustatory receptor,” “ODE and odorant degrading enzyme,” “CXE and carboxylesterase,” and “SNMP and sensory neuron membrane protein gene” were used as keywords to screen putative olfactory-related genes in the annotated head and thorax transcriptomes. All putative OBPs, CSPs, ORs, IRs, GRs, ODEs, and SNMPs were manually checked using the BLASTX program of NCBI with a cut-off *E* value of 10^–5^.

The putative N-terminal signal peptides and transmembrane domains (TMDs) of olfactory-related genes were predicted using SignalP 4.1 and TMHMM 2.0, respectively. Amino acid sequences were aligned using ClustalX (Thompson et al., 1994). MEME was used for motif pattern analyses of OBPs and CSPs to confirm the accuracy of annotations (Supplementary Table S3).

## Results

### Transcriptome sequencing, assembly, and functional annotation

We obtained more than 4.9 Gb of raw reads from each sample in the head and thorax transcriptomes of *G. aureata*. The transcriptomes of three head samples of *G. aureata* yielded 45,053,582 (89.67%, MQAZT1), 47,478,452 (90.21%, MQAZT2), and 44,486,760 (96.29%, MQAZT3) clean reads. The transcriptomes of three thorax samples of *G. aureata* yielded 45,606,984 (89.71%, MQAZX1), 44,377,778 (89.7%, MQAZX2), and 45,345,264 (90.04%, MQAZX3) clean reads. The Q30 value in each sample was above 96.20% (Supplementary Table S4). After clean reads for six samples of head and thorax were assembled, a total of 132,598 unigenes were generated (Supplementary Table S5), with a maximum length of 31,778 bp, minimum length of 201 bp, and average length of 618 bp (Supplementary Figure S1). The raw reads have been deposited in the NCBI Sequence Read Archive (SRA) database under accession numbers SRR22475456-SRR22475461 (MQAZT1-3 and MQAZX1-3).

A total of 40,592 unigenes (30.61%) of *G. aureata* were successfully annotated to seven databases (Supplementary Table S6). The numbers of reference gene sequences annotated in Nr, KOG, Nt, GO, KEGG, eggNOG, and Pfam were 31,872 (24.04%), 8,801 (6.64%), 8,077 (6.09%), 19,385 (14.62%), 2,907 (2.19%), 12,153 (9.17%), and 16,531 (12.47%), respectively (Supplementary Table S6). Among the annotated unigenes, 50.56% had best matches to lepidopteran sequences, primarily *Bombyx mori* (26.72%), *Plutella xylostella* (12.41%), and *Danaus plexippus* (11.43%) (Supplementary Figure S2). GO annotation was used to classify the unigenes into 67 functional groups in three main categories. Of 40,592 unigenes of *G. aureata*, 19,385 (14.62%) were annotated (Supplementary Figure S3). Moreover, among the 40,592 unigenes, 8,801 unigenes (6.64%) were classified into 24 KOG categories (Supplementary Figure S4).

### Differentially expressed genes

**FIGURE 1:**
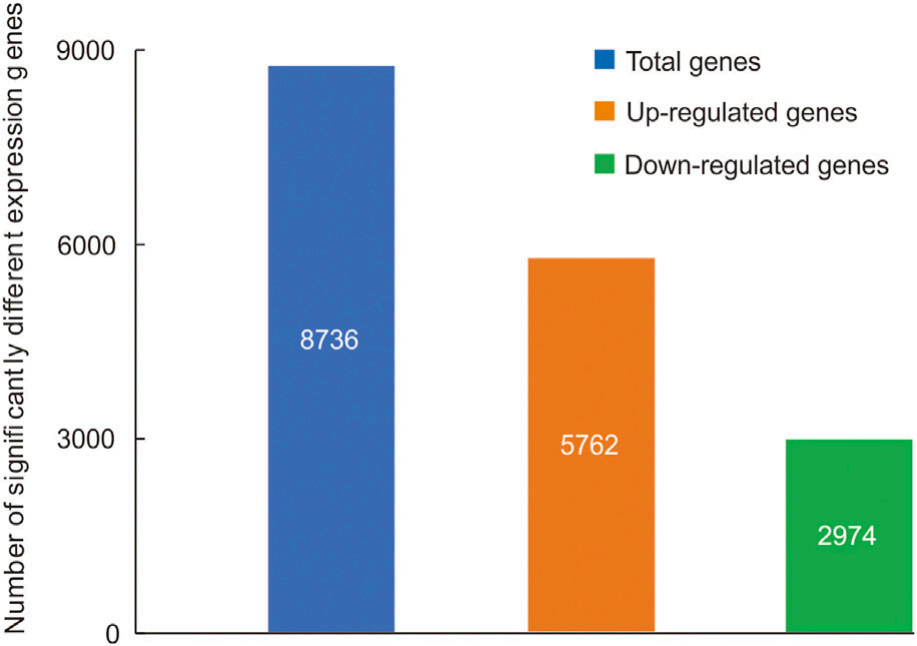
Significantly differentially expressed genes (sDEGs) between the head and thorax transcriptomes of *Gynaephora aureata*.

A total of 8,736 genes were significantly differential expressed between the head and thorax (Supplementary Table S7), 5,762 genes were upregulated and 2,974 genes were downregulated in the thorax compared with the head (Figure 1). Among sDEGs, 65 upregulated sDEGs and 14 downregulated were related to carbohydrate metabolism, 76 upregulated and 13 downregulated sDEGs were related to lipid metabolism, and 49 upregulated sDEGs and 5 downregulated sDEGs were related to detoxication. Among 66 genes encoding cuticular proteins, 14 were significantly upregulated and 52 were downregulated. We also detected three upregulated sDEGs related to the immune response, and two sDEGs (one upregulated and one downregulated) related to DNA repair (Supplementary Table S8). The results of high-throughput sequencing were verified by qRT-PCR for six DEGs (Supplementary Figure S5). GO functional analysis showed that 2,655 sDEGs (30.39% of 8,736 genes) were enriched for terms in the three GO categories: biological process (169 GO terms), cellular component (22 GO terms), and molecular function (121 GO terms) (Supplementary Table S9). In the cellular component category, the terms “regulation of peptidoglycan recognition protein signaling pathway” and “negative regulation of peptidoglycan recognition protein signaling pathway” were enriched (Supplementary Table S9; Supplementary Figure S6). “Myosin filament” was the most highly enriched in the molecular function domain (Supplementary Table S9; Supplementary Figure S6). In the biological process category, “l-threonine ammonia-lyase activity” and “amidase activity” were the enriched components (Supplementary Table S9; Supplementary Figure S6).

In total, 2,907 sDEGs (33.28% of 8,736 genes) were annotated to 335 KEGG pathways, including 16 significantly enriched pathways (Supplementary Figure S7). We found that seven pathways related to metabolic reactions were enriched, including two pathways related to carbohydrate metabolism (“Other glycan degradation” and “Galactose metabolism”), three pathways related to lipid metabolism (“Glycosphingolipid biosynthesis-globo series,” “Fatty acid elongation,” and “Fat digestion and absorption”), and two pathways related to amino acid metabolism (“Tryptophan metabolism” and “Glycine, serine and threonine metabolism”). sDEGs were also involved in two pathways (“Lysosome” and “Peroxisome”) related to antioxidant and detoxication (Supplementary Figure S7).

### Identification of pigment pathway-associated genes

**FIGURE 2:**
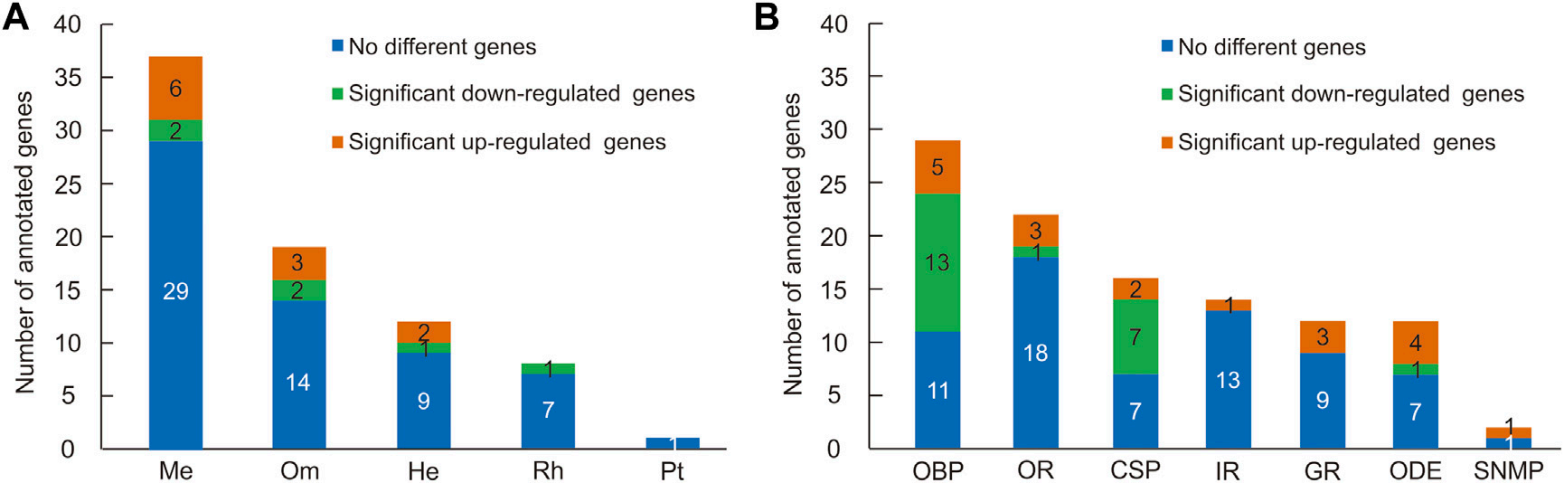
Numbers of pigment pathway and olfactory-related genes identified in the head and thorax transcriptomes of *Gynaephora aureata*. **(A)** Numbers of five kinds of pigment pathway genes. Pigment pathway genes are abbreviated as follows: melanin-associated genes (Me), ommochrome-associated genes (Om), heme-associated genes (He), rhodopsin-associated genes (Rh), and pteridine-associated genes (Pt). **(B)** Numbers of seven kinds of olfactory-related genes. Olfactory related genes are abbreviated as follows: odorant-binding protein (OBP), olfactory receptor (OR), chemosensory protein (CSP), ionotropic receptor (IR), gustatory receptor (GR), odorant degrading enzyme (ODE), and sensory neuron membrane protein (SNMP).

Pigment pathway-associated genes can be divided into five categories, namely, rhodopsin, ommochrome, pteridine, melanin, and heme. In this study, we searched the head and thorax transcriptomes of *G. aureata* for homologues corresponding to proteins obtained from the “Pigment Metabolic Process” GO term of *D. melanogaster* based on the AmiGO database. Of 109 *D. melanogaster* proteins, 99 proteins had putative homologues in *G. aureata* (Supplementary Table S2) and 73 proteins were identified as possible homologues by reciprocal best hits (RBH) (Supplementary Table S2; Figure 2).

**FIGURE 3:**
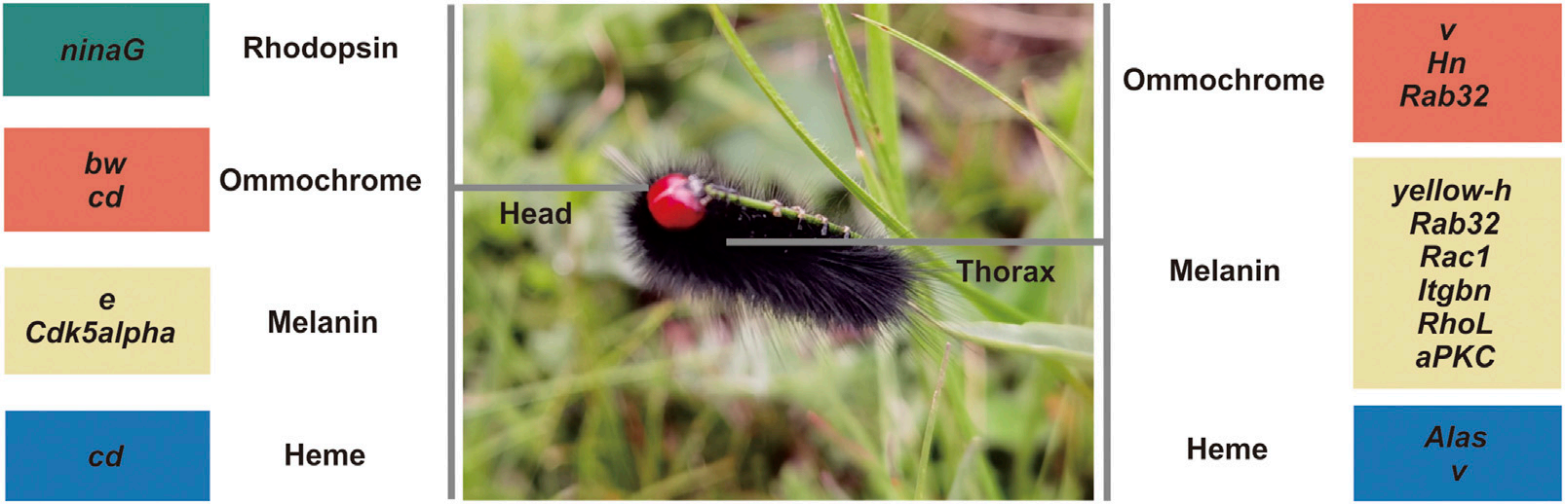
Significantly differentially expressed pigment pathway-associated genes between the head and thorax transcriptomes of *Gynaephora aureata*. Rhodopsin-associated genes, ommochrome-associated genes, melanin-associated genes, and heme-associated genes are shown in green, red, yellow, and blue boxes, respectively. *NinaG*, *bw*, *cd*, *e,* and *Cdk5alpha* are significantly upregulated in the head relative to the thorax of *G. aureata*. *V*, *Hn*, *Rab32*, *yellow-h*, *Rac1*, *Itgbn*, *RhoL*, *aPKC,* and *Alas* are significantly upregulated in the thorax relative to the head of *G. aureata*.

Eight of seventeen rhodopsin-associated genes were confirmed by RBH (Supplementary Table S2). The *ninaG* gene was significantly upregulated in the head (Supplementary Table S10; Figure 3). For ommochrome-associated genes, 19 out of 26 ommochrome-associated genes were confirmed by RBH (Supplementary Table S2). Among these, six ommochrome-associated genes, including Dihydropterin deaminase (*DhpD*), maroon-like (*mal*), Punch (*Pu*), sepia (*se*), claret (*ca*), and deep orange (*dor*), were not only related to the ommochrome biosynthesis pathway but also to the pteridine biosynthesis pathway. Two of these ommochrome-associated genes, cardinal (*cd*) and vermilion (*v*), were not only related to the ommochrome biosynthesis pathway but also to the heme biosynthesis pathway. The melanin and ommochrome biosynthesis pathways shared one gene (*Rab32*). Five key genes in the biosynthesis of ommochrome, namely, *v*, cinnabar (*cn*), *cd*, white (*w*), and scarlet (*st*), were identified by RBH (Supplementary Table S2). Three ommochrome-associated genes (*v*, *Hn,* and *Rab32*) were significantly upregulated in the thorax, and two ommochrome-associated genes, *cd* and brown (*bw*), were significantly upregulated in the head (Supplementary Table S10; Figure 3). One of seven pteridine-associated genes was confirmed by RBH (Supplementary Table S2). Additionally, 37 out of 50 melanin-associated genes were confirmed by RBH, including four key genes yellow-f, yellow-f2, yellow-h and ebony (*e*) involved in melanin synthesis (Supplementary Table S2). Six melanin-associated genes, including yellow-h, Rab32, Rac1, Integrin betanu subunit (*Itgbn*), Rho-like (*RhoL*), and atypical protein kinase C (*aPKC*), were significantly upregulated in the thorax, and two melanin-associated genes, *e* and Cyclin-dependent kinase 5 (*Cdk5alpha*), were significantly upregulated in the head (Supplementary Table S10; Figure 3). Furthermore, 12 out of 16 heme-associated genes were confirmed by RBH (Supplementary Table S2). Two heme-associated genes, Aminolevulinate synthase (*Alas*) and *v*, were significantly upregulated in the thorax, and one heme-associated gene (*cd*) was significantly upregulated in the head (Supplementary Table S10; Figure 3).

### Candidate genes related to odorant transport molecules

**FIGURE 4:**
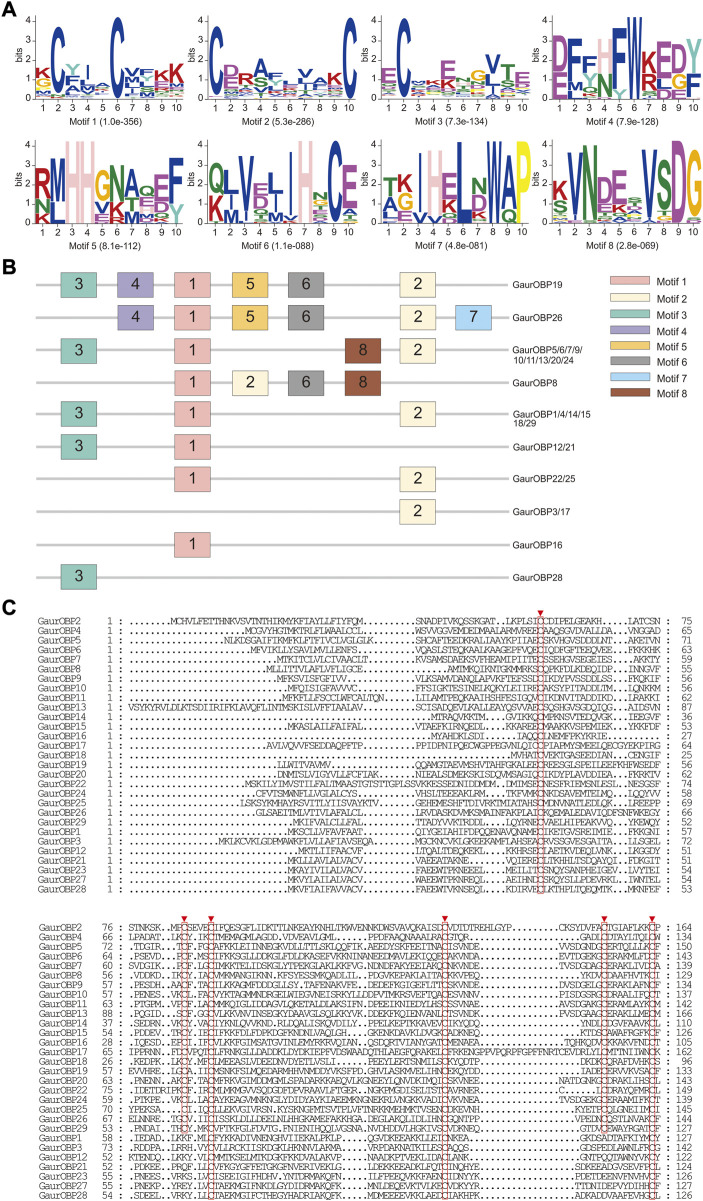
Motif pattern analysis and alignment of candidate odorant-binding proteins (OBPs) of *Gynaephora aureata*. **(A)** Eight motifs from *G. aureata* and other lepidopterans. The *E*-value for each motif is shown in parentheses. **(B)** Locations of each motif in the protein sequences. **(C)** Alignment of candidate OBPs of *G. aureata*. Highly conserved cysteine residues are marked with red borders.

In the head and thorax transcriptomes of *G. aureata*, we identified 29 OBPs with ORFs ranging from 324 to 804 bp encoding 107–267 amino acids (Supplementary Tables S11, S12). Among 29 OBPs, 18 had complete ORFs. Based on a multiple sequence alignment, 22 OBPs belonged to the typical “Classic OBP” subfamily with six conserved cysteine residues. Seven OBPs were assigned to the “Minus-C OBP” subfamily with four conserved cysteine residues (Figure 4C). The homology of 23 OBP sequences from Lepidoptera orthologs ranged from 38.98% to 88.72% and the homology of 6 OBP sequences from Diptera orthologs ranged from 31.07% to 86.33% in the NCBI database (Supplementary Table S11). MEME results revealed 8 motifs in the identified OBPs (Figure 4A). Nine OBPs had the same motif pattern, 3-1-8-2. Six OBPs had the same motif pattern, 3-1-2 (Figure 4B). Five of the 29 OBPs were significantly upregulated in the thorax and 13 in the head (Table 1).

**TABLE 1:** Summary of all significantly differential expressed olfactory-related genes between the head and thorax transcriptomes of *Gynaephora aureata*. FDR represents the corrected *p*-value.

**Gene symbol**	**Unigene ID**	**Gene name**	**Log_2_ Fold Change (thorax/head)**	**Nr annotation**	***P* value (head vs. thorax)**	**FDR (head vs. thorax)**
odorant-binding proteins (OBPs)	c123304_g1	GaurOBP3	6.45	-	1.30E-39	8.34E-38
	c87770_g1	GaurOBP15	5.44	Odorant binding protein [*Calliphora stygia*]	2.48E-09	3.90E-08
	c63775_g2	GaurOBP23	4.69	-	1.04E-06	1.20E-05
	c102377_g1	GaurOBP29	4.40	-	6.69E-06	6.80E-05
	c138562_g1	GaurOBP2	1.76	-	7.30E-52	6.30E-50
	c112026_g1	GaurOBP8	-1.10	OBP8 [*Helicoverpa armigera*]	4.10E-05	3.62E-04
	c120618_g1	GaurOBP12	-2.36	-	2.44E-80	3.78E-78
	c116221_g1	GaurOBP4	-2.66	OBPABPX, partial [*Sesamia inferens*]	2.68E-07	3.33E-06
	c125482_g1	GaurOBP5	-2.74	OBP5 [*Helicoverpa armigera*]	1.78E-12	3.68E-11
	c75840_g1	GaurOBP22	-3.00	OBP10, partial [*Sesamia inferens*]	1.23E-04	9.86E-04
	c116014_g1	GaurOBP19	-3.11	RecName: Full=General odorant-binding protein 2; Short=GOBP 2; AltName: Full=APR-10; AltName: Full=Pheromone-binding protein 10; Flags: Precursor [*Antheraea pernyi*]	4.98E-07	5.98E-06
	c80545_g1	GaurOBP7	-3.51	OBP5 [*Helicoverpa armigera*]	5.30E-65	6.21E-63
	c121370_g1	GaurOBP6	-4.09	Odorant binding protein 5 [*Spodoptera exigua*]	3.56E-21	1.21E-19
	c62166_g1	GaurOBP24	-4.22	Odorant binding protein [*Spodoptera exigua*]	1.81E-05	1.71E-04
	c110319_g1	GaurOBP11	-4.24	SexiOBP11 [*Spodoptera exigua*]	1.04E-22	3.80E-21
	c85948_g1	GaurOBP13	-4.36	Odorant-binding protein [*Helicoverpa assulta*]	0.00E+00	0.00E+00
	c115277_g1	GaurOBP20	-6.00	OBP6, partial [*Sesamia inferens*]	5.67E-15	1.39E-13
	c115219_g1	GaurOBP9	-6.88	-	2.12E-25	8.63E-24
chemosensory proteins (CSPs)	c102794_g1	GaurCSP7	1.52	Putative chemosensory protein [*Sesamia inferens*]	2.96E-32	1.53E-30
	c93001_g1	GaurCSP14	1.49	Chemosensory protein 11b [*Danaus plexippus*]	4.12E-13	8.90E-12
	c78200_g1	GaurCSP10	-1.12	Chemosensory protein [*Helicoverpa armigera*]	1.34E-16	3.63E-15
	c113883_g1	GaurCSP6	-1.51	Chemosensory protein [*Mamestra brassicae*]	1.13E-56	1.11E-54
	c110453_g1	GaurCSP11	-1.61	Chemosensory protein [*Danaus plexippus*]	5.39E-76	7.76E-74
	c112228_g1	GaurCSP8	-1.89	Chemosensory protein [*Helicoverpa assulta*]	1.76E-113	5.39E-111
	c113883_g2	GaurCSP5	-2.30	Chemosensory protein [*Mamestra brassicae*]	1.06E-120	3.51E-118
	c101910_g1	GaurCSP2	-2.57	Chemosensory protein 13 [*Papilio xuthus*]	5.54E-215	5.72E-212
	c122714_g1	GaurCSP1	-2.70	Chemosensory protein 10 [*Helicoverpa armigera*]	1.45E-88	2.66E-86
odorant receptor proteins (ORs)	c87037_g1	GaurOR4	3.68	Putative olfactory receptor 39 [*Ostrinia furnacalis*]	8.71E-07	1.01E-05
	c105845_g1	GaurOR14	2.69	Putative chemosensory receptor 9 [*Heliothis virescens*]	5.81E-04	4.00E-03
	c106937_g1	GaurOR9	2.40	Odorant receptor [*Dendrolimus kikuchii*]	5.06E-11	9.30E-10
	c117776_g1	GaurOR5	-1.79	Odorant receptor 83b [*Loxostege sticticalis*]	1.89E-06	2.09E-05
ionotropic receptors (IRs)	c89666_g3	GaurIR6	2.51	Ionotropic receptor, partial [*Helicoverpa armigera*]	1.36E-03	8.49E-03
gustatory receptors (GRs)	c165296_g1	GaurGR8	3.68	Gustatory receptor 68 [*Bombyx mori*]	3.36E-04	2.45E-03
	c92521_g1	GaurGR5	2.88	Gustatory receptor, partial [*Helicoverpa armigera*]	3.74E-05	3.33E-04
	c92124_g1	GaurGR10	1.59	Gustatory receptor 46, partial [*Bombyx mori*]	1.27E-03	8.02E-03
odorant degrading enzymes (ODEs)	c126824_g1	GaurCXE4	3.06	Odorant degrading enzyme CXE13 [*Sesamia inferens*]	3.97E-99	9.12E-97
	c111179_g1	GaurCXE5	2.41	Odorant degrading enzyme CXE13 [*Sesamia inferens*]	3.09E-89	5.75E-87
	c126077_g1	GaurCXE7	2.00	Odorant degrading enzyme CXE6, partial [*Sesamia inferens*]	6.20E-107	1.65E-104
	c134134_g1	GaurCXE3	1.23	Odorant degrading enzyme CXE18 [*Sesamia inferens*]	2.41E-19	7.43E-18
	c130873_g2	GaurCXE6	-2.09	Odorant degrading enzyme CXE13 [*Sesamia inferens*]	6.19E-62	6.87E-60
sensory neuron membrane protein genes (SNMPs)	c75543_g1	GaurSNMP2	3.31	Predicted: Sensory neuron membrane protein 2 [*Bombyx mori*]	4.10E-98	9.11E-96

**FIGURE 5:**
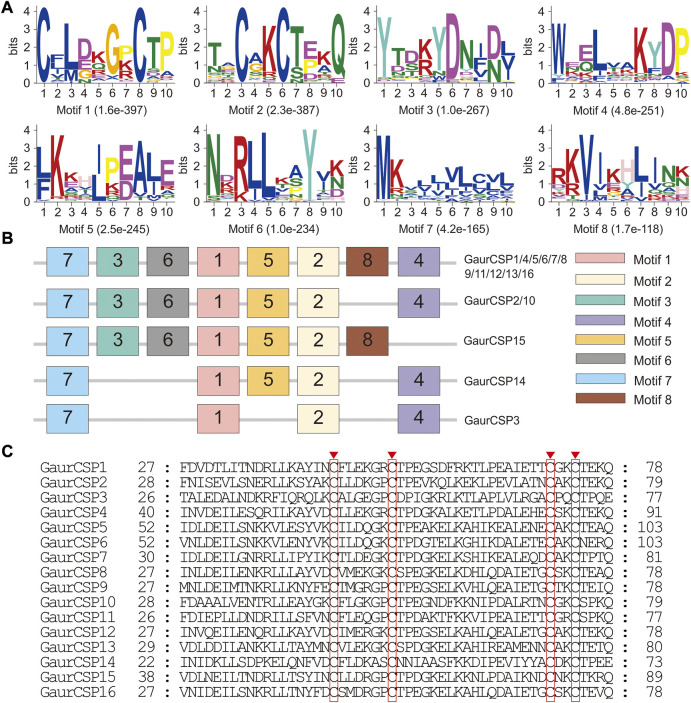
Motif pattern analysis and alignment of candidate chemosensory proteins (CSPs) of *Gynaephora aureata*. **(A)** Eight motifs from *G. aureata* and other lepidopterans. The *E*-value for each motif is shown in parentheses. **(B)** Locations of each motif in the protein sequences. **(C)** Alignment of candidate CSPs of *G. aureata*. The highly conserved cysteine residues are marked with red borders.

A total of 16 putative CSPs were identified from the head and thorax transcriptomes of *G. aureata*, of which 11 CSPs had complete ORFs (Supplementary Tables S11 and S12). The ORFs of 16 CSP genes ranged from 318 to 447 bp and encoded 105 to 148 amino acids (Supplementary Table S11). BLASTX results showed that in addition to the homology of GaurCSP14 (40.98%), the 15 CSPs had high homology (63.48%–92.73%) with lepidopteran orthologs (Supplementary Table S11). All CSPs except GaurCSP15 had signal peptides (Supplementary Table S11). All CSPs had four positionally conserved cysteine residues and the featured sequence C1-X6-7-C2-X18-C3-X2-C4 (where X indicates any amino acid) of typical insect CSPs (Figure 5C). MEME revealed 8 motifs in the identified CSPs (Figure 5A). Eleven CSPs had the same motif pattern, 7-3-6-1-5-2-8-4 (Figure 5B). Two CSPs had the same motif pattern, 7-3-6-1-5-2-4 (Figure 5B). The motif pattern of GaurCSP15 was 7-3-6-1-5-2-8 (Figure 5B). Two of the 16 CSPs were significantly upregulated in the thorax and seven were upregulated in the head (Table 1).

We identified 22 putative ORs in *G. aureata* based on sequence similarity to insect ORs (Supplementary Tables S11, S12). The genes of 22 ORs had ORFs encoding 105–336 amino acids and were predicted to have 0–5 TMDs, of which the genes encoding five ORs (GaurOR1, GaurOR2, GaurOR3, GaurOR5, and GaurOR12) had complete ORFs. The homology of OR sequences from lepidopteran orthologs ranged from 46.23% to 94.89% in the NCBI database (Supplementary Table S11 and S12). Three (GaurOR4, GaurOR9, and GaurOR14) of the 29 ORs were significantly upregulated in the thorax and one (GaurOR5) was upregulated in the head (Table 1).

The analysis of transcriptome data revealed 14 IRs (Supplementary Tables S11, S12). These IRs had ORFs ranging from 297 to 2,724 bp, encoded 98–907 amino acids (Supplementary Table S11), and had 0–4 predicted TMDs (Supplementary Table S11). Most best hit IRs shared more than 68% amino acid sequence identity with lepidopteran orthologs, whereas GaurIR6 and GaurIR8 shared 45.04% and 45.87% amino acid sequence identities, respectively (Supplementary Table S11). One (GaurIR6) of the 14 IRs was significantly upregulated in the thorax (Table 1).

We identified 12 putative GRs in *G. aureata* (Supplementary Tables S11, S12), containing 0–8 TMDs (Supplementary Table S11). The lengths of the deduced proteins for putative GR genes ranged from 106 to 455 amino acids. Of these, four of the GRs had complete ORFs (Supplementary Table S11). The BLASTX searches against the Nr database showed 32.56%–91.48% homology with sequences in other Lepidoptera (Supplementary Table S11). Three (GaurGR5, GaurGR8, and GaurGR10) of the 12 GRs were significantly upregulated in the thorax (Table 1).

We identified 12 putative ODEs in the head and thorax transcriptomes of *G. aureata* (Supplementary Tables S11, S12). The ORFs of 12 ODE genes ranged from 357 to 1785 bp and encoded 118–594 amino acids (Supplementary Table S11). Four of these ODEs had complete ORFs (Supplementary Table S11). The candidate ODEs shared 42.50%–75.47% amino acid sequence identities with other Lepidoptera ODEs in the NCBI database (Supplementary Table S11). Four (GaurCXE3, GaurCXE4, GaurCXE5, and GaurCXE7) of the 12 ODEs were significantly upregulated in the thorax and one (GaurCXE6) was upregulated in the head (Table 1).

We identified two putative SNMPs with complete ORFs, GaurSNMP1 and GaurSNMP2 (Supplementary Tables S11, S12). The ORFs of GaurSNMP1 and GaurSNMP2 were 1,560 and 1,521 bp and can encode 519 and 506 amino acids, respectively (Supplementary Table S11). The deduced GaurSNMP1 and GaurSNMP2 proteins contained two TMDs (Supplementary Table S11), conforming to the general characteristics of SNMPs. GaurSNMP1 and GaurSNMP2 had 78.65% and 60.67% homology with sequences of other Lepidoptera species (Supplementary Table S11). GaurSNMP2 was significantly upregulated in the thorax (Table 1).

## Discussion

The first transcriptomic analyses of *G. aureata* were conducted by high-throughput sequencing. Some sDEGs between the head and thorax of *G. aureata* were related to carbohydrate metabolism, lipid metabolism, epidermal protein, and detoxification. There were more upregulated sDEGs related to carbohydrate and lipid metabolism in the thorax than in the head, which may be related to the energy consumption caused by movement in the thorax. Genes encoding cuticle proteins had different expression patterns among tissues. The number of upregulated sDEGs encoding cuticle proteins was higher in the head than in the thorax, which may be due to the higher degree of ossification in head. The detoxification, immune response, and DNA repair functions of the QTP *Gynaephora* species may be related to high-altitude adaptation. Additionally, the detoxification function of the thorax may be stronger than that of the head in *G. aureata* (Supplementary Table S8).

**FIGURE 6:**
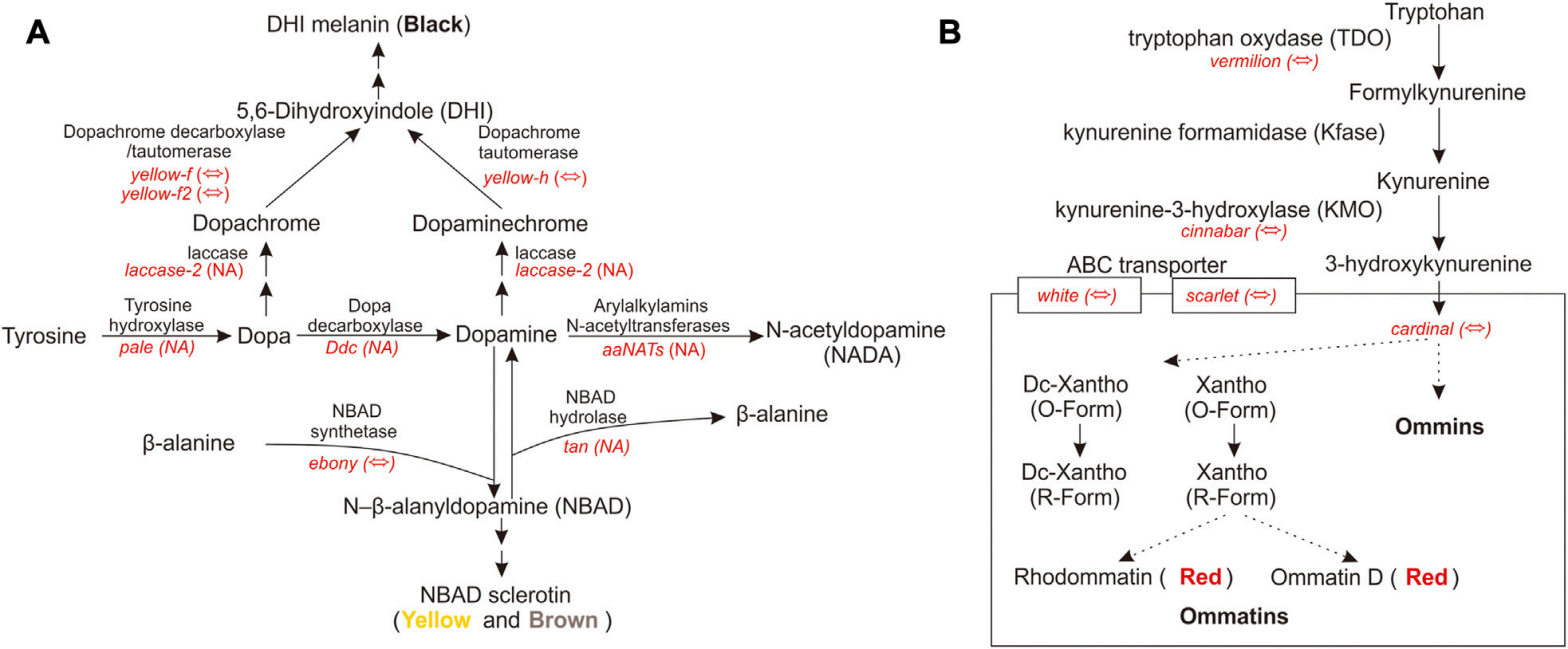
Melanin and ommochrome biosynthesis pathways (Sullivan and Sullivan, 1975; O'Hare et al., 1984; Searles and Voelker, 1986; ten Have et al., 1995; Yang, 2021; Barek et al., 2022). **(A)** Key gene products in the melanin biosynthesis pathway. **(B)** Key gene products in the ommochrome biosynthesis pathway. “⇔” indicates that the gene is supported by a reciprocal BLAST hit (RBH) analysis; “NA” indicates that the gene is either not supported by the RBH analysis or not part of the *D. melanogaster* AmiGO pigment gene set.

Pigment pathway-associated genes are divided into five categories, namely, rhodopsin, ommochrome, pteridine, melanin, and heme. The upregulated pigment pathway-associated sDEGs in the head included *ninaG*, *bw*, *cd*, *e,* and *Cdk5alpha*. The rhodopsin-associated gene *ninaG* plays a role in the biochemical pathway of responsible for the conversion of retinal to the rhodopsin chromophore 3-hydroxyretinal (Sarfare et al., 2005). The upregulated pigment pathway-associated sDEGs in the thorax included *v*, *Hn*, *Rab32*, *yellow-h*, *Rac1*, *Itgbn*, *RhoL*, *aPKC,* and *Alas*. The heme-associated gene *Alas* is the first enzyme in the heme biosynthesis pathway. The melanin-associated gene *Rab32* has a key role in the biogenesis of melanosomes and potentially other lysosome-related organelles (Wasmeier et al., 2006). There are few studies on the relationship between pigment-associated genes (*Cdk5alpha*, *bw*, *Hn*, *Rac1*, *Itgbn*, *RhoL,* and *aPKC*) and body color. Further functional experiments will be needed to link these pigment-associated genes to body color. QTP *Gynaephora* species show red head and black body hair, which are morphological features associated with high-altitude adaptation. The red and black color formation in *G. aureata* may be related to the melanin and ommochrome biosynthesis pathways. We focused on the melanin-associated genes *e* and *yellow-h* and ommochrome-associated genes *cd* and *v*. In the biosynthesis pathway of melanin, tyrosine hydroxylase encoded by *pale* hydroxylates the first tyrosine to dopa (Figure 6A). Under the action of dopamine decarboxylase (*Ddc*), dopamine is converted into dopamine (Barek et al., 2022). Dopamine under the action of NBAD synthetase (*e*) forms NBAD (*N*-β-alanyldopamine), which participates in epidermal fusion, making the epidermis yellow or light brown (Wittkopp et al., 2003a; Wittkopp et al., 2003b; Yang, 2021; Barek et al., 2022). In *G. aureata*, the *e* gene was significantly upregulated in the head (Figure 3), which may be related to the yellow appearance in the frons of the head. Phenoloxidase with wide specificity can oxidize dopa and dopamine, and convert them into quinones, which can rapidly cyclize to form amino pigments. Cuticular laccase oxidizes dopa and dopamine into corresponding quinones (Arakane et al., 2005), quinones rapidly cyclize to form amino chromes (Figure 6A) (Barek et al., 2022). In *D. melanogaster*, DCDT (dopachrome decarboxylase/tautomerases) encoded by *yellow-f* and *yellow-f2* may convert dopachrome and dopaminechrome into the same end product, DHI (5,6-dihydroxyindole) (Figure 6A) (Barek et al., 2022). However, in other insects, DCDT is recalcitrant to dopachrome. Therefore, a separate DPT (dopaminechrome tautomerase) is required (Barek et al., 2022). Recent research has shown that DPT is the product of the *yellow-h* gene in *D. melanogaster* (Figure 6A) (Barek et al., 2022). In *Papilio xuthus*, *yellow-h3* was detected at sites where melanin was deposited in the cuticle and may participate in the biosynthesis of melanin (Futahashi et al., 2011; Barek et al., 2022). In *G. aureata*, four *yellow*-related genes (*yellow*, *yellow-f*, *yellow-f2,* and *yellow-h*) were confirmed by RBH (Supplementary Table S2). *Yellow-f* and *yellow-f2* may be involved in the conversion of dopa to DHI. According to a chemical analysis, melanin in insects mainly comes from dopamine, not dopa (Sugumaran and Barek, 2016; Barek et al., 2018; Barek et al., 2022). Therefore, gradual melanin formation *via* dopamine may be an important process. *Yellow-h* was significantly upregulated in the thorax of *G. aureata* (Supplementary Table S10; Figure 3). *Yellow-h* may be related to the transformation of dopamine into DHI, producing melanin to form the black body color of *G. aureata*. Therefore, *yellow-h* may be crucial in the formation of melanin in *G. aureata*; however, further studies are still needed to confirm this.

In the biosynthesis of ommochrome, tryptophan forms the precursor of the eye pigment 3-hydroxycaninurine under the action of tryptophan oxygenase (TDO), encoded by *vermilion*, kynurenine formamidase (Kfase), and kynurenine-3-hydroxylase (KMO) encoded by *cinnabar*, and it is transferred to pigment granules to form a heterodimer by the ABC transporter *w* and *scarlet* (*st*) (Figure 6B) (Sullivan and Sullivan, 1975; O'Hare et al., 1984; Searles and Voelker, 1986; ten Have et al., 1995; Yang, 2021). Ommochromes are divided into ommatins and ommins. The molecular mechanism underlying the transition from 3-hydroxykynurenine to ommochrome is largely unknown. *Cd* is important in the formation of eye pigments in *B. mori* (Osanai-Futahashi et al., 2016). In *G. aureata*, five key genes involved in the biosynthesis of ommochrome, namely, *v*, *cn*, *cd*, *w,* and *st*, were identified (Supplementary Table S2). Ommin A forms a purple phenotype, decarboxylated xanthommatin, decarboxylated dihydroxanthommatin, rhodommatin, and ommatin D form a red phenotype (Yang, 2021). In *G. aureata*, the *cd* gene was significantly upregulated in the head (Supplementary Table S10; Figure 3), which may be related to the red head of the species. The *v* gene was significantly upregulated in the thorax (Supplementary Table S10; Figure 3), suggesting that ommochromes formed in the thorax. The black phenotype of the thorax of *G. aureata* is the result of multiple pigments; however, further research is still needed to determine the detailed mechanisms.

In insects, olfaction plays an important physiological function by sensing chemical clues in the environment (Peng et al., 2022). Insects may adjust their olfactory system to adapt to changes in biotic (such as plant or host phenotype and genotype) and abiotic factors (Linz et al., 2013; Claudianos et al., 2014). The change in feeding habits of QTP *Gynaephora* species is an adaptation of the olfactory system to the environment of the QTP. In this study, 107 olfactory-related genes were identified in *G. aureata* (Supplementary Table S11; Figure 2), including OBPs, CSPs, ORs, IRs, GRs, ODEs, and SNMPs. As the first step in odor recognition, OBPs and CSPs can bind to and transport lipophilic odorant molecules (Wanner et al., 2005; Pelosi et al., 2006; Guo et al., 2011; Dippel et al., 2014; Pelosi et al., 2014; Pelosi et al., 2018; Liu et al., 2020). We identified 29 OBPs (Supplementary Table S11; Figure 2), which was fewer than the numbers in *Spodoptera littoralis* (49) and *B. mori* (46) (Gong et al., 2009; Vieira and Rozas, 2011; Walker et al., 2019) but similar to estimates in other species in Lepidoptera, such as *Spodoptera frugiperda* (25) and *Chilo suppressalis* (26) (Cao et al., 2014; Qiu et al., 2020). A total of 16 CSPs were identified, similar to corresponding counts in the lepidopterans *Plodia interpunctella* (15) and *Galleria mellonella* (18) (Jia et al., 2018; Jiang et al., 2021). The motif patterns of different OBPs and CSPs diff among insects. The most conserved motif pattern of OBPs was 3-1-8-2, and the most conserved motif pattern of CSPs was 7-3-6-1-5-2-8-4 in *G. aureata*. This showed that OBPs and CSPs may have conserved functions in odor recognition. Different motif patterns provide evidence for possible functional differentiation. Chemosensory receptor protein families are involved in the detection and transduction of odorant signals, mainly including ORs, IRs, and GRs. We identified 22 ORs (Supplementary Table S11; Figure 2), fewer than estimates in *S. littoralis* (60) (Walker et al., 2019), which may be related to the sequencing method and sequencing depth. We identified 12 GRs and 14 IRs (Supplementary Table S11; Figure 2). The number of GRs was similar to that in *Peridroma saucia* (10) (Sun Y. L. et al., 2020). The number of IRs was similar to counts in *S. littoralis* (17) and *B. mori* (18) (Croset et al., 2010; Walker et al., 2019). Carboxylesterases (CXEs) are among the most important ODEs. We identified 12 CXEs (Supplementary Table S11; Figure 2), which was fewer than the numbers in *S. littoralis* (20) (Durand et al., 2011; Liu et al., 2019), *Spodoptera litura* (24) (Zhang et al., 2016), and *Semia inferens* (20) (Zhang et al., 2014) but similar to the number in *Cydia pomonella* (12) (Huang et al., 2016; Liu et al., 2019). We identified two SNMPs (Supplementary Table S11; Figure 2), similar to count sin *S. litura* (2) (Zhang et al., 2014) and *B. mori* (Walker et al., 2019; Xu et al., 2021). Host finding relies on the olfactory system for the detection of environmental signals (Gols et al., 2012; Suh et al., 2014; Ballesteros et al., 2019). IRs play a key role in sensing different odors, such as acids, salts, ammonia, and aldehyde (Rytz et al., 2013; Chen et al., 2015; Enjin et al., 2016; Knecht et al., 2016; Frank et al., 2017; Zhang et al., 2019). GRs mainly detect sugar, bitterness, and pheromones, which have important functions in finding hosts (Clyne et al., 2000; Park and Kwon, 2011; Mang et al., 2016). OBPs and ORs play key roles in host preferences. Therefore, the differences in expression levels of different olfactory genes may be related to the recognition of different hosts; however, further research is still needed to verify this.

To explore the genetic basis of high-altitude adaptation in QTP *Gynaephora* species, we performed the sequencing, assembly and annotation of the head and thorax transcriptomes of *G. aureata* and identified 73 pigment pathway and 107 olfactory-related genes. Pigment pathway-associated genes are related to body color adaptation to high-altitude environments (low temperatures and high UV levels). Olfactory-related genes are related to the adaptation of *G. aureata* to the abundant plant resources in the QTP. Pigment pathway and olfactory-related genes provide insight into the adaptation of QTP *Gynaephora* species to high altitudes, including the genetic basis of body color and changes in food habits. A reference genome is not available for QTP *Gynaephora* species. All analyses in this study were based on the head and thorax transcriptomes of *G. aureata*. In the future, pigment pathway and olfactory-related genes should be identified by full-genome analyses. In addition, functional analyses of key melanin-associated *yellow-h* and ommochrome-associated *cd* genes are needed in the future.

## Conclusion

In this study, transcriptomic analyses of *G. aureata* were conducted by high-throughput sequencing for the first time to clarify the molecular basis of key traits related to survival in the QTP. We detected sDEGs related to carbohydrate metabolism, lipid metabolism, epidermal proteins, and detoxification. Additionally, we identified 73 genes involved in pigment pathways and 107 olfactory-related genes in *G. aureata*. The melanin-associated gene *yellow-h* and the ommochrome-associated gene *cd* might play important roles in the formation of black and red body colors of *G. aureata*, respectively. However, further functional assays of *yellow-h* and *cd* are needed in the future. These results provide new insights into high-altitude adaptation of *Gynaephora* in the QTP, providing a basis for the development of new control strategies for these pests.

## Data Availability

The original contributions presented in the study are publicly available. This data can be found here: SRR22475456-61.
