# Supplementary material for: Differential gene expression patterns between the head and thorax of Gynaephora aureata are associated with high-altitude adaptation
Source: Front Genet. 2023 Apr 18;14:1137618. doi: 10.3389/fgene.2023.1137618 (PMC10151491; doi:10.3389/fgene.2023.1137618)
Supplement: Supplementary file 1 [file DataSheet1.zip › Table S1.docx]

**Table S1. Primers used for the quantitative real-time PCR (qRT-PCR) analysis of *Gynaephora aureata*.**

| **Gene ID** | **Gene name** | **Abbreviation** | **Primer name** | **Primer sequence (5’ to 3’)** | **Product size (bp)** | **Tm (°C)** |
| --- | --- | --- | --- | --- | --- | --- |
| c125087_g1 | *Alpha-tubulin* | *C* | c125087F | CTAATGCGTCTGATTCCTGTT | 160 | 50 |
|  |  |  | c125087R | CCTCGTGCGTAGTTGTTC |  |  |
| c1042_g1 | *Fatty acid binding protein* | *D* | c1042F | AACACAGTTATCCAGACAGTCAA | 115 | 53 |
|  |  |  | c1042R | CAACGCCATCCCATCCTT |  |  |
| c62166_g1 | *Odorant binding protein* | *OBP* | c62166F | GGAGCATTACATTCTCACCTACC | 90 | 60 |
|  |  |  | c62166R | GGCAACCGTATCCGATGG |  |  |
| c115942_g2 | *Carbohydrate digestion and absorption* | *CDA* | CDAF2 | TCAACTCCAGCATCTAAGC | 120 | 55 |
|  |  |  | CDAR2 | AGGTACAACAGTCCAACATT |  |  |
| c118047_g1 | *Developmental pigmentation* | *DP* | DPF | TAATAGGTTCAATCATCATC | 110 | 55 |
|  |  |  | DPR | AATAAGCACCGTTAGATA |  |  |
| c130659_g2 | *Dihydropteridine reductase* | *DR* | DRF | ATACAACAATGCGTCCACTA | 150 | 55 |
|  |  |  | DRR | GGTCCAGTTAGTTAGTTCGTAT |  |  |
| Internal reference gene | *Elongation factor 1 alpha* | *EF-1α* | EF1αF | CCCGCCAACATCACCACT | 130 | 60 |
|  |  |  | EF1αR | CGTAACCACGACGCAACTCC |  |  |
