# Supplementary material for: Differential gene expression patterns between the head and thorax of Gynaephora aureata are associated with high-altitude adaptation
Source: Front Genet. 2023 Apr 18;14:1137618. doi: 10.3389/fgene.2023.1137618 (PMC10151491; doi:10.3389/fgene.2023.1137618)
Supplement: Supplementary file 1 [file DataSheet1.zip › Table S10.docx]

**Table S10. Summary of all pigment pathway-associated genes significantly differentially expressed between the head and thorax transcriptomes of *Gynaephora aureata*. FDR represents the corrected *P*-value.**

| **Pigment pathway** | **Gene symbol** | **Gene name** | **Unigene ID** | **Up/down** | **Log_2_ Fold Change (thorax/head)** | ***P* value**  **(head vs. thorax)** | **FDR**  **(head vs. thorax)** |
| --- | --- | --- | --- | --- | --- | --- | --- |
| Rhodopsin | *ninaG* | *ninaG* | c131440_g1 | down | -1.70 | 6.23E-04 | 4.25E-03 |
| Ommochrome | *bw* | *brown* | c134995_g1 | down | -1.87 | 7.40E-20 | 2.34E-18 |
| Ommochrome | *Hn* | *Henna* | c120137_g1 | up | 1.02 | 2.64E-27 | 1.15E-25 |
| Ommochrome & Melanin “granule group” | *Rab32* | *Rab32* | c126401_g1 | up | 1.00 | 1.16E-22 | 4.23E-21 |
| Ommochrome & Heme “granule group” | *cd* | *cardinal* | c138628_g1 | down | -2.03 | 1.84E-95 | 3.88E-93 |
| Ommochrome & Heme “granule group” | *v* | *vermilion* | c116674_g1 | up | 1.68 | 1.33E-66 | 1.60E-64 |
| Melanin | *Rac1* | *Rac1* | c129363_g1 | up | 1.19 | 4.77E-35 | 2.68E-33 |
| Melanin | *Cdk5alpha* | *Cdk5 activator-like protein* | c136518_g1 | down | -1.56 | 1.81E-10 | 3.16E-09 |
| Melanin | *Itgbn* | *Integrin betanu subunit* | c133644_g1 | up | 1.71 | 2.16E-53 | 1.95E-51 |
| Melanin | *RhoL* | *Rho-like* | c121101_g1 | up | 1.44 | 1.39E-54 | 1.30E-52 |
| Melanin | *yellow-h* | *yellow-h* | c136149_g1 | up | 1.06 | 8.88E-07 | 1.03E-05 |
| Melanin | *e* | *ebony* | c135749_g1 | down | -3.79 | 8.71E-158 | 5.16E-155 |
| Melanin | *aPKC* | *atypical protein kinase C* | c132766_g1 | up | 1.31 | 4.34E-26 | 1.81E-24 |
| Heme | *Alas* | *Aminolevulinate synthase* | c126962_g1 | up | 1.01 | 2.73E-33 | 1.46E-31 |
