# Supplementary material for: Differential gene expression patterns between the head and thorax of Gynaephora aureata are associated with high-altitude adaptation
Source: Front Genet. 2023 Apr 18;14:1137618. doi: 10.3389/fgene.2023.1137618 (PMC10151491; doi:10.3389/fgene.2023.1137618)
Supplement: Supplementary file 1 [file DataSheet1.zip › Table S11.docx]

**Table S11. Details for candidate odorant-binding protein (OBPs), chemosensory proteins (CSPs), ionotropic receptors (IRs), gustatory receptors (GRs), olfactory receptors (ORs), odorant degrading enzymes (ODEs) and sensory neuron membrane proteins (SNMPs) of *Gynaephora aureata*. TMD represents the number of transmembrane domains.**

| **Gene symbol** | **Unigene ID** | **Gene name** | **Length (bp)** | **ORF (nt/aa)** | **Full length** | **Signal peptide** | **TMD** | **BLAXTX best hit (Reference/Name/Species)** | **Identity (%)** | **BLASTN best hit (Reference/Name/Species)** | **Identity (%)** |
| --- | --- | --- | --- | --- | --- | --- | --- | --- | --- | --- | --- |
| odorant-binding proteins (OBPs) | c124855_g2 | GaurOBP1 | 1361 | 399 \| 132 | Yes | No | 0 | gb\|AGR39568.1\| odorant binding protein 5 [*Agrotis ipsilon*] | 53.44 | odorant binding protein 5 [*Agrotis ipsilon*] | 53.44 |
|  | c138562_g1 | GaurOBP2 | 1359 | 513 \| 170 | Yes | No | 0 | gb\|AWT22229.1\| odorant-binding protein 10 [*Mythimna separata*] | 61.90 | odorant-binding protein 10 [*Mythimna separata*] | 61.90 |
|  | c123304_g1 | GaurOBP3 | 1345 | 504 \| 167 | Yes | No | 0 | gb\|AKT26499.1\| odorant binding protein 22 [*Spodoptera exigua*] | 72.86 | odorant binding protein 22 [*Spodoptera exigua*] | 72.86 |
|  | c116221_g1 | GaurOBP4 | 1180 | 438 \| 145 | Yes | No | 0 | ref\|XP_026744800.1\| general odorant-binding protein 69a-like [*Trichoplusia ni*] | 72.32 | OBPABPX, partial [*Sesamia inferens*] | 71.05 |
|  | c125482_g1 | GaurOBP5 | 1028 | 480 \| 159 | 5' missing | No | 0 | gb\|ASA40067.1\| odorant-binding protein 31 [*Helicoverpa armigera*] | 49.33 | odorant-binding protein 31 [*Helicoverpa armigera*] | 49.33 |
|  | c121370_g1 | GaurOBP6 | 1010 | 468 \| 155 | Yes | No | 0 | gb\|AGH70101.1\| odorant binding protein 5 [*Spodoptera exigua*] | 45.71 | odorant binding protein 5 [*Spodoptera exigua*] | 45.71 |
|  | c80545_g1 | GaurOBP7 | 910 | 447 \| 148 | Yes | Yes | 0 | gb\|AKT26503.1\| odorant binding protein 26 [*Spodoptera exigua*] | 65.77 | general odorant-binding protein 28a-like [*Spodoptera litura*] | 64.00 |
|  | c112026_g1 | GaurOBP8 | 894 | 804 \| 267 | Yes | No | 0 | gb\|AZB49383.1\| odorant-binding protein 2 [*Heortia vitessoides*] | 66.42 | OBP8 [*Helicoverpa armigera*] | 65.41 |
|  | c115219_g1 | GaurOBP9 | 858 | 444 \| 147 | Yes | No | 0 | ref\|XP_021194655.1\| general odorant-binding protein 69a-like [*Helicoverpa armigera*] | 52.00 | general odorant-binding protein 69a-like [*Helicoverpa armigera*] | 52.19 |
|  | c108905_g2 | GaurOBP10 | 744 | 444 \| 147 | Yes | No | 0 | gb\|ALZ45421.1\| odorant binding protein 6 [*Athetis dissimilis*] | 60.81 | general odorant-binding protein 28a [*Helicoverpa armigera*] | 55.48 |
|  | c110319_g1 | GaurOBP11 | 739 | 462 \| 153 | Yes | No | 0 | gb\|AGP03457.1\| SexiOBP11 [*Spodoptera exigua*] | 70.13 | SexiOBP11 [*Spodoptera exigua*] | 70.13 |
|  | c120618_g1 | GaurOBP12 | 714 | 402 \| 133 | Yes | No | 0 | gb\|AGH70105.1\| odorant binding protein 9 [*Spodoptera exigua*] | 88.72 | odorant-binding protein 17 [*Helicoverpa armigera*] | 89.63 |
|  | c85948_g1 | GaurOBP13 | 707 | 528 \| 175 | 5' missing | No | 0 | gb\|AKT26503.1\| odorant binding protein 26 [*Spodoptera exigua*] | 43.62 | odorant-binding protein 31 [*Helicoverpa armigera*] | 47.20 |
|  | c83210_g1 | GaurOBP14 | 705 | 366 \| 121 | Yes | No | 0 | gb\|ASA40075.1\| odorant-binding protein 37 [*Helicoverpa assulta*] | 78.51 | general odorant-binding protein lush-like [*Spodoptera litura*] | 76.86 |
|  | c87770_g1 | GaurOBP15 | 675 | 423 \| 140 | Yes | Yes | 0 | gb\|AID61300.1\| odorant binding protein [*Calliphora stygia*] | 86.33 | odorant binding protein (OBP7) mRNA, complete cds [*Calliphora stygia*] | 86.19 |
|  | c80895_g1 | GaurOBP16 | 663 | 339 \| 112 | Yes | No | 0 | gb\|KOB73305.1\| Odorant binding protein [*Operophtera brumata*] | 74.11 | odorant binding protein 32, partial [*Spodoptera litura*] | 52.38 |
|  | c9568_g1 | GaurOBP17 | 615 | 570 \| 189 | 5' missing | No | 0 | gb\|AKT26497.1\| odorant binding protein 19 [*Spodoptera exigua*] | 38.98 | odorant-binding protein 19 [*Helicoverpa assulta*] | 31.71 |
|  | c133725_g3 | GaurOBP18 | 595 | 324 \| 107 | Yes | No | 0 | gb\|AGS36756.1\| OBP16 [*Sesamia inferens*] | 77.97 | OBP16, partial [*Sesamia inferens*] | 77.97 |
|  | c116014_g1 | GaurOBP19 | 588 | 471 \| 156 | 5' missing | No | 0 | gb\|AGJ83353.1\| general odorant-binding protein 2 [*Dendrolimus kikuchii*] | 81.41 | general odorant-binding protein 2 [*Helicoverpa armigera*] | 79.49 |
|  | c115277_g1 | GaurOBP20 | 557 | 471 \| 156 | 5' missing | No | 0 | ref\|XP_021194654.1\| general odorant-binding protein 69a-like [*Helicoverpa armigera*] | 62.69 | general odorant-binding protein 69a-like [*Helicoverpa armigera*] | 62.69 |
|  | c111192_g1 | GaurOBP21 | 524 | 408 \| 135 | Yes | No | 0 | ref\|XP_039959060.1\| general odorant-binding protein 99a-like [*Bactrocera tryoni*] | 31.07 | general odorant-binding protein 99a [*Lucilia cuprina*] | 35.82 |
|  | c75840_g1 | GaurOBP22 | 521 | 489 \| 162 | Yes | No | 0 | gb\|AGS36751.1\| OBP10 [*Sesamia inferens*] | 66.97 | OBP10, partial [*Sesamia inferens*] | 66.97 |
|  | c63775_g2 | GaurOBP23 | 515 | 405 \| 134 | Yes | No | 0 | gb\|AID61318.1\| odorant binding protein [*Calliphora stygia*] | 44.09 | general odorant-binding protein 99a-like [*Lucilia cuprina*] | 42.52 |
|  | c62166_g1 | GaurOBP24 | 523 | 453 \| 150 | 5' missing | No | 0 | gb\|ADY17886.1\| odorant binding protein [*Spodoptera exigua*] | 72.41 | OBP3, partial [*Sesamia inferens*] | 75.42 |
|  | c109579_g1 | GaurOBP25 | 497 | 471 \| 156 | 5' missing | Yes | 0 | gb\|AGS36743.1\| OBP1 [*Sesamia inferens*] | 83.96 | OBP1 [*Sesamia inferens*] | 83.96 |
|  | c87626_g1 | GaurOBP26 | 494 | 480 \| 159 | 5' missing | No | 0 | ref\|XP_026739057.1\| pheromone-binding protein [*Trichoplusia ni*] | 65.10 | pheromone binding protein-1 [*Bombyx mori*] | 60.40 |
|  | c63775_g1 | GaurOBP27 | 460 | 423 \| 140 | 3' missing | No | 0 | gb\|AID61318.1\| odorant binding protein [*Calliphora stygia*] | 51.82 | odorant binding protein [*Calliphora stygia*] | 51.82 |
|  | c141867_g1 | GaurOBP28 | 445 | 387 \| 128 | 3' missing | No | 0 | gb\|AID61318.1\| odorant binding protein [*Calliphora stygia*] | 74.34 | general odorant-binding protein 99a-like [*Lucilia cuprina*] | 71.68 |
|  | c102377_g1 | GaurOBP29 | 440 | 411 \| 136 | 3' missing | No | 0 | ref\|XP_037823833.1\| general odorant-binding protein 99a [*Lucilia sericata*] | 76.61 | general odorant-binding protein 99a [*Lucilia sericata*] | 76.81 |
| chemosensory proteins (CSPs) | c122714_g1 | GaurCSP1 | 2587 | 369 \| 122 | Yes | Yes | 0 | gb\|AZB49399.1\| chemosensory protein 7 [*Heortia vitessoides*] | 88.79 | chemosensory protein 10 [*Mythimna separata*] | 86.92 |
|  | c101910_g1 | GaurCSP2 | 2024 | 366 \| 122 | Yes | Yes | 0 | gb\|AWT22257.1 chemosensory protein 9 [*Mythimna separata*] | 90.16 | Histia rhodope CSP2 | 74.92 |
|  | c112249_g2 | GaurCSP3 | 1347 | 318 \| 105 | Yes | Yes | 0 | gb\|AKT26494.1\| chemosensory protein 20 [*Spodoptera exigua*] | 84.11 | chemosensory protein 5 [*Agrotis ipsilon*] | 83.18 |
|  | c121905_g2 | GaurCSP4 | 1206 | 417 \| 138 | 5' missing | Yes | 0 | gb\|AIW65099.1\| chemosensory protein [*Helicoverpa armigera*] | 83.33 | chemosensory protein 14 variant [*Bombyx mori*] | 91.36 |
|  | c113883_g2 | GaurCSP5 | 1122 | 447\| 148 | 5' missing | Yes | 0 | gb\|ALT31605.1\| chemosensory protein 23 [*Cnaphalocrocis medinalis*] | 74.22 | chemosensory protein 9 [*Helicoverpa armigera*] | 67.97 |
|  | c113883_g1 | GaurCSP6 | 1111 | 447\| 148 | 5' missing | Yes | 0 | gb\|AWT22252.1\| chemosensory protein 2 [*Mythimna separata*] | 70.63 | *Oedaleus asiaticus* chemosensory protein 8 (CSP8) | 90.48 |
|  | c102794_g1 | GaurCSP7 | 980 | 381 \| 126 | Yes | Yes | 0 | gb\|AND82443.1\| chemosensory protein 1 [*Athetis dissimilis*] | 72.44 | *Carpasna sasakii* chemosensory protein 10 | 74.73 |
|  | c112228_g1 | GaurCSP8 | 921 | 387 \| 128 | Yes | Yes | 0 | gb\|AAK53762.1\| chemosensory protein [*Helicoverpa armigera*] | 92.73 | chemosensory protein 13 [*Helicoverpa armigera*] | 91.82 |
|  | c94725_g1 | GaurCSP9 | 908 | 381 \| 126 | Yes | Yes | 0 | gb\|QIJ45719.1\| chemosensory protein [*Glyphodes pyloalis*] | 65.74 | chemosensory protein 1 [*Conogethes punctiferalis*] | 65.09 |
|  | c78200_g1 | GaurCSP10 | 833 | 372 \| 123 | Yes | Yes | 0 | gb\|ASA40088.1\| chemosensory protein 26 [*Helicoverpa armigera*] | 72.36 | chemosensory protein 26 [*Helicoverpa armigera*] | 72.36 |
|  | c110453_g1 | GaurCSP11 | 787 | 363 \| 120 | Yes | Yes | 0 | gb\|AEX07267.1\| CSP6 [*Helicoverpa armigera*] | 67.54 | CSP6 [*Helicoverpa armigera*] | 67.54 |
|  | c47681_g1 | GaurCSP12 | 780 | 354 \| 117 | Yes | Yes | 0 | gb\|QIJ45719.1\| chemosensory protein [*Glyphodes pyloalis*] | 63.48 | chemosensory protein 17 [*Ectropis obliqua*] | 62.61 |
|  | c145799_g1 | GaurCSP13 | 684 | 378 \| 125 | Yes | Yes | 0 | gb\|ALT31609.1\| chemosensory protein 27 [*Cnaphalocrocis medinalis*] | 66.94 | chemosensory protein 27, partial [*Cnaphalocrocis medinalis*] | 66.94 |
|  | c93001_g1 | GaurCSP14 | 663 | 345 \| 114 | Yes | Yes | 0 | gb\|ASA40081.1\| chemosensory protein 20 [*Helicoverpa assulta*] | 40.98 | chemosensory protein 20 [*Helicoverpa armigera*] | 44.55 |
|  | c146323_g1 | GaurCSP15 | 465 | 348 \| 115 | 5' missing | No | 0 | dbj\|BAV56812.1\| chemosensory protein 8 [*Ostrinia furnacalis*] | 70.37 | chemosensory protein 8 [*Ostrinia furnacalis*] | 70.37 |
|  | c78057_g1 | GaurCSP16 | 429 | 333 \| 110 | 3' missing | Yes | 0 | gb\|AAF71289.1\| chemosensory protein [*Mamestra brassicae*] | 63.96 | chemosensory protein 13 [*Helicoverpa armigera*] | 65.26 |
| odorant receptor proteins (ORs) | c121158_g1 | GaurOR1 | 1805 | 675 \| 224 | Yes | No | 3 | ref\|XP_021196261.1\| odorant receptor Or1-like [*Helicoverpa armigera*] | 64.76 | odorant receptor Or1-like [*Helicoverpa armigera*] | 64.76 |
|  | c114549_g1 | GaurOR2 | 1491 | 600 \| 199 | Yes | No | 2 | gb\|QNS36198.1\| olfactory receptor 4 [*Mythimna separata*] | 60.83 | putative olfactory receptor 12 [*Spodoptera litura*] | 58.08 |
|  | c120471_g1 | GaurOR3 | 1312 | 1011 \| 336 | Yes | No | 5 | ref\|XP_021194498.1\| odorant receptor 46a-like [*Helicoverpa armigera*] | 71.30 | odorant receptor 46a-like [*Helicoverpa armigera*] | 71.30 |
|  | c87037_g1 | GaurOR4 | 1077 | 975 \| 324 | 3' missing | No | 5 | ref\|XP_035459442.1\| odorant receptor 94a-like [*Spodoptera frugiperda*] | 50.76 | odorant receptor 4-like [*Spodoptera litura*] | 50.78 |
|  | c117776_g1 | GaurOR5 | 1045 | 429 \| 142 | Yes | No | 3 | gb\|AZT78922.2\| odorant receptor ORco [*Dioryctria abietella*] | 94.89 | olfactory receptor OR83b [*Helicoverpa armigera*] | 97.08 |
|  | c139106_g2 | GaurOR6 | 841 | 642 \| 213 | 3' missing | No | 2 | gb\|QPX50363.1\| odorant receptor [*Helicoverpa armigera*] | 59.75 | odorant receptor [*Helicoverpa armigera*] | 59.75 |
|  | c105629_g1 | GaurOR7 | 831 | 831 \| 276 | 5', 3' missing | No | 4 | gb\|ACC63238.1\| olfactory receptor 10 [*Helicoverpa armigera*] | 60.92 | olfactory receptor 10, partial [*Helicoverpa armigera*] | 60.92 |
|  | c102101_g1 | GaurOR8 | 804 | 801 \| 266 | 5', 3' missing | No | 5 | gb\|QNS36227.1\| olfactory receptor 36 [*Mythimna separata*] | 62.06 | olfactory receptor 36 [*Mythimna separata*] | 62.06 |
|  | c106937_g1 | GaurOR9 | 770 | 615 \| 204 | 5' missing | No | 2 | ref\|XP_022824679.1\| odorant receptor 67c-like [*Spodoptera litura*] | 70.31 | odorant receptor 67c-like [*Spodoptera litura*] | 70.31 |
|  | c108565_g1 | GaurOR10 | 697 | 423 \| 140 | 5' missing | No | 3 | gb\|QMS80321.1\| odorant receptor [*Histia rhodope*] | 74.81 | odorant receptor 13a-like [*Spodoptera litura*] | 74.81 |
|  | c108922_g1 | GaurOR11 | 650 | 363 \| 120 | 5' missing | Yes | 1 | gb\|QNS36220.1\| olfactory receptor 23 [*Mythimna separata*] | 75.00 | olfactory receptor 23 [*Mythimna separata*] | 73.39 |
|  | c122933_g2 | GaurOR12 | 641 | 363 \| 120 | Yes | No | 2 | gb\|QPX50372.1\| odorant receptor [*Helicoverpa armigera*] | 90.51 | odorant receptor 4-like [*Helicoverpa armigera*] | 90.51 |
|  | c39949_g1 | GaurOR13 | 602 | 600 \| 199 | 3' missing | No | 2 | ref\|XP_022816679.1\| odorant receptor 10a-like [*Spodoptera litura*] | 71.00 | odorant receptor 10a-like [*Spodoptera litura*] | 71.00 |
|  | c105845_g1 | GaurOR14 | 558 | 555 \| 184 | 5', 3' missing | No | 2 | ref\|XP_021191840.1\| odorant receptor 4-like [*Helicoverpa armigera*] | 59.24 | odorant receptor 4-like [*Helicoverpa armigera*] | 59.24 |
|  | c60654_g1 | GaurOR15 | 534 | 531 \| 176 | 5', 3' missing | No | 3 | gb\|AJD81553.1\| olfactory receptor 15 [*Helicoverpa assulta*] | 50.80 | olfactory receptor 15, partial [*Helicoverpa assulta*] | 56.80 |
|  | c8367_g1 | GaurOR16 | 531 | 318 \| 105 | 3' missing | No | 1 | gb\|QNS36229.1\| olfactory receptor 38 [*Mythimna separata*] | 53.39 | olfactory receptor 38 [*Mythimna separata*] | 53.39 |
|  | c148515_g1 | GaurOR17 | 524 | 354 \| 117 | 3' missing | Yes | 0 | ref\|XP_026730844.1\| odorant receptor 4-like [*Trichoplusia ni*] | 46.23 | olfactory receptor 4 [*Mythimna separata*] | 45.37 |
|  | c56717_g1 | GaurOR18 | 494 | 384 \| 127 | 5' missing | No | 2 | gb\|AIG51898.1\| odorant receptor [*Helicoverpa armigera*] | 77.17 | odorant receptor [*Helicoverpa armigera*] | 77.17 |
|  | c39949_g2 | GaurOR19 | 473 | 351 \| 116 | 5' missing | Yes | 0 | ref\|XP_035435381.1\| odorant receptor 10a-like [*Spodoptera frugiperda*] | 78.45 | odorant receptor 10a-like [*Spodoptera frugiperda*] | 78.45 |
|  | c160229_g1 | GaurOR20 | 465 | 354 \| 117 | 3' missing | No | 1 | gb\|ALM26194.1\| odorant receptor 6 [*Athetis dissimilis*] | 51.09 | odorant receptor, partial [*Helicoverpa armigera*] | 43.43 |
|  | c89538_g1 | GaurOR21 | 442 | 438 \| 145 | 5', 3' missing | No | 2 | gb\|QPX50362.1\| odorant receptor [*Helicoverpa armigera*] | 57.04 | *Manduca sexta* mRNA for Olfactory receptor 26 (OR-26 gene) | 94.59 |
|  | c50962_g1 | GaurOR22 | 388 | 327 \| 108 | 5' missing | Yes | 1 | gb\|ARO76427.1\| odorant receptor 21 [*Conogethes punctiferalis*] | 57.29 | odorant receptor 21 [*Conogethes punctiferalis*] | 57.29 |
| ionotropic receptors (IRs) | c124465_g1 | GaurIR1 | 3074 | 2514 \| 837 | 5' missing | No | 3 | gb\|AJD81628.1\| ionotropic receptor 25a [*Helicoverpa assulta*] | 91.45 | ionotropic receptor 25a isoform X1 [*Helicoverpa armigera*] | 91.45 |
|  | c132989_g1 | GaurIR2 | 2770 | 2724 \| 907 | 5' missing | No | 4 | gb\|ARB05665.1\| ionization receptor 8a [*Mythimna separata*] | 81.33 | ionization receptor 8a [*Helicoverpa armigera*] | 79.73 |
|  | c125651_g1 | GaurIR3 | 2162 | 435 \| 144 | Yes | Yes | 1 | ref\|XP_021200448.1\| ionotropic receptor 21a [*Helicoverpa armigera*] | 70.18 | ionotropic receptor 21a [*Helicoverpa armigera*] | 70.18 |
|  | c104535_g1 | GaurIR4 | 1176 | 1017 \| 338 | 5' missing | No | 3 | gb\|QNS36192.1\| ionotropic receptor 75p [*Mythimna separata*] | 73.09 | ionotropic receptor, partial [*Helicoverpa armigera*] | 44.44 |
|  | c46397_g1 | GaurIR5 | 1139 | 963 \| 320 | 5' missing | No | 2 | gb\|QHB15331.1\| ionotropic receptor 76b [*Peridroma saucia*] | 68.04 | ionotropic receptor 76b, partial [*Helicoverpa assulta*] | 66.04 |
|  | c89666_g3 | GaurIR6 | 1061 | 915 \| 304 | 5' missing | No | 4 | gb\|AIG51915.1\| ionotropic receptor [*Helicoverpa armigera*] | 45.04 | ionotropic receptor, partial [*Helicoverpa armigera*] | 45.04 |
|  | c109228_g1 | GaurIR7 | 811 | 411 \| 136 | Yes | No | 1 | gb\|AIG51922.1\| ionotropic receptor [*Helicoverpa armigera*] | 83.80 | ionotropic receptor, partial [*Helicoverpa armigera*] | 83.80 |
|  | c164592_g1 | GaurIR8 | 690 | 687 \| 228 | 5', 3' missing | No | 2 | gb\|AJD81625.1\| ionotropic receptor 7d.3 [*Helicoverpa assulta*] | 45.87 | ionotropic receptor 7d.3, partial [*Helicoverpa assulta*] | 45.87 |
|  | c109059_g1 | GaurIR9 | 668 | 510 \| 169 | 3' missing | No | 3 | ref\|XP_035433110.1\| ionotropic receptor 75a-like [*Spodoptera frugiperda*] | 80.36 | ionotropic receptor 75a-like [*Spodoptera frugiperda*] | 80.36 |
|  | c104624_g1 | GaurIR10 | 632 | 630 \| 209 | 5', 3' missing | No | 3 | gb\|ALM24942.1\| ionotropic receptor 41a [*Athetis dissimilis*] | 76.59 | putative chemosensory ionotropic receptor IR41a [*Spodoptera littoralis*] | 74.38 |
|  | c89695_g1 | GaurIR11 | 608 | 435 \| 144 | Yes | No | 1 | gb\|ARB05668.1\| ionization receptor 75q2 [*Mythimna separata*] | 81.68 | ionization receptor 75q2 [*Mythimna separata*] | 81.68 |
|  | c105295_g1 | GaurIR12 | 554 | 396 \| 131 | 5' missing | No | 1 | gb\|AJD81631.1\| ionotropic receptor 41a [*Helicoverpa assulta*] | 78.18 | ionotropic receptor 41a, partial [*Helicoverpa assulta*] | 78.18 |
|  | c13648_g1 | GaurIR13 | 364 | 363 \| 120 | 5', 3' missing | No | 0 | gb\|AIG51921.1\| ionotropic receptor [*Helicoverpa armigera*] | 74.38 | ionotropic receptor, partial [*Helicoverpa armigera*] | 74.38 |
|  | c146743_g1 | GaurIR14 | 298 | 297 \| 98 | 5', 3' missing | No | 1 | gb\|AJD81640.1\| ionotropic receptor 76b [*Helicoverpa assulta*] | 83.56 | ionotropic receptor 76b, partial [*Helicoverpa assulta*] | 83.56 |
| gustatory receptors (GRs) | c134520_g1 | GaurGR1 | 1751 | 1368 \| 455 | Yes | No | 8 | ref\|XP_035448630.1\| gustatory receptor for sugar taste 43a-like [*Spodoptera frugiperda*] | 77.63 | gustatory receptor 4 [*Helicoverpa armigera*] | 76.97 |
|  | c104535_g1 | GaurGR2 | 1176 | 1017 \| 338 | 5' missing | No | 3 | Select seq ref\|XP_021199226.1\| glutamate receptor 1-like [*Helicoverpa armigera*] | 78.53 | ionotropic receptor, partial [*Helicoverpa armigera*] | 44.44 |
|  | c118902_g2 | GaurGR3 | 1149 | 636 \| 211 | 5' missing | No | 4 | gb\|AJD81596.1\| gustatory receptor 3 [*Helicoverpa assulta*] | 91.48 | gustatory receptor 3 [*Helicoverpa assulta*] | 91.48 |
|  | c118902_g1 | GaurGR4 | 847 | 816 \| 271 | 3' missing | No | 4 | gb\|AIG51909.1\| gustatory receptor [*Helicoverpa armigera*] | 83.09 | gustatory and odorant receptor 24 [*Helicoverpa armigera*] | 90.91 |
|  | c92521_g1 | GaurGR5 | 752 | 660 \| 219 | Yes | No | 2 | gb\|AIG51912.1\| gustatory receptor [*Helicoverpa armigera*] | 80.56 | gustatory receptor 6 [*Helicoverpa armigera*] | 80.56 |
|  | c80490_g1 | GaurGR6 | 734 | 366 \| 121 | Yes | No | 2 | gb\|AJD81595.1\| gustatory receptor 2 [*Helicoverpa assulta*] | 88.81 | *Helicoverpa armigera* gustatory receptor (Gr2) mRNA, complete cds | 78.19 |
|  | c115165_g1 | GaurGR7 | 685 | 642 \| 213 | 5' missing | Yes | 4 | tpg\|DAA06375.1\| TPA: gustatory receptor 11 [*Bombyx mori*] | 36.20 | TPA_inf: gustatory receptor 11 [*Bombyx mori*] | 36.20 |
|  | c165296_g1 | GaurGR8 | 666 | 654 \| 217 | 5' missing | No | 1 | ref\|XP_021188278.1\| gustatory receptor 68a isoform X2 [*Helicoverpa armigera*] | 60.83 | gustatory receptor 68a isoform X2 [*Helicoverpa armigera*] | 60.83 |
|  | c146708_g1 | GaurGR9 | 631 | 351 \| 116 | Yes | No | 2 | gb\|AJD81604.1\| gustatory receptor 11 [*Helicoverpa assulta*] | 43.16 | gustatory receptor 11, partial [*Helicoverpa assulta*] | 43.16 |
|  | c92124_g1 | GaurGR10 | 545 | 339 \| 112 | 5' missing | No | 2 | gb\|KOB66549.1\| Gustatory receptor 46 [*Operophtera brumata*] | 40.51 | gustatory receptor 46 [*Operophtera brumata*] | 40.51 |
|  | c63661_g1 | GaurGR11 | 508 | 483 \| 160 | 5' missing | No | 0 | ref\|XP_037871941.1\| gustatory receptor for sugar taste 64f-like [*Bombyx mori*] | 39.56 | gustatory receptor for sugar taste 64f-like [*Bombyx mori*] | 39.56 |
|  | c146365_g1 | GaurGR12 | 322 | 321 \| 106 | 5', 3' missing | No | 0 | gb\|AJD81604.1\| gustatory receptor 11 [*Helicoverpa assulta*] | 32.56 | gustatory receptor 11, partial [*Helicoverpa assulta*] | 32.56 |
| odorant degrading enzymes (ODEs) | c136660_g1 | GaurCXE1 | 3560 | 1653 \| 550 | Yes | Yes | 0 | gb\|AII21978.1\| odorant degrading enzyme CXE1 [*Sesamia inferens*] | 74.81 | odorant degrading enzyme CXE1 [*Sesamia inferens*] | 74.81 |
|  | c134735_g1 | GaurCXE2 | 2762 | 1626 \| 541 | Yes | Yes | 0 | gb\|AII21992.1\| odorant degrading enzyme CXE20 [*Sesamia inferens*] | 64.59 | odorant degrading enzyme CXE20 [*Sesamia inferens*] | 64.59 |
|  | c134134_g1 | GaurCXE3 | 2134 | 1653 \| 550 | Yes | Yes | 0 | gb\|AII21990.1\| odorant degrading enzyme CXE18 [*Sesamia inferens*] | 72.08 | odorant degrading enzyme CXE18 [*Sesamia inferens*] | 72.08 |
|  | c126824_g1 | GaurCXE4 | 1934 | 1785 \| 594 | 5' missing | No | 1 | gb\|AII21987.1\| odorant degrading enzyme CXE13 [*Sesamia inferens*] | 60.99 | odorant degrading enzyme CXE13 [*Sesamia inferens*] | 60.99 |
|  | c111179_g1 | GaurCXE5 | 1856 | 1761 \| 586 | Yes | No | 1 | gb\|AII21987.1\| odorant degrading enzyme CXE13 [*Sesamia inferens*] | 72.27 | odorant degrading enzyme CXE13 [*Sesamia inferens*] | 72.27 |
|  | c130873_g2 | GaurCXE6 | 1827 | 1128 \| 375 | 5' missing | No | 0 | gb\|AII21987.1\| odorant degrading enzyme CXE13 [*Sesamia inferens*] | 75.47 | odorant degrading enzyme CXE13 [*Sesamia inferens*] | 75.47 |
|  | c126077_g1 | GaurCXE7 | 1803 | 1611 \| 536 | 5' missing | No | 0 | gb\|AII21982.1\| odorant degrading enzyme CXE6 [*Sesamia inferens*] | 62.81 | odorant degrading enzyme CXE6, partial [*Sesamia inferens*] | 62.81 |
|  | c129797_g2 | GaurCXE8 | 1748 | 1527 \| 508 | 5' missing | No | 0 | gb\|AII21989.1\| odorant degrading enzyme CXE16 [*Sesamia inferens*] | 73.29 | odorant degrading enzyme CXE16, partial [*Sesamia inferens*] | 73.29 |
|  | c126369_g4 | GaurCXE9 | 686 | 684 \| 227 | 5', 3' missing | No | 0 | gb\|AII21980.1\| odorant degrading enzyme CXE3 [*Sesamia inferens*] | 54.39 | odorant degrading enzyme CXE3 [*Sesamia inferens*] | 54.39 |
|  | c94431_g1 | GaurCXE10 | 513 | 384 \| 127 | 5' missing | No | 0 | gb\|AII21984.1\| odorant degrading enzyme CXE10 [*Sesamia inferens*] | 64.80 | odorant degrading enzyme CXE10 [*Sesamia inferens*] | 64.80 |
|  | c15037_g1 | GaurCXE11 | 362 | 360 \| 119 | 5', 3' missing | No | 0 | gb\|AII21986.1\| odorant degrading enzyme CXE12 [*Sesamia inferens*] | 60.16 | odorant degrading enzyme CXE12, partial [*Sesamia inferens*] | 60.16 |
|  | c15037_g2 | GaurCXE12 | 357 | 357 \| 118 | 5', 3' missing | No | 0 | gb\|AII21982.1\| odorant degrading enzyme CXE6 [*Sesamia inferens*] | 42.50 | odorant degrading enzyme CXE6, partial [*Sesamia inferens*] | 42.50 |
| sensory neuron membrane protein genes (SNMPs) | c126636_g1 | GaurSNMP1 | 1702 | 1560 \| 519 | Yes | No | 2 | gb\|AGN52677.1\| sensory neuron membrane protein 2 [*Spodoptera exigua*] | 78.65 | sensory neuron membrane protein 2 [*Spodoptera exigua*] | 78.65 |
|  | c75543_g1 | GaurSNMP2 | 1624 | 1521 \| 506 | Yes | No | 2 | gb\|AKT26505.1\| sensory neuron membrane protein 3 [*Spodoptera exigua*] | 60.67 | sensory neuron membrane protein 2 [*Helicoverpa armigera*] | 60.00 |
