# Supplementary material for: Differential gene expression patterns between the head and thorax of Gynaephora aureata are associated with high-altitude adaptation
Source: Front Genet. 2023 Apr 18;14:1137618. doi: 10.3389/fgene.2023.1137618 (PMC10151491; doi:10.3389/fgene.2023.1137618)
Supplement: Supplementary file 1 [file DataSheet1.zip › Table S2.docx]

**Table S2. Occurrence of *Drosophila melanogaster* pigment pathway-associated genes in the head and thorax transcriptomes of *Gynaephora aureata* identified by a reciprocal BLAST hit (RBH) analysis. “⇒” represents a BLASTP hit (E < 1×10^-5^) between the *D. melanogaster* protein and a transcript in the head and thorax transcriptomes of *G. aureata*; “⇔” represents a reciprocal BLAST hit (BLASTP, E < 1×10^-5^) between the best BLASTP hit and NCBI database; “NA” represents no BLAST hit.**

| **Pigment pathway** | **Gene symbol** | **Gene name** | **Accession number** | **BLASTP best hit *G. aureata*** | **BLASTP *E* value** | **Reciprocal BLASTP best hit species** | **Reciprocal BLASTP *E* value** | **Identity** | **Reciprocal BLAST hit** |
| --- | --- | --- | --- | --- | --- | --- | --- | --- | --- |
| Rhodopsin | *EMC3* | *ER membrane protein complex subunit 3* | NP_609444 | c112587_g1 | 1.00E-138 | *Trichoplusia ni* | 5.00E-173 | 94.76% | ⇔ |
| Rhodopsin | *alpha-Man-IIa* | *alpha-Mannosidase class II a* | NP_001262413 | c129006_g1 | 0 | *Helicoverpa armigera* | 0 | 79.30% | ⇔ |
| Rhodopsin | *alpha-Man-IIb* | *alpha-Mannosidase class II b* | NP_650494 | c135897_g1 | 0 | *Trichoplusia ni* | 0 | 79.26% | ⇔ |
| Rhodopsin | *Cnx99A* | *Calnexin 99A* | NP_733286 | c135328_g1 | 0 | *Trichoplusia ni* | 0 | 77.60% | ⇔ |
| Rhodopsin | *Xport-A* | *exit protein of rhodopsin and TRP A* | NP_650846 | NA | NA | NA | NA | NA | NA |
| Rhodopsin | *Hexo1* | *Hexosaminidase 1* | NP_728975 | c111529_g1 | 0 | *Ostrinia furnacalis* | 0 | 79.78% | ⇔ |
| Rhodopsin | *ninaA* | *neither inactivation nor afterpotential A* | NP_476656 | c124135_g1 | 0 | *Manduca sexta* | 1.00E-137 | 82.67% | ⇔ |
| Rhodopsin | *ninaB* | *neither inactivation nor afterpotential B* | NP_650307 | c125145_g1 | 5.00E-147 | NA | NA | NA | ⇒ |
| Rhodopsin | *ninaD* | *neither inactivation nor afterpotential D* | NP_724087 | c133815_g1 | 8.00E-78 | NA | NA | NA | ⇒ |
| Rhodopsin | *ninaG* | *ninaG* | NP_001247047 | c131440_g1 | 1.00E-63 | *Trichoplusia ni* | 0 | 71.06% | ⇔ |
| Rhodopsin | *pinta* | *prolonged depolarization afterpotential (PDA) is not apparent* | NP_001287466 | c125492_g1 | 7.00E-32 | NA | NA | NA | NA |
| Rhodopsin | *santa-maria* | *scavenger receptor acting in neural tissue and majority of rhodopsin is absent* | NP_723277 | c133815_g1 | 9.00E-89 | NA | NA | NA | ⇒ |
| Rhodopsin | *Culd* | *CUB and LDLa domain* | NP_729364 | c119828_g2 | 6.00E-05 | NA | NA | NA | ⇒ |
| Rhodopsin | *fdl* | *fused lobes* | NP_001286350 | c132297_g1 | 3.00E-171 | *Spodoptera frugiperda* | 0 | 83.23% | ⇔ |
| Rhodopsin | *PAPLA1* | *Phosphatidic Acid Phospholipase A1* | NP_001285741 | c138749_g1 | 0 | NA | NA | NA | ⇒ |
| Rhodopsin | *roh* | *reduction of Rh1* | NP_726319 | NA | NA | NA | NA | NA | NA |
| Rhodopsin | *rdhB* | *retinol dehydrogenase B* | NP_001262831 | c126240_g1 | 2.00E-51 | NA | NA | NA | ⇒ |
| Ommochrome | *bw* | *brown* | NP_523824 | c134995_g1 | 8.00E-54 | *Papilio machaon* | 0 | 69.18% | ⇔ |
| Ommochrome | *cl* | *clot* | NP_001260098 | c149674_g1 | 8.00E-40 | *Drosophila busckii* | 2.00E-33 | 78.95% | ⇔ |
| Ommochrome | *Hn* | *Henna* | NP_523963 | c120137_g1 | 0 | *Trichoplusia ni* | 0 | 93.87% | ⇔ |
| Ommochrome | *cm* | *carmine* | NP_001259302 | c123095_g1 | 0 | *Limenitis arthemis astyanax* | 0 | 96.88% | ⇔ |
| Ommochrome | *car* | *carnation* | NP_728266 | c126873_g1 | 2.00E-148 | *Limenitis arthemis astyanax* | 0 | 81.02% | ⇔ |
| Ommochrome | *lt* | *light* | NP_001036415 | c130306_g1 | 0 | *Limenitis arthemis astyanax* | 0 | 79.77% | ⇔ |
| Ommochrome | *p* | *pink* | NP_001303455 | c132224_g1 | 2.00E-62 | *Limenitis arthemis astyanax* | 0 | 59.43% | ⇔ |
| Ommochrome | *rb* | *ruby* | NP_001259239 | c138739_g1 | 0 | *Limenitis arthemis astyanax* | 0 | 84.44% | ⇔ |
| Ommochrome | *or* | *orange* | NP_536793 | c126701_g1 | 3.00E-128 | *Limenitis arthemis astyanax* | 7.00E-135 | 96.86% | ⇔ |
| Ommochrome | *g* | *garnet* | NP_001259529 | c121884_g1 | 0 | *Limenitis arthemis astyanax* | 0 | 78.81% | ⇔ |
| Ommochrome | *cn* | *cinnabar* | NP_523651 | c111671_g1 | 9.00E-161 | *Limenitis arthemis astyanax* | 0 | 73.90% | ⇔ |
| Ommochrome | *kar* | *karmoisin* | NP_652025 | c130798_g3 | 6.00E-132 | *Heliconius melpomene cythera* | 1.00E-145 | 62.64% | ⇔ |
| Ommochrome | *st* | *scarlet* | NP_524108 | c132971_g1 | 0 | *Spodoptera frugiperda* | 0 | 83.15% | ⇔ |
| Ommochrome | *z* | *zeste* | NP_525051 | NA | NA | NA | NA | NA | NA |
| Ommochrome | *w* | *white* | NP_476787 | c128001_g1 | 0 | *Spodoptera litura* | 0 | 91.49% | ⇔ |
| Ommochrome | *HPS4* | *Hermansky-Pudlak Syndrome 4* | NP_725707 | c136975_g1 | 4.00E-150 | *Eumeta japonica* | 0 | 71.98% | ⇔ |
| Ommochrome | *po* | *pale ocelli* | NA | NA | NA | NA | NA | NA | NA |
| Ommochrome; Pteridine | *DhpD* | *Dihydropterin deaminase* | NP_649439 | c132835_g1 | 8.00E-124 | NA | NA | NA | ⇒ |
| Ommochrome; Pteridine | *mal* | *maroon-like* | NP_001285493 | c129431_g2 | 0 | NA | NA | NA | ⇒ |
| Ommochrome; Pteridine | *Pu* | *Punch* | NP_726037 | c135894_g2 | 5.00E-143 | NA | NA | NA | ⇒ |
| Ommochrome; Pteridine | *se* | *sepia* | NP_648235 | c56463_g1 | 6.00E-59 | NA | NA | NA | ⇒ |
| Ommochrome; Pteridine | *ca* | *claret* | NP_733309 | c134428_g2 | 2.00E-30 | NA | NA | NA | ⇒ |
| Ommochrome; Pteridine | *dor* | *deep orange* | NP_477286 | c134689_g1 | 0 | *Limenitis arthemis astyanax* | 0 | 70.96% | ⇔ |
| Ommochrome; Melanin | *Rab32* | *Rab32* | NP_724763 | c126401_g1 | 2.00E-122 | *Spodoptera frugiperda* | 7.00E-159 | 96.83% | ⇔ |
| Ommochrome; Heme | *cd* | *cardinal* | NP_651081 | c138628_g1 | 0 | *Limenitis arthemis astyanax* | 0 | 63.32% | ⇔ |
| Ommochrome; Heme | *v* | *vermilion* | NP_511113 | c116674_g1 | 0 | *Limenitis arthemis astyanax* | 0 | 90.00% | ⇔ |
| Pteridine | *pr* | *purple* | NP_724244 | c124385_g1 | 1.00E-62 | NA | NA | NA | ⇒ |
| Melanin | *Zir* | *Zizimin-related* | NP_608489 | c138567_g1 | 0 | NA | NA | NA | ⇒ |
| Melanin | *Cdc42* | *Cell division cycle 42* | NP_001245762 | c108686_g1 | 4.00E-123 | *Galleria mellonella* | 5.00E-134 | 98.43% | ⇔ |
| Melanin | *Cdk5* | *Cyclin-dependent kinase 5* | NP_477080 | c117450_g1 | 9.00E-148 | *Pectinophora gossypiella* | 8.00E-159 | 100.00% | ⇔ |
| Melanin | *Eph* | *Eph receptor tyrosine kinase* | NP_726590 | c132106_g1 | 0 | *Manduca sexta* | 0 | 90.82% | ⇔ |
| Melanin | *Flo2* | *Flotillin 2* | NP_001259553 | c133199_g2 | 0 | *Nymphalis io* | 0 | 94.80% | ⇔ |
| Melanin | *Rac1* | *Rac1* | NP_001261247 | c129363_g1 | 3.00E-133 | *Helicoverpa zea* | 3.00E-135 | 96.88% | ⇔ |
| Melanin | *Rac2* | *Rac2* | NP_001261517 | c129363_g1 | 2.00E-129 | NA | NA | NA | ⇒ |
| Melanin | *aPKC* | *atypical protein kinase C* | NP_524892 | c132766_g1 | 0 | *Trichoplusia ni* | 0 | 94.23% | ⇔ |
| Melanin | *bsk* | *basket* | NP_723541 | c125179_g1 | 1.00E-116 | NA | NA | NA | ⇒ |
| Melanin | *dl* | *dorsal* | NP_724052 | c137291_g1 | 6.00E-108 | *Helicoverpa armigera* | 0 | 55.06% | ⇔ |
| Melanin | *DCTN1-p150* | *Dynactin 1, p150 subunit* | NP_524061 | c138609_g1 | 0 | *Spodoptera frugiperda* | 0 | 86.73% | ⇔ |
| Melanin | *Dhc64C* | *Dynein heavy chain 64C* | NP_001261430 | c138730_g1 | 0 | *Helicoverpa zea* | 0 | 97.68% | ⇔ |
| Melanin | *e* | *ebony* | NP_524431 | c135749_g1 | 0 | *Chilo suppressalis* | 0 | 74.51% | ⇔ |
| Melanin | *egr* | *eiger* | NP_724878 | c133170_g1 | 9.00E-07 | *Helicoverpa zea* | 0 | 77.58% | ⇔ |
| Melanin | *grim* | *grim* | NP_524137 | NA | NA | NA | NA | NA | NA |
| Melanin | *Gr28b* | *Gustatory receptor 28b* | NP_647614 | c134520_g1 | 4.00E-09 | *Spodoptera frugiperda* | 0 | 78.62% | ⇔ |
| Melanin | *hep* | *hemipterous* | NP_001285182 | c131582_g1 | 7.00E-147 | NA | NA | NA | ⇒ |
| Melanin | *Nrg* | *Neuroglian* | NP_001162705 | c139743_g1 | 0 | *Spodoptera frugiperda* | 0 | 92.81% | ⇔ |
| Melanin | *PPO2* | *Prophenoloxidase 2* | NP_610443 | c139092_g1 | 0 | NA | NA | NA | ⇒ |
| Melanin | *Rho1* | *Rho1* | NP_599135 | c80185_g1 | 1.00E-140 | *Lucilia cuprina* | 7.00E-140 | 100.00% | ⇔ |
| Melanin | *Sp7* | *Serine protease 7* | NP_649734 | c119603_g1 | 2.00E-87 | *Spodoptera litura* | 0 | 66.75% | ⇔ |
| Melanin | *Spn27A* | *Serpin 27A* | NP_001260143 | c129007_g1 | 5.00E-87 | *Phthorimaea operculella* | 0 | 68.54% | ⇔ |
| Melanin | *y* | *yellow* | NP_476792 | c127028_g1 | 0 | *Spodoptera litura* | 0 | 74.80% | ⇔ |
| Melanin | *yellow-f* | *yellow-f* | NP_001262517 | c129696_g2 | 9.00E-97 | *Manduca sexta* | 0 | 75.57% | ⇔ |
| Melanin | *yellow-f2* | *yellow-f2* | NP_650247 | c117613_g1 | 8.00E-105 | *Helicoverpa armigera* | 0 | 65.49% | ⇔ |
| Melanin | *PPO3* | *Prophenoloxidase 3* | NP_524760 | c139092_g1 | 0 | NA | NA | NA | ⇒ |
| Melanin | *dia* | *diaphanous* | NP_001260640 | c138160_g1 | 2.00E-30 | NA | NA | NA | ⇒ |
| Melanin | *Als2* | *Amyotrophic lateral sclerosis 2* | NP_649347 | c139113_g1 | 1.00E-74 | NA | NA | NA | ⇒ |
| Melanin | *Cdk5alpha* | *Cdk5 activator-like protein* | NP_001260320 | c136518_g1 | 2.00E-93 | *Amyelois transitella* | 0 | 91.20% | ⇔ |
| Melanin | *Dronc* | *Death regulator Nedd2-like caspase* | NP_524017 | c131608_g1 | 2.00E-39 | *Lymantria dispar* | 0 | 78.08% | ⇔ |
| Melanin | *Dark* | *Death-associated APAF1-related killer* | NP_725638 | c138476_g1 | 6.00E-18 | *Trichoplusia ni* | 0 | 67.94% | ⇔ |
| Melanin | *DCX-EMAP* | *Doublecortin-domain-containing echinoderm-microtubule-associated protein* | NP_001261850 | c135983_g1 | 0 | NA | NA | NA | ⇒ |
| Melanin | *Eb1* | *Eb1* | NP_724495 | c121098_g1 | 3.00E-124 | NA | NA | NA | ⇒ |
| Melanin | *Ephrin* | *Ephrin* | NP_726584 | c142919_g1 | 3.00E-24 | *Trichoplusia ni* | 8.00E-43 | 90.83% | ⇔ |
| Melanin | *Itgbn* | *Integrin betanu subunit* | NP_523608 | c133644_g1 | 8.00E-175 | *Helicoverpa zea* | 0 | 79.04% | ⇔ |
| Melanin | *LanA* | *Laminin A* | NP_476617 | c139123_g1 | 0 | *Spodoptera litura* | 0 | 80.82% | ⇔ |
| Melanin | *lectin-37Da* | *lectin-37Da* | NP_001014489 | c62712_g1 | 7.00E-28 | *Lucilia cuprina* | 6.00E-64 | 54.43% | ⇔ |
| Melanin | *lectin-37Db* | *lectin-37Db* | NP_001014490 | c127829_g1 | 8.00E-18 | *Hyposmocoma kahamanoa* | 2.00E-130 | 67.64% | ⇔ |
| Melanin | *MP1* | *Melanization Protease 1* | NP_001138002 | c131677_g2 | 6.00E-86 | NA | NA | NA | ⇒ |
| Melanin | *Pten* | *Phosphatase and tensin homolog* | NP_477423 | c131522_g1 | 4.00E-97 | *Helicoverpa armigera* | 0 | 78.41% | ⇔ |
| Melanin | *poly* | *poly* | NP_001189215 | NA | NA | NA | NA | NA | ⇒ |
| Melanin | *PPO1* | *Prophenoloxidase 1* | NP_476812 | c139092_g1 | 0 | *Spodoptera exigua* | 0 | 80.44% | ⇔ |
| Melanin | *RhoGEF3* | *Rho guanine nucleotide exchange factor 3* | NP_001261202 | c132941_g1 | 3.00E-10 | *Phthorimaea operculella* | 0 | 82.67% | ⇔ |
| Melanin | *RhoL* | *Rho-like* | NP_001247003 | c121101_g1 | 2.00E-83 | *Bombyx mori* | 1.00E-125 | 92.90% | ⇔ |
| Melanin | *RhoBTB* | *Rho-related BTB domain containing* | NP_649216 | c136191_g1 | 0 | *Ostrinia furnacalis* | 0 | 91.19% | ⇔ |
| Melanin | *Shc* | *SHC-adaptor protein* | NP_524683 | c125854_g3 | 2.00E-32 | *Helicoverpa zea* | 0 | 90.07% | ⇔ |
| Melanin | *yellow-h* | *yellow-h* | NP_651912 | c136149_g1 | 3.00E-119 | *Heliconius melpomene* | 0 | 72.71% | ⇔ |
| Melanin | *Vav* | *Vav guanine nucleotide exchange factor* | NP_728235 | c138105_g1 | 1.00E-140 | *Papilio machaon* | 1.00E-97 | 95.39% | ⇔ |
| Melanin | *Exn* | *Ephexin* | NP_001097630 | c139559_g1 | 7.00E-161 | *Helicoverpa armigera* | 0 | 86.68% | ⇔ |
| Heme | *Alas* | *Aminolevulinate synthase* | NP_477281 | c126962_g1 | 0 | *Helicoverpa zea* | 0 | 90.53% | ⇔ |
| Heme | *Coprox* | *Coproporphyrinogen oxidase* | NP_001285697 | c138782_g1 | 0 | *Manduca sexta* | 0 | 83.25% | ⇔ |
| Heme | *FeCH* | *Ferrochelatase* | NP_524613 | c135990_g1 | 0 | *Papilio polytes* | 0 | 79.03% | ⇔ |
| Heme | *Ho* | *Heme oxygenase* | NP_524321 | c117179_g1 | 6.00E-34 | *Spodoptera frugiperda* | 0 | 81.56% | ⇔ |
| Heme | *Ppox* | *Protoporphyrinogen oxidase* | NP_651278 | c135609_g1 | 1.00E-143 | *Manduca sexta* | 0 | 79.57% | ⇔ |
| Heme | *Updo* | *Uroporphyrinogen decarboxylase* | NP_610501 | c124796_g3 | 0 | *Bombyx mandarina* | 0 | 87.15% | ⇔ |
| Heme | *CG3803* | *CG3803* | NP_611855 | c130476_g1 | 2.00E-154 | *Spodoptera litura* | 0 | 84.42% | ⇔ |
| Heme | *Cox10* | *Cytochrome c oxidase assembly factor 10* | NP_609382 | c129504_g1 | 2.00E-144 | NA | NA | NA | ⇒ |
| Heme | *Uros2* | *MIP06608p* | NA | NA | NA | NA | NA | NA | NA |
| Heme | *Pbgd* | *Porphobilinogen deaminase* | NA | NA | NA | NA | NA | NA | NA |
| Heme | *Pbgs* | *Porphobilinogen synthase* | NP_001261752 | c138740_g1 | 5.00E-137 | NA | NA | NA | ⇒ |
| Heme | *Dmel\CG34423* | *Uncharacterized protein, isoform B* | NP_001163263 | c171448_g1 | 2.00E-18 | *Lucilia sericata* | 1.00E-38 | 83.18% | ⇔ |
| Heme | *Uros1* | *Uroporphyrinogen III synthase 1* | NP_001259362 | c127016_g1 | 1.00E-29 | *Trichoplusia ni* | 2.00E-135 | 73.88% | ⇔ |
| Heme | *CG13551* | *CG13551* | NP_652302 | c171448_g1 | 3.00E-47 | *Lucilia sericata* | 1.00E-38 | 83.18% | ⇔ |
