# Supplementary material for: Differential gene expression patterns between the head and thorax of Gynaephora aureata are associated with high-altitude adaptation
Source: Front Genet. 2023 Apr 18;14:1137618. doi: 10.3389/fgene.2023.1137618 (PMC10151491; doi:10.3389/fgene.2023.1137618)
Supplement: Supplementary file 1 [file DataSheet1.zip › Table S3.docx]

**Table S3. Accession numbers of odorant-binding proteins (OBPs) and chemosensory proteins (CSPs) in other Lepidoptera used in motif pattern analyses.**

| **Gene symbol (abbreviation)** | **Species** | **Accession number** |
| --- | --- | --- |
| odorant-binding proteins (OBPs) | *Drosophila melanogaster* | NP_001263078.1 |
|  |  | NP_651707.1 |
|  |  | NP_611445.1 |
|  |  | NP_611709.1 |
|  |  | NP_524241.1 |
|  |  | NP_523505.1 |
|  |  | NP_001286186.1 |
|  |  | NP_611488.1 |
|  |  | NP_611448.2 |
|  |  | NP_611481.1 |
|  |  | NP_001286619.1 |
|  |  | NP_001097700.2 |
|  |  | NP_523421.2 |
|  |  | NP_524242.2 |
|  |  | NP_611442.1 |
|  |  | NP_731042.1 |
|  |  | NP_725973.1 |
|  |  | NP_001263077.1 |
|  |  | NP_524039.2 |
|  |  | NP_611447.1 |
|  |  | NP_725966.1 |
|  |  | NP_001286632.1 |
|  |  | NP_573350.1 |
|  |  | NP_727322.1 |
|  |  | NP_728338.2 |
|  |  | NP_651712.1 |
|  |  | NP_722746.2 |
|  |  | NP_725925.3 |
|  |  | NP_725926.1 |
|  | *Bombyx mori* | NP_001037498.1 |
|  |  | NP_001037496.1 |
|  |  | NP_001153664.1 |
|  |  | NP_001037494.1 |
|  |  | NP_001077095.1 |
|  |  | XP_012545845.1 |
|  |  | NP_001140185.1 |
|  |  | XP_004932340.1 |
|  |  | XP_021205117.2 |
|  |  | NP_001140190.1 |
|  |  | NP_001140188.1 |
|  |  | NP_001140191.1 |
|  |  | NP_001140189.1 |
|  |  | NP_001140187.1 |
|  |  | NP_001140186.1 |
|  |  | XP_004931990.1 |
|  |  | XP_037868365.1 |
|  |  | XP_004928236.2 |
|  | *Helicoverpa armigera* | XP_021192652.2 |
|  |  | XP_021187376.1 |
|  |  | XP_021200198.2 |
|  |  | XP_021196071.1 |
|  |  | XP_049700409.1 |
|  |  | XP_049700693.1 |
|  |  | XP_021193809.2 |
|  |  | XP_049700401.1 |
|  |  | XP_049700407.1 |
|  |  | XP_021188671.1 |
|  |  | XP_021187261.2 |
|  |  | XP_049700405.1 |
|  | *Trichoplusia ni* | XP_026744821.1 |
|  |  | XP_026744800.1 |
|  |  | XP_026739044.1 |
|  |  | XP_026737639.1 |
|  |  | XP_026731021.1 |
|  |  | XP_026731025.1 |
|  |  | XP_026730650.1 |
|  |  | XP_026739863.1 |
|  |  | XP_026736312.1 |
|  |  | XP_026731727.1 |
|  |  | XP_026731024.1 |
|  |  | XP_026739057.1 |
|  | *Spodoptera litura* | XP_022816701.1 |
|  |  | XP_022822379.1 |
|  |  | XP_022826721.1 |
|  |  | XP_022826780.1 |
|  |  | XP_022826781.1 |
|  |  | XP_022827633.1 |
|  |  | XP_022816506.1 |
|  |  | XP_022817831.1 |
|  |  | XP_022817865.1 |
|  |  | XP_022816230.1 |
|  | *Spodoptera frugiperda* | XP_035434733.1 |
|  |  | XP_035442487.1 |
|  |  | XP_050550081.1 |
|  |  | XP_050550076.1 |
|  |  | XP_050550074.1 |
|  |  | XP_050550072.1 |
|  |  | XP_035454669.1 |
|  |  | XP_035452971.2 |
|  |  | XP_035435754.2 |
|  |  | XP_035428835.1 |
|  |  | XP_035434730.2 |
|  | *Spodoptera exigua* | AKT26503.1 |
|  |  | AGH70100.1 |
|  |  | AGH70099.1 |
|  |  | AGH70098.1 |
|  |  | AGH70097.1 |
|  |  | CAC12831.1 |
|  |  | AKT26502.1 |
|  |  | AKT26500.1 |
|  |  | AKT26497.1 |
|  |  | AKT26504.1 |
|  |  | AKT26501.1 |
|  |  | AKT26499.1 |
|  |  | AKT26496.1 |
|  |  | AKT26495.1 |
|  |  | AGH70107.1 |
|  |  | AGH70106.1 |
|  |  | AGH70105.1 |
|  |  | AGH70104.1 |
|  |  | AGH70103.1 |
|  |  | AGH70102.1 |
|  |  | AGH70101.1 |
|  |  | CAC12832.1 |
|  |  | ACY78413.1 |
|  |  | AAU95537.1 |
|  |  | AAU95536.1 |
|  |  | AHI16728.1 |
|  |  | AGP03460.1 |
|  |  | AGP03459.1 |
|  |  | AGP03458.1 |
|  | *Lymantria dispar* | AAC47913.1 |
|  |  | AAC47914.1 |
| chemosensory proteins (CSPs) | *Drosophila melanogaster* | NP_995765.1 |
|  |  | NP_649035.2 |
|  |  | NP_995764.2 |
|  |  | NP_572395.2 |
|  |  | NP_001027174.1 |
|  |  | NP_610061.2 |
|  |  | NP_788004.1 |
|  |  | NP_995763.1 |
|  |  | NP_650970.2 |
|  |  | NP_001027438.1 |
|  |  | NP_732641.2 |
|  |  | NP_001027399.1 |
|  |  | NP_650280.1 |
|  |  | NP_001027150.1 |
|  |  | NP_995735.1 |
|  |  | NP_652269.3 |
|  |  | NP_995736.1 |
|  |  | NP_001014530.1 |
|  |  | NP_001027134.1 |
|  |  | NP_001014531.1 |
|  | *Bombyx mori* | ABH88194 |
|  |  | ABH88195 |
|  |  | ABH88196 |
|  |  | ABH88197 |
|  |  | ABH88198 |
|  |  | ABH88199 |
|  |  | ABH88200 |
|  |  | ABH88201 |
|  |  | ABH88202 |
|  |  | ABH88203 |
|  |  | ABH88204 |
|  |  | ABH88205 |
|  |  | ABH88206 |
|  |  | ABH88207 |
|  |  | ABH88208 |
|  |  | ABH88209 |
|  | *Helicoverpa armigera* | AAK53762 |
|  |  | AEX07265 |
|  |  | AEX07266 |
|  |  | AEX07269 |
|  |  | AEB54579 |
|  |  | AEX07267 |
|  |  | AEX07268 |
|  | *Spodoptera litura* | AAY26143.1 |
|  |  | ALJ30224.1 |
|  |  | ALJ30223.1 |
|  |  | ALJ30222.1 |
|  |  | ALJ30221.1 |
|  |  | ALJ30220.1 |
|  |  | ALJ30219.1 |
|  |  | ALJ30218.1 |
|  |  | ALJ30217.1 |
|  |  | ALJ30216.1 |
|  |  | ALJ30215.1 |
|  |  | ALJ30214.1 |
|  |  | ALJ30213.1 |
|  |  | ALJ30212.1 |
|  | *Spodoptera frugiperda* | QXT24547.1 |
|  |  | QXT24548.1 |
|  |  | QXT24549.1 |
|  |  | QXT24550.1 |
|  |  | QXT24551.1 |
|  |  | QXT24552.1 |
|  |  | UXY92042.1 |
|  | *Spodoptera exigua* | AKF42441.1 |
|  |  | AKT26494.1 |
|  |  | AKT26493.1 |
|  |  | AKT26492.1 |
|  |  | AKT26491.1 |
|  |  | AKT26490.1 |
|  |  | AKT26489.1 |
|  |  | AKT26488.1 |
|  |  | AKT26487.1 |
|  |  | AKT26486.1 |
|  |  | AKT26485.1 |
|  |  | AKT26484.1 |
|  |  | AKT26483.1 |
|  |  | AKT26482.1 |
|  |  | AKT26481.1 |
|  |  | AKF42444.1 |
|  |  | AKF42443.1 |
|  |  | AKF42440.1 |
|  |  | ABM67690.1 |
|  |  | AVC68637.1 |
|  |  | AVC68636.1 |
|  |  | AVC68635.1 |
