# Supplementary material for: Differential gene expression patterns between the head and thorax of Gynaephora aureata are associated with high-altitude adaptation
Source: Front Genet. 2023 Apr 18;14:1137618. doi: 10.3389/fgene.2023.1137618 (PMC10151491; doi:10.3389/fgene.2023.1137618)
Supplement: Supplementary file 1 [file DataSheet1.zip › Table S4.docx]

**Table S4. Evaluation of sequencing data for six samples in *Gynaephora aureata*. Q30 indicates that the base quality score is not less than 30.**

| **Sample** | **Sample no** | **Number of raw reads** | **Number of clean reads** | **Size of clean bases (Gb)** | **Clean reads rate (%)** | **Q30 (%)** |
| --- | --- | --- | --- | --- | --- | --- |
| Head | MQAZT1 | 50241724 | 45053582 | 6.75 | 89.67 | 96.21 |
|  | MQAZT2 | 52630764 | 47478452 | 7.12 | 90.21 | 96.39 |
|  | MQAZT3 | 49545954 | 44486760 | 6.67 | 89.79 | 96.29 |
| Thorax | MQAZX1 | 50837086 | 45606984 | 6.84 | 89.71 | 96.34 |
|  | MQAZX2 | 49472670 | 44377778 | 6.66 | 89.70 | 96.21 |
|  | MQAZX3 | 50362502 | 45345264 | 6.80 | 90.04 | 96.33 |
