# Supplementary material for: Differential gene expression patterns between the head and thorax of Gynaephora aureata are associated with high-altitude adaptation
Source: Front Genet. 2023 Apr 18;14:1137618. doi: 10.3389/fgene.2023.1137618 (PMC10151491; doi:10.3389/fgene.2023.1137618)
Supplement: Supplementary file 1 [file DataSheet1.zip › Table S5.docx]

**Table S5. Summary of the head and thorax transcriptome assemblies for *Gynaephora aureata*.**

| **Sequence length (bp)** | **Transcriptome number** | **Unigene number** |
| --- | --- | --- |
| <400 | 101741 (52.91%) | 80998 (61.08%) |
| 400-1000 | 54414 (28.3%) | 33369 (25.17%) |
| 1000-2000 | 21682 (11.27%) | 10925 (8.24%) |
| 2000-3000 | 7810 (4.06%) | 4004 (3.02%) |
| 3000-4000 | 3348 (1.74%) | 1637 (1.23%) |
| ≥4000 | 3307 (1.72%) | 1665 (1.26%) |
