# Supplementary material for: Differential gene expression patterns between the head and thorax of Gynaephora aureata are associated with high-altitude adaptation
Source: Front Genet. 2023 Apr 18;14:1137618. doi: 10.3389/fgene.2023.1137618 (PMC10151491; doi:10.3389/fgene.2023.1137618)
Supplement: Supplementary file 1 [file DataSheet1.zip › Table S6.docx]

**Table S6. Summary of annotations of the head and thorax transcriptomes of *Gynaephora aureata*.**

| **Annotated database** | **Number of annotated unigenes** | **Percentage (%)** |
| --- | --- | --- |
| Nr | 31872 | 24.04 |
| KOG | 8801 | 6.64 |
| Nt | 8077 | 6.09 |
| GO | 19385 | 14.62 |
| KEGG | 2907 | 2.19 |
| eggNOG | 12153 | 9.17 |
| BLASTX | 25006 | 18.86 |
| BLASTP | 16482 | 12.43 |
| Pfam | 16531 | 12.47 |
| Annotated in all databases | 5321 | 4.01 |
| Annotated in at least one database | 31883 | 24.05 |
| All | 40592 | 30.61 |
