# Supplementary material for: Differential gene expression patterns between the head and thorax of Gynaephora aureata are associated with high-altitude adaptation
Source: Front Genet. 2023 Apr 18;14:1137618. doi: 10.3389/fgene.2023.1137618 (PMC10151491; doi:10.3389/fgene.2023.1137618)
Supplement: Supplementary file 1 [file DataSheet1.zip › Table S8.docx]

**Table S8. Significantly differential expressed genes (sDEGs) between the head and thorax transcriptomes of Gynaephora aureata involved in carbohydrate metabolism, lipid metabolism, detoxication, cuticular protein, immune response and DNA repair.**

| **Gene style** | **Unigene ID** | **Log_2_ Fold Change (thorax/head)** | **Nr annotation** |
| --- | --- | --- | --- |
| Carbohydrate metabolism | c14354_g1 | 7.89 | Predicted: Glucose dehydrogenase [FAD, quinone]-like [*Bombyx mori*] |
|  | c119601_g1 | 7.67 | Predicted: Facilitated trehalose transporter Tret1 [*Bombyx mori*] |
|  | c115942_g2 | 6.48 | Beta-glucosidase precursor [*Danaus plexippus*] |
|  | c112064_g1 | 6.02 | Predicted: Glucose dehydrogenase [FAD, quinone]-like [*Bombyx mori*] |
|  | c126443_g1 | 5.62 | Predicted: Glucose dehydrogenase [FAD, quinone]-like [*Bombyx mori*] |
|  | c114910_g1 | 5.24 | Beta-glucosidase precursor [*Danaus plexippus*] |
|  | c136129_g1 | 4.93 | Predicted: Facilitated trehalose transporter Tret1-2 homolog [*Bombyx mori*] |
|  | c164160_g1 | 4.74 | Predicted: Facilitated trehalose transporter Tret1-2 homolog [*Bombyx mori*] |
|  | c56563_g1 | 4.73 | Predicted: Glucose dehydrogenase [FAD, quinone] [*Musca domestica*] |
|  | c126439_g1 | 4.53 | Alpha amylase [*Danaus plexippus*] |
|  | c30373_g1 | 4.43 | Beta-glucosidase precursor [*Spodoptera frugiperda*] |
|  | c55258_g1 | 4.13 | Predicted: UDP-glucose 4-epimerase-like [*Aplysia californica*] |
|  | c73986_g1 | 4.13 | Predicted: Facilitated trehalose transporter Tret1-like [*Plutella xylostella*] |
|  | c126834_g1 | 3.98 | Beta-glucosidase precursor [*Spodoptera frugiperda*] |
|  | c133335_g1 | 3.95 | Predicted: Facilitated trehalose transporter Tret1-like isoform X1 [*Bombyx mori*] |
|  | c93005_g3 | 3.64 | Glycogen phosphorylase [*Lucilia cuprina*] |
|  | c116575_g1 | 3.63 | Alpha amylase [*Danaus plexippus*] |
|  | c11892_g1 | 3.63 | Glycogen phosphorylase [*Lucilia cuprina*] |
|  | c130036_g1 | 3.30 | Beta-glucosidase precursor [*Danaus plexippus*] |
|  | c50420_g1 | 3.29 | Predicted: Facilitated trehalose transporter Tret1-2 homolog [*Bombyx mori*] |
|  | c121451_g1 | 3.27 | Alpha-amylase [*Helicoverpa armigera*] |
|  | c127253_g1 | 3.21 | Predicted: Facilitated trehalose transporter Tret1-like [*Bombyx mori*] |
|  | c122851_g1 | 3.07 | Predicted: Alpha-amylase 2-like [*Bombyx mori*] |
|  | c135481_g1 | 3.05 | Predicted: Glucose dehydrogenase [FAD, quinone]-like [*Bombyx mori*] |
|  | c119660_g1 | 3.02 | Uridine diphosphate glucosyltransferase precursor [*Bombyx mori*] |
|  | c105648_g1 | 2.98 | Beta-1,3-glucanase [*Heliconius* *melpomene*] |
|  | c114542_g2 | 2.89 | Predicted: Probable trehalose-phosphate phosphatase C [*Musca domestica*] |
|  | c132109_g1 | 2.81 | Putative neutral alpha-glucosidase ab [*Danaus plexippus*] |
|  | c128180_g1 | 2.71 | Predicted: Facilitated trehalose transporter Tret1-like [*Bombyx mori*] |
|  | c120060_g2 | 2.63 | Predicted: Facilitated trehalose transporter Tret1-like [*Bombyx mori*] |
|  | c120060_g1 | 2.60 | Predicted: Facilitated trehalose transporter Tret1 [*Plutella xylostella*] |
|  | c128897_g1 | 2.37 | Predicted: Glucose dehydrogenase [FAD, quinone]-like *[Plutella xylostella*] |
|  | c131693_g1 | 2.25 | Predicted: Glucose dehydrogenase [FAD, quinone]-like [*Plutella xylostella*] |
|  | c121964_g1 | 2.16 | Predicted: Glucose dehydrogenase [FAD, quinone]-like isoform X1 [*Bombyx mori*] |
|  | c133590_g2 | 2.06 | Predicted: Facilitated trehalose transporter Tret1 [*Bombyx mori*] |
|  | c122613_g1 | 2.04 | Predicted: Fructose-bisphosphate aldolase isoform X1 [*Bombyx mori*] |
|  | c133546_g1 | 1.72 | Predicted: Facilitated trehalose transporter Tret1-like isoform X1 [*Bombyx mori*] |
|  | c132009_g1 | 1.58 | Alpha amylase [*Antheraea pernyi*] |
|  | c130345_g1 | 1.58 | Trehalose 6-phosphate synthase isoform I [*Helicoverpa armigera*] |
|  | c121839_g1 | 1.54 | Predicted: Putative alpha-L-fucosidase [*Bombyx mori*] |
|  | c139097_g1 | 1.54 | Predicted: Facilitated trehalose transporter Tret1 [*Plutella xylostella*] |
|  | c125896_g1 | 1.53 | Predicted: Facilitated trehalose transporter Tret1-like [*Bombyx mori*] |
|  | c138030_g1 | 1.48 | Predicted: Putative hexokinase HKDC1 [*Bombyx mori*] |
|  | c133057_g1 | 1.48 | UDP-N-acetylglucosamine pyrophosphorylase [*Spodoptera exigua*] |
|  | c136173_g1 | 1.48 | Predicted: UDP-glucose 4-epimerase [*Bombyx mori*] |
|  | c123640_g2 | 1.45 | Facilitated trehalose transporter Tret1 [*Danaus plexippus*] |
|  | c135388_g2 | 1.39 | Predicted: Alpha-1,3-mannosyl-glycoprotein 4-beta-N-acetylglucosaminyltransferase B-like [*Bombyx mori*] |
|  | c131362_g1 | 1.36 | Beta-galactosidase [*Bombyx mori*] |
|  | c131453_g1 | 1.36 | Predicted: GDP-fucose transporter 1 [*Bombyx mori*] |
|  | c133351_g1 | 1.36 | Predicted: Beta-N-acetylglucosaminidase 1 isoform X1 [*Bombyx mori*] |
|  | c136022_g1 | 1.34 | Predicted: Glucose dehydrogenase [FAD, quinone]-like [*Bombyx mori*] |
|  | c128946_g1 | 1.33 | Glucose-1-phosphatase [*Danaus plexippus*] |
|  | c132752_g1 | 1.28 | Predicted: Glucosylceramidase-like [*Bombyx mori*] |
|  | c130032_g1 | 1.27 | Beta-N-acetylglucoasminidase 1 [*Spodoptera frugiperda*] |
|  | c138959_g6 | 1.27 | Predicted: Lysosomal alpha-mannosidase-like, partial [*Bombyx mori*] |
|  | c138762_g1 | 1.26 | Trehalase-like protein [*Heliconius erato*] |
|  | c124559_g1 | 1.25 | Predicted: Solute carrier family 2, facilitated glucose transporter member 6-like [*Bombyx mori*] |
|  | c138959_g5 | 1.24 | Predicted: Lysosomal alpha-mannosidase-like, partial [*Bombyx mori*] |
|  | c136055_g1 | 1.24 | Predicted: Mannosyl-oligosaccharide alpha-1,2-mannosidase isoform A isoform X2 [*Bombyx mori*] |
|  | c129787_g1 | 1.22 | UDP-glucose pyrophosphorylase [*Spodoptera exigua*] |
|  | c128656_g6 | 1.20 | Predicted: Facilitated trehalose transporter Tret1-like [*Bombyx mori*] |
|  | c125614_g1 | 1.20 | Predicted: Probable galactose-1-phosphate uridylyltransferase [*Plutella xylostella*] |
|  | c124942_g2 | 1.15 | Predicted: Carbohydrate-responsive element-binding protein isoform X4 [*Bombyx* *mori*] |
|  | c127965_g1 | 1.08 | Predicted: Facilitated trehalose transporter Tret1-like [*Bombyx* *mori*] |
|  | c130589_g2 | 1.04 | Predicted: Facilitated trehalose transporter Tret1-2 homolog isoform X1 [*Plutella* *xylostella*] |
|  | c138645_g1 | -1.05 | UDP-glycosyltransferase UGT33F2 [*Helicoverpa armigera*] |
|  | c133825_g3 | -1.07 | Predicted: Facilitated trehalose transporter Tret1-2 homolog [*Plutella xylostella*] |
|  | c134951_g1 | -1.25 | Predicted: Solute carrier family 2, facilitated glucose transporter member 8-like [*Bombyx mori*] |
|  | c126864_g1 | -1.48 | Predicted: Facilitated trehalose transporter Tret1-like isoform X3 [*Plutella xylostella*] |
|  | c129083_g3 | -1.48 | Predicted: Fructose 1,6-bisphosphate aldolase isoform X1 [*Bombyx mori*] |
|  | c132165_g2 | -1.61 | Predicted: Facilitated trehalose transporter Tret1-2 homolog [*Bombyx mori*] |
|  | c139425_g1 | -1.69 | UDP-glycosyltransferase UGT46A3 [*Helicoverpa armigera*] |
|  | c126190_g4 | -1.71 | Predicted: Facilitated trehalose transporter Tret1-like isoform X1 [*Bombyx mori*] |
|  | c88338_g2 | -2.51 | Predicted: UDP-glucose 6-dehydrogenase [*Musca domestica*] |
|  | c136346_g1 | -2.85 | Predicted: Glucose dehydrogenase [FAD, quinone]-like [*Plutella xylostella*] |
|  | c159384_g1 | -3.50 | Predicted: Glucose dehydrogenase [FAD, quinone]-like [*Plutella xylostella*] |
|  | c119601_g2 | -3.72 | Predicted: Facilitated trehalose transporter Tret1 [*Bombyx mori*] |
|  | c134019_g1 | -4.87 | Predicted: Glucose dehydrogenase [FAD, quinone]-like [*Bombyx mori*] |
|  | c127040_g2 | -6.11 | Predicted: Low quality protein: facilitated trehalose transporter Tret1-like [*Bombyx mori*] |
| Lipid metabolism | c1042_g1 | 7.99 | Fatty acid binding protein *[Spodoptera litura]* |
|  | c102244_g1 | 6.57 | Predicted: Pancreatic triacylglycerol lipase-like [*Bombyx mori*] |
|  | c106161_g1 | 6.40 | Predicted: Lipase 1-like [*Plutella xylostella*] |
|  | c91534_g1 | 6.39 | Predicted: Pancreatic lipase-related protein 2 [*Bombyx mori*] |
|  | c87071_g1 | 5.52 | Insect intestinal lipase 6 [*Mamestra configurata*] |
|  | c130486_g6 | 5.51 | Predicted: Elongation of very long chain fatty acids protein AAEL008004-like [*Bombyx mori*] |
|  | c127540_g2 | 5.38 | Predicted: Putative fatty acyl-CoA reductase CG5065 [*Bombyx mori*] |
|  | c127540_g1 | 5.18 | Predicted: Fatty acyl-CoA reductase 1-like [*Plutella xylostella*] |
|  | c94768_g1 | 4.47 | Neutral lipase [*Helicoverpa armigera*] |
|  | c13778_g1 | 4.28 | Predicted: Lipase-1 isoform X1 [*Bombyx mori*] |
|  | c116845_g1 | 4.28 | Predicted: Putative fatty acyl-CoA reductase CG5065 [*Bombyx mori*] |
|  | c49280_g1 | 4.09 | Fatty acid synthase 1, partial [*Helicoverpa assulta*] |
|  | c133843_g1 | 3.97 | Predicted: Elongation of very long chain fatty acids protein AAEL008004-like [*Bombyx mori*] |
|  | c138650_g1 | 3.78 | Predicted: Fatty acid synthase-like [*Bombyx mori*] |
|  | c105837_g1 | 3.63 | Insect intestinal lipase 6 [*Mamestra configurata*] |
|  | c111061_g1 | 3.63 | Predicted: Elongation of very long chain fatty acids protein 7-like isoform X1 [*Bombyx mori*] |
|  | c139651_g3 | 3.63 | Predicted: Fatty acid synthase-like [*Bombyx mori*] |
|  | c113362_g1 | 3.62 | Predicted: Putative fatty acyl-CoA reductase CG5065 [*Bombyx mori*] |
|  | c126397_g2 | 3.59 | Pancreatic lipase 1 [*Mamestra configurata*] |
|  | c139083_g1 | 3.58 | Predicted: Fatty acid synthase-like [*Bombyx mori*] |
|  | c121306_g1 | 3.58 | Acidic lipase [*Helicoverpa armigera*] |
|  | c93035_g1 | 3.52 | Neutral lipase [*Helicoverpa armigera*] |
|  | c124429_g1 | 3.50 | Predicted: Fatty acyl-CoA reductase 1-like [*Bombyx mori*] |
|  | c2368_g1 | 3.49 | Neutral lipase [*Helicoverpa armigera*] |
|  | c124500_g1 | 3.44 | Predicted: Elongation of very long chain fatty acids protein AAEL008004 isoform X1 [*Tribolium castaneum*] |
|  | c20348_g1 | 3.42 | Fatty-acyl-coa desaturase [*Operophtera brumata*] |
|  | c118257_g1 | 3.41 | Fatty acid-binding protein 2 [*Helicoverpa armigera*] |
|  | c138650_g2 | 3.35 | Predicted: Fatty acid synthase-like [*Bombyx mori*] |
|  | c126397_g1 | 3.34 | Predicted: Pancreatic triacylglycerol lipase-like [*Bombyx mori*] |
|  | c121886_g1 | 3.32 | Acidic lipase [*Helicoverpa armigera*] |
|  | c135748_g3 | 3.28 | Predicted: Fatty acyl-CoA reductase 1-like [*Bombyx mori*] |
|  | c130486_g7 | 3.26 | Predicted: Elongation of very long chain fatty acids protein AAEL008004-like [*Bombyx mori*] |
|  | c125673_g1 | 3.25 | Predicted: Pancreatic triacylglycerol lipase-like [*Plutella xylostella*] |
|  | c116285_g2 | 3.21 | Fatty acyl-coa reductase 2, partial [*Helicoverpa armigera*] |
|  | c135096_g1 | 3.21 | Predicted: Fatty acyl-CoA reductase 1-like [*Bombyx mori*] |
|  | c117667_g1 | 3.16 | Predicted: Elongation of very long chain fatty acids protein 7-like [*Plutella xylostella*] |
|  | c123026_g1 | 3.09 | Acidic lipase [*Helicoverpa armigera*] |
|  | c26318_g1 | 3.07 | Neutral lipase [*Helicoverpa armigera*] |
|  | c134258_g1 | 3.07 | Predicted: Cholesterol 25-hydroxylase-like [*Bombyx mori*] |
|  | c129831_g3 | 3.07 | Predicted: Sphingomyelin phosphodiesterase 1-like [*Bombyx mori*] |
|  | c39530_g1 | 3.00 | Predicted: Probable fatty acid-binding Protein [*Musca domestica*] |
|  | c126943_g1 | 2.72 | Predicted: Pancreatic triacylglycerol lipase-like [*Bombyx mori*] |
|  | c130215_g1 | 2.70 | Predicted: Fatty acid synthase-like [*Plutella xylostella*] |
|  | c110780_g1 | 2.48 | RecName: Full=Fatty acid-binding protein 1; Short=FABP 1 |
|  | c135205_g1 | 2.40 | Predicted: Putative fatty acyl-CoA reductase CG5065 [*Bombyx mori*] |
|  | c130881_g2 | 2.38 | Predicted: Elongation of very long chain fatty acids protein 4-like [*Plutella xylostella*] |
|  | c118797_g1 | 2.34 | Predicted: Elongation of very long chain fatty acids protein AAEL008004-like [*Plutella xylostella*] |
|  | c134114_g2 | 2.25 | Fatty alcohol acetyltransferase [*Agrotis segetum*] |
|  | c130881_g1 | 2.19 | Predicted: Elongation of very long chain fatty acids protein 4 [*Bombyx mori*] |
|  | c64911_g1 | 2.13 | Predicted: Fatty acyl-CoA reductase 2-like [*Bombyx mori*] |
|  | c114761_g1 | 2.13 | Predicted: Elongation of very long chain fatty acids protein 7-like [*Bombyx mori*] |
|  | c128613_g1 | 2.06 | Fatty alcohol acetyltransferase [*Agrotis segetum*] |
|  | c130546_g1 | 1.92 | Predicted: Lipase 1-like [*Bombyx mori*] |
|  | c131044_g1 | 1.89 | Predicted: Fatty-acid amide hydrolase 2-A-like isoform X1 [*Bombyx mori*] |
|  | c137124_g2 | 1.89 | Predicted: Lipase 3-like [*Plutella xylostella*] |
|  | c112388_g1 | 1.81 | Triacylglycerol lipase [*Danaus plexippus*] |
|  | c124309_g1 | 1.77 | Predicted: Sphingomyelin phosphodiesterase isoform X1 [*Bombyx mori*] |
|  | c127966_g1 | 1.76 | Pancreatic lipase 2 [*Mamestra configurata*] |
|  | c131719_g1 | 1.62 | Predicted: Pancreatic lipase-related protein 2-like [*Bombyx mori*] |
|  | c117311_g1 | 1.62 | Fatty alcohol acetyltransferase [*Agrotis segetum*] |
|  | c77609_g1 | 1.60 | Predicted: Elongation of very long chain fatty acids protein AAEL008004-like [*Bombyx mori*] |
|  | c131684_g2 | 1.59 | Predicted: Fatty-acid amide hydrolase 2 isoform X1 [*Bombyx mori*] |
|  | c136203_g2 | 1.47 | Predicted: Pancreatic lipase-related protein 2-like [*Bombyx mori*] |
|  | c130185_g1 | 1.45 | Predicted: Fatty acid 2-hydroxylase-like [*Plutella xylostella*] |
|  | c98556_g1 | 1.42 | Fatty-acyl coa reductase 6, partial [*Agrotis ipsilon*] |
|  | c131852_g1 | 1.36 | Neutral lipase [*Danaus plexippus*] |
|  | c52619_g1 | 1.32 | Predicted: Fatty acid-binding protein-like [*Bombyx mori*] |
|  | c121566_g1 | 1.29 | Predicted: Pancreatic lipase-related protein 2-like isoform X2 [*Bombyx mori*] |
|  | c128964_g1 | 1.28 | Fatty alcohol acetyltransferase [*Agrotis segetum*] |
|  | c135634_g1 | 1.27 | Predicted: Putative fatty acyl-CoA reductase CG5065 [*Plutella xylostella*] |
|  | c129093_g1 | 1.27 | Neutral lipase [*Helicoverpa armigera*] |
|  | c138747_g2 | 1.24 | Fatty acid synthase 1, partial [*Helicoverpa armigera*] |
|  | c138747_g1 | 1.20 | Fatty acid synthase [*Agrotis ipsilon*] |
|  | c132467_g1 | 1.06 | Predicted: Group XV phospholipase A2-like [*Bombyx* *mori*] |
|  | c104209_g1 | 1.06 | Fatty alcohol acetyltransferase [*Agrotis* *segetum*] |
|  | c128706_g1 | 1.04 | Fatty acid transport protein 4 [*Sesamia* *inferens*] |
|  | c124421_g1 | -1.08 | Predicted: Acid sphingomyelinase-like phosphodiesterase 3a [*Plutella xylostella*] |
|  | c135697_g1 | -1.11 | Predicted: Putative fatty acyl-CoA reductase CG5065 [*Bombyx mori*] |
|  | c132600_g2 | -1.32 | Fatty acyl-coa reductase 8, partial [*Helicoverpa assulta*] |
|  | c125380_g1 | -1.35 | Neutral lipase [*Danaus plexippus*] |
|  | c132331_g1 | -1.37 | Predicted: Lipase member H-B-like [*Bombyx mori*] |
|  | c99817_g1 | -1.45 | Predicted: Pancreatic lipase-related protein 2-like [*Bombyx mori*] |
|  | c138111_g1 | -1.69 | Predicted: Liprin-beta-1 isoform X2 [*Bombyx mori*] |
|  | c129244_g2 | -1.71 | Predicted: Elongation of very long chain fatty acids protein AAEL008004 isoform X1 [*Bombyx mori*] |
|  | c128467_g1 | -1.77 | Fatty acyl-coa reductase 7, partial [*Helicoverpa assulta*] |
|  | c130252_g1 | -2.33 | Fatty acyl-coa reductase 5, partial [*Helicoverpa assulta*] |
|  | c129019_g1 | -3.21 | Predicted: Elongation of very long chain fatty acids protein 7 [*Bombyx mori*] |
|  | c38154_g1 | -3.31 | Predicted: Putative fatty acyl-CoA reductase CG5065 [*Bombyx mori*] |
|  | c112489_g1 | -5.75 | RecName: Full=Adipokinetic prohormone; Contains: RecName: Full=Adipokinetic hormone; Short=AKH; Flags: Precursor [*Manduca sexta*] |
| Detoxication | c88376_g1 | 6.78 | Cytochrome P450 CYP339A1 [*Bombyx mori*] |
|  | c96999_g1 | 5.40 | Cytochrome P450 CYP6B43 [*Helicoverpa armigera*] |
|  | c114532_g1 | 4.43 | Glutathione S-transferases 1-1 [*Lucilia sericata*] |
|  | c109601_g2 | 3.86 | Microsomal glutathione S-transferase 1-5 [*Spodoptera litura*] |
|  | c117684_g1 | 3.77 | Cytochrome P450 CYP4G26 [*Helicoverpa armigera*] |
|  | c120831_g1 | 3.69 | Carboxylesterase-like protein [*Helicoverpa armigera*] |
|  | c125640_g1 | 3.48 | Predicted: Low quality protein: venom carboxylesterase-6-like [*Bombyx mori*] |
|  | c120609_g1 | 3.14 | Glutathione S-transferase epsilon 7, partial [*Spodoptera litura*] |
|  | c116808_g1 | 3.05 | Cytochrome P450 CYP6AB10 [*Helicoverpa armigera*] |
|  | c98934_g1 | 3.01 | Carboxylesterase, partial [*Agrotis ipsilon*] |
|  | c130166_g2 | 3.01 | Glutathione S-transferase sigma 4 [*Spodoptera litura*] |
|  | c125818_g1 | 2.88 | Cytochrome P450 CYP6AE51, partial [*Lymantria dispar*] |
|  | c122946_g1 | 2.82 | Cytochrome P450 CYP4G9 [*Helicoverpa armigera*] |
|  | c108238_g1 | 2.80 | Glutathione S-transferase unclassified 1 [*Bombyx mori*] |
|  | c122468_g1 | 2.47 | Carboxylesterase [*Helicoverpa armigera*] |
|  | c114793_g1 | 2.28 | Cytochrome P450 CYP4M10v2 [*Helicoverpa armigera*] |
|  | c122289_g1 | 2.24 | Carboxylesterase CXE26 [*Spodoptera littoralis*] |
|  | c132112_g5 | 2.19 | Cytochrome P450 CYP6AB10 [*Helicoverpa armigera*] |
|  | c133946_g2 | 2.12 | Cytochrome P450 CYP6AB32, partial [*Lymantria dispar*] |
|  | c132484_g1 | 2.09 | Venom carboxylesterase-6-like precursor [*Bombyx mori*] |
|  | c125967_g1 | 2.04 | Glutathione S-transferase epsilon 11 [*Spodoptera litura*] |
|  | c77793_g1 | 2.03 | Cytochrome P450 CYP9A3 [*Helicoverpa armigera*] |
|  | c134723_g3 | 2.03 | Cytochrome P450 CYP321A7 [*Spodoptera frugiperda*] |
|  | c129028_g1 | 2.00 | Cytochrome P450 CYP6AB9 [*Helicoverpa armigera*] |
|  | c124042_g1 | 1.96 | Microsomal glutathione S-transferase 1-1 [*Spodoptera litura*] |
|  | c130677_g1 | 1.93 | Cytochrome P450 CYP340G1 [*Helicoverpa armigera*] |
|  | c112317_g1 | 1.90 | Glutathione S-transferase epsilon 2 [*Spodoptera litura*] |
|  | c132112_g4 | 1.81 | Cytochrome P450 CYP6AB36, partial [*Lymantria dispar*] |
|  | c111518_g1 | 1.76 | Glutathione S-transferase epsilon 15 [*Spodoptera litura*] |
|  | c128894_g1 | 1.76 | Cytochrome P450 CYP341D1 [*Helicoverpa armigera*] |
|  | c128534_g2 | 1.68 | Cytochrome P450, partial [*Helicoverpa armigera*] |
|  | c131294_g1 | 1.68 | Cytochrome P450 CYP6AN15v1 [*Lymantria dispar*] |
|  | c130472_g1 | 1.63 | Cytochrome P450 337B3v4 [*Helicoverpa armigera*] |
|  | c130018_g1 | 1.58 | Cytochrome P450 monooxygenase CYP4M6 [*Helicoverpa zea*] |
|  | c111329_g1 | 1.54 | Cytochrome P450 CYP6AB37 [*Lymantria dispar*] |
|  | c137456_g2 | 1.47 | Carboxylesterase [*Cnaphalocrocis medinalis*] |
|  | c130065_g2 | 1.45 | Cytochrome P450 CYP332A1 [*Helicoverpa armigera*] |
|  | c136273_g1 | 1.41 | Cytochrome P450 CYP304F1 [*Helicoverpa armigera*] |
|  | c122243_g1 | 1.38 | Cytochrome P450 *[Helicoverpa armigera*] |
|  | c122218_g1 | 1.37 | Cytochrome P450 CYP306A1 [*Helicoverpa armigera*] |
|  | c130781_g1 | 1.37 | Glutathione S-transferase omega 2 [*Spodoptera litura*] |
|  | c113871_g1 | 1.33 | Cytochrome P450 CYP4G75 [*Spodoptera frugiperda*] |
|  | c137447_g1 | 1.22 | Cytochrome P450 CYP4L5 [*Helicoverpa armigera*] |
|  | c127892_g4 | 1.18 | Carboxylesterase care-11 precursor [*Bombyx mori*] |
|  | c126841_g1 | 1.17 | Predicted: Glutathione S-transferase 1-like [*Plutella xylostella*] |
|  | c93497_g1 | 1.17 | Glutathione S-transferase sigma 5 [*Spodoptera litura*] |
|  | c124557_g1 | 1.13 | Cytochrome P450 CYP6AE51, partial [*Lymantria* *dispar*] |
|  | c130705_g1 | 1.08 | Glutathione S-transferase epsilon 5 [*Spodoptera* *litura*] |
|  | c132763_g1 | 1.07 | Cytochrome P450 CYP6AE51, partial [*Lymantria* *dispar*] |
|  | c138613_g1 | -1.03 | Carboxylesterase CXE30 [*Spodoptera littoralis*] |
|  | c131675_g1 | -1.32 | Cytochrome P450 CYP9AJ3 [*Helicoverpa armigera*] |
|  | c131721_g2 | -1.42 | Cytochrome P450 CYP4S12 [*Helicoverpa armigera*] |
|  | c137746_g1 | -3.10 | Cytochrome P450 CYP307A1 [*Helicoverpa armigera*] |
|  | c135681_g2 | -5.59 | Cytochrome P450, partial [*Bombyx mori*] |
| Cuticular protein | c122931_g1 | 9.16 | Putative cuticle protein CPH45 [*Antheraea yamamai*] |
|  | c97896_g1 | 8.75 | Putative cuticle protein CPH38 [*Danaus plexippus*] |
|  | c81147_g1 | 5.76 | Cuticular protein RR-2 motif 128 [*Danaus plexippus*] |
|  | c780_g1 | 2.89 | Larval cuticle protein LCP-17 precursor [*Bombyx mori*] |
|  | c60025_g1 | 2.40 | Cuticular protein RR-2 motif 130 precursor [*Bombyx mori*] |
|  | c104920_g1 | 1.76 | Larval cuticle protein LCP-30 precursor [*Bombyx mori*] |
|  | c102188_g1 | 1.56 | Predicted: Larval cuticle protein III/IV-like [*PlutelLa xylostelLa*] |
|  | c111370_g1 | 1.53 | Cuticular protein RR-1 motif 40 precursor [*Bombyx mori*] |
|  | c157921_g1 | 1.42 | Cuticular protein RR-1 motif 27 precursor [*Bombyx mori*] |
|  | c96248_g1 | 1.35 | RecName: Full=Flexible cuticle protein 12; Flags: Precursor [*Hyalophora cecropia*] |
|  | c113908_g1 | 1.22 | Predicted: Cuticular protein glycine-rich 21 isoform X1 [*Bombyx mori*] |
|  | c101471_g1 | 1.22 | Larval cuticle protein LCP-22 precursor [*Bombyx mori*] |
|  | c107269_g1 | 1.18 | Predicted: Larval cuticle protein 1-like [*Plutella xylostella*] |
|  | c138090_g5 | 1.13 | Predicted: Epidermal growth factor receptor kinase substrate 8-like isoform X3 [*Bombyx* *mori*] |
|  | c130575_g2 | -1.00 | Putative cuticle protein CPH41 [*Danaus plexippus*] |
|  | c101690_g1 | -1.13 | TPAPutative cuticle protein [*Danaus plexippus*] |
|  | c111882_g1 | -1.28 | Cuticular protein RR-1 motif 26 precursor [*Bombyx mori*] |
|  | c101508_g1 | -1.37 | Cuticular protein ppolcpr77 [*Papilio polytes*] |
|  | c120373_g1 | -1.39 | Cuticular protein RR-2 motif 63 [*Danaus plexippus*] |
|  | c131468_g1 | -1.48 | Predicted: Cuticular protein RR-2 motif 131 isoform X1 [*Bombyx mori*] |
|  | c135068_g1 | -1.65 | Cuticular protein RR-2 motif 79 precursor [*Bombyx mori*] |
|  | c136033_g1 | -1.65 | Cuticular protein ppolcpr55 [*Papilio polytes*] |
|  | c126227_g1 | -1.94 | Cuticle protein [*Helicoverpa armigera*] |
|  | c107849_g1 | -1.95 | Cuticular protein RR-2 motif 69 precursor [*Bombyx mori*] |
|  | c99658_g1 | -2.08 | Putative cuticular protein [*Papilio xuthus*] |
|  | c120135_g1 | -2.13 | Cuticular protein CPFL6Bb [*Papilio xuthus*] |
|  | c98551_g1 | -2.37 | Cuticular protein ppolcph2 [*Papilio polytes*] |
|  | c45215_g1 | -2.77 | Cuticular protein ppolcph8 [*Papilio polytes*] |
|  | c122478_g1 | -2.82 | Cuticular protein RR-1 motif 11 precursor [*Bombyx mori*] |
|  | c105094_g1 | -2.93 | Cuticular protein RR-2 motif 87 precursor [*Bombyx mori*] |
|  | c111704_g1 | -3.04 | Cuticular protein ppolcpr59a [*Papilio polytes*] |
|  | c121785_g1 | -3.05 | Predicted: Cuticle protein 8 [*Bombyx mori*] |
|  | c121338_g1 | -3.13 | Putative cuticular protein [*Papilio xuthus*] |
|  | c158136_g1 | -3.16 | Cuticular protein RR-1 motif 23 precursor [*Bombyx mori*] |
|  | c117294_g1 | -3.28 | Cuticular protein ppolcph30 [*Papilio polytes*] |
|  | c126270_g1 | -3.77 | Pupal cuticle protein [*Heliothis virescens*] |
|  | c122073_g2 | -3.78 | Cuticular protein RR-2 motif 72 [*Bombyx mori*] |
|  | c112872_g1 | -4.26 | Cuticular protein RR-1 motif 17 precursor [*Bombyx mori*] |
|  | c111599_g1 | -4.38 | Cuticular protein hypothetical 12 precursor [*Bombyx mori*] |
|  | c123398_g1 | -4.45 | Predicted: Pupal cuticle protein 27-like [*Bombyx mori*] |
|  | c122065_g1 | -4.57 | Predicted: Cuticular protein RR-1 motif 33 isoform X1 [*Bombyx mori*] |
|  | c110064_g1 | -4.57 | Cuticular protein hypothetical 8 precursor [*Bombyx mori*] |
|  | c110448_g1 | -4.61 | Cuticular protein RR-1 motif 7 precursor [*Bombyx mori*] |
|  | c127595_g1 | -4.68 | Cuticular protein RR-2 motif 65 precursor [*Bombyx mori*] |
|  | c131284_g1 | -4.75 | Cuticular protein RR-1 motif 15 precursor [*Bombyx mori*] |
|  | c121694_g1 | -4.81 | TPA: Putative cuticle protein [*Bombyx mori*] |
|  | c120145_g1 | -4.89 | Cuticular protein pxutcpr10 [*Papilio xuthus*] |
|  | c107539_g1 | -4.91 | pupal cuticle protein [*Heliothis virescens*] |
|  | c110077_g2 | -5.11 | Cuticular protein RR-2 motif 87 [*Danaus plexippus*] |
|  | c111599_g2 | -5.21 | Cuticular protein hypothetical 12 [*Danaus plexippus*] |
|  | c116341_g1 | -5.21 | Cuticular protein CPR71 [*Papilio xuthus*] |
|  | c75816_g1 | -5.26 | Cuticular protein RR-1 motif 32 precursor [*Bombyx mori*] |
|  | c107939_g1 | -5.46 | Putative cuticular protein [*Papilio xuthus*] |
|  | c119416_g1 | -5.82 | Cuticular protein hypothetical 17 precursor [*Bombyx mori*] |
|  | c110077_g1 | -5.93 | Cuticular protein RR-2 motif 87 precursor [*Bombyx mori*] |
|  | c115503_g1 | -6.18 | Cuticular protein hypothetical 9 precursor [*Bombyx mori*] |
|  | c107150_g1 | -6.49 | Putative cuticular protein [*Danaus plexippus*] |
|  | c117674_g1 | -6.85 | Cuticular protein hypothetical 15 precursor [*Bombyx mori*] |
|  | c120483_g1 | -6.99 | Cuticular protein RR-2 motif 87 [*Danaus plexippus*] |
|  | c98821_g1 | -7.05 | Predicted: Cuticle protein 1-like [*Plutella xylostella*] |
|  | c125453_g1 | -7.15 | Cuticular protein hypothetical 7 precursor [*Bombyx mori*] |
|  | c56275_g1 | -7.19 | TPAPutative cuticle protein [*Danaus plexippus*] |
|  | c120483_g2 | -7.41 | Cuticular protein RR-2 motif 87 [*Danaus plexippus*] |
|  | c122649_g1 | -7.87 | Putative cuticle protein [*Danaus plexippus*] |
|  | c116748_g1 | -8.87 | Cuticular protein RR-2 motif 63 [*Danaus plexippus*] |
|  | c117674_g2 | -9.20 | Cuticular protein hypothetical 15 precursor [*Bombyx mori*] |
| Immune response | c129400_g2 | 2.06 | Immulectin [*Ostrinia furnacalis*] |
|  | c131047_g5 | 1.09 | Immulectin III [*Manduca* *sexta*] |
|  | c134386_g1 | 1.07 | Immulectin [*Ostrinia* *furnacalis*] |
| DNA repair | c131211_g1 | 1.01 | DNA repair protein RAD51-like protein 3 [*Zootermopsis nevadensis*] |
|  | c130952_g2 | -5.92 | Predicted: DNA mismatch repair protein Msh3-like [*Bombyx mori*] |
