# Supplementary material for: Differential gene expression patterns between the head and thorax of Gynaephora aureata are associated with high-altitude adaptation
Source: Front Genet. 2023 Apr 18;14:1137618. doi: 10.3389/fgene.2023.1137618 (PMC10151491; doi:10.3389/fgene.2023.1137618)
Supplement: Supplementary file 1 [file DataSheet1.zip › Table S9.docx]

**Table S9. All significantly enriched Gene Ontology (GO) terms determined by a GO enrichment analysis of significantly differentially expressed genes (sDEGs) between the head and thorax transcriptomes of *Gynaephora aureata*.**

| **Level 1** | **GO ID** | **GO term** | ***P* value** | **FDR** |
| --- | --- | --- | --- | --- |
| Biological Process | GO:0031032 | Actomyosin structure organization | 2.00E-04 | 2.07E-02 |
|  | GO:0030534 | Adult behavior | 6.00E-04 | 4.18E-02 |
|  | GO:0032328 | Alanine transport | 3.00E-04 | 2.69E-02 |
|  | GO:0046164 | Alcohol catabolic process | 0.00E+00 | 4.10E-03 |
|  | GO:0006066 | Alcohol metabolic process | 0.00E+00 | 3.00E-04 |
|  | GO:1901606 | Alpha-amino acid catabolic process | 0.00E+00 | 5.30E-03 |
|  | GO:0043604 | Amide biosynthetic process | 3.00E-04 | 2.41E-02 |
|  | GO:0009310 | Amine catabolic process | 0.00E+00 | 1.00E-04 |
|  | GO:0006865 | Amino acid transport | 0.00E+00 | 2.10E-03 |
|  | GO:0006040 | Amino sugar metabolic process | 0.00E+00 | 0.00E+00 |
|  | GO:0006026 | Aminoglycan catabolic process | 0.00E+00 | 1.00E-04 |
|  | GO:0006022 | Aminoglycan metabolic process | 0.00E+00 | 0.00E+00 |
|  | GO:0009653 | Anatomical structure morphogenesis | 4.00E-04 | 3.13E-02 |
|  | GO:0006820 | Anion transport | 0.00E+00 | 4.00E-04 |
|  | GO:0002777 | Antimicrobial peptide biosynthetic process | 4.00E-04 | 3.01E-02 |
|  | GO:0002775 | Antimicrobial peptide production | 4.00E-04 | 3.01E-02 |
|  | GO:0007610 | Behavior | 0.00E+00 | 1.00E-04 |
|  | GO:0008218 | Bioluminescence | 1.00E-04 | 9.00E-03 |
|  | GO:0046395 | Carboxylic acid Catabolic process | 0.00E+00 | 0.00E+00 |
|  | GO:0019752 | Carboxylic acid metabolic process | 5.00E-04 | 4.15E-02 |
|  | GO:0046942 | Carboxylic acid transport | 2.00E-04 | 1.82E-02 |
|  | GO:0006812 | Cation transport | 1.00E-04 | 1.04E-02 |
|  | GO:0009063 | Cellular amino acid catabolic process | 0.00E+00 | 3.00E-04 |
|  | GO:0042402 | Cellular biogenic amine catabolic process | 0.00E+00 | 1.00E-04 |
|  | GO:0006576 | Cellular biogenic amine metabolic process | 7.00E-04 | 4.64E-02 |
|  | GO:0034754 | Cellular hormone metabolic process | 7.00E-04 | 4.64E-02 |
|  | GO:0044242 | Cellular lipid catabolic process | 0.00E+00 | 3.90E-03 |
|  | GO:0044255 | Cellular lipid metabolic process | 0.00E+00 | 0.00E+00 |
|  | GO:0006030 | Chitin metabolic process | 0.00E+00 | 0.00E+00 |
|  | GO:0003341 | Cilium movement | 1.00E-04 | 1.32E-02 |
|  | GO:0030574 | Collagen catabolic process | 0.00E+00 | 0.00E+00 |
|  | GO:0032963 | Collagen metabolic process | 0.00E+00 | 1.00E-04 |
|  | GO:0002534 | Cytokine production involved in inflammatory response | 7.00E-04 | 4.64E-02 |
|  | GO:0042742 | Defense response to bacterium | 0.00E+00 | 3.60E-03 |
|  | GO:0050830 | Defense response to Gram-positive bacterium | 0.00E+00 | 9.00E-04 |
|  | GO:0032502 | Developmental process | 0.00E+00 | 4.00E-04 |
|  | GO:0007586 | Digestion | 0.00E+00 | 0.00E+00 |
|  | GO:0097070 | Ductus arteriosus closure | 1.00E-04 | 1.23E-02 |
|  | GO:0006633 | Fatty acid biosynthetic process | 0.00E+00 | 5.00E-04 |
|  | GO:1901568 | Fatty acid derivative metabolic process | 0.00E+00 | 1.00E-04 |
|  | GO:0030497 | Fatty acid elongation | 0.00E+00 | 1.80E-03 |
|  | GO:0034626 | Fatty acid elongation, polyunsaturated Fatty acid | 1.00E-04 | 1.04E-02 |
|  | GO:0019367 | Fatty acid elongation, saturated Fatty acid | 0.00E+00 | 1.30E-03 |
|  | GO:0019368 | Fatty acid elongation, unsaturated Fatty acid | 0.00E+00 | 1.20E-03 |
|  | GO:0006631 | Fatty acid metabolic process | 0.00E+00 | 0.00E+00 |
|  | GO:0060361 | Flight | 1.00E-04 | 9.00E-03 |
|  | GO:0007629 | Flight behavior | 6.00E-04 | 4.64E-02 |
|  | GO:0019376 | Galactolipid catabolic process | 0.00E+00 | 2.90E-03 |
|  | GO:0019374 | Galactolipid metabolic process | 0.00E+00 | 6.00E-04 |
|  | GO:1901071 | Glucosamine-containing compound metabolic process | 0.00E+00 | 0.00E+00 |
|  | GO:0019377 | Glycolipid catabolic process | 7.00E-04 | 4.72E-02 |
|  | GO:0006027 | Glycosaminoglycan catabolic process | 0.00E+00 | 1.20E-03 |
|  | GO:0007186 | G-protein coupled receptor signaling pathway | 0.00E+00 | 1.60E-03 |
|  | GO:0042446 | Hormone biosynthetic process | 0.00E+00 | 4.10E-03 |
|  | GO:0042445 | Hormone metabolic process | 0.00E+00 | 2.00E-03 |
|  | GO:0006818 | Hydrogen transport | 4.00E-04 | 3.47E-02 |
|  | GO:0006690 | Icosanoid metabolic process | 0.00E+00 | 1.00E-04 |
|  | GO:0002433 | Immune response-regulating cell surface receptor signaling pathway involved in phagocytosis | 7.00E-04 | 4.64E-02 |
|  | GO:0046218 | Indolalkylamine catabolic process | 1.00E-04 | 1.04E-02 |
|  | GO:0006586 | Indolalkylamine metabolic process | 1.00E-04 | 1.23E-02 |
|  | GO:0042436 | Indole-containing compound catabolic process | 0.00E+00 | 6.00E-04 |
|  | GO:0042430 | Indole-containing compound metabolic process | 1.00E-04 | 6.40E-03 |
|  | GO:0015698 | Inorganic anion transport | 7.00E-04 | 4.64E-02 |
|  | GO:0050892 | Intestinal absorption | 1.00E-04 | 1.23E-02 |
|  | GO:0030299 | Intestinal cholesterol absorption | 3.00E-04 | 2.69E-02 |
|  | GO:0006811 | Ion transport | 0.00E+00 | 0.00E+00 |
|  | GO:0070189 | Kynurenine metabolic process | 1.00E-04 | 1.04E-02 |
|  | GO:0015807 | L-amino acid transport | 6.00E-04 | 4.18E-02 |
|  | GO:0016042 | Lipid catabolic process | 0.00E+00 | 0.00E+00 |
|  | GO:0044241 | Lipid digestion | 0.00E+00 | 5.80E-03 |
|  | GO:0006629 | Lipid metabolic process | 0.00E+00 | 0.00E+00 |
|  | GO:0034439 | Lipoprotein lipid oxidation | 7.00E-04 | 4.64E-02 |
|  | GO:0042160 | Lipoprotein modification | 7.00E-04 | 4.64E-02 |
|  | GO:0042161 | Lipoprotein oxidation | 7.00E-04 | 4.64E-02 |
|  | GO:0046466 | Membrane lipid catabolic process | 0.00E+00 | 0.00E+00 |
|  | GO:0007017 | Microtubule-based process | 4.00E-04 | 3.07E-02 |
|  | GO:0072330 | Monocarboxylic acid biosynthetic process | 0.00E+00 | 3.20E-03 |
|  | GO:0032787 | Monocarboxylic acid metabolic process | 0.00E+00 | 3.00E-04 |
|  | GO:0015672 | Monovalent inorganic cation transport | 0.00E+00 | 5.00E-04 |
|  | GO:0044243 | Multicellular organismal catabolic process | 0.00E+00 | 0.00E+00 |
|  | GO:0007275 | Multicellular organismal development | 2.00E-04 | 2.15E-02 |
|  | GO:0044240 | Multicellular organismal lipid catabolic process | 2.00E-04 | 1.58E-02 |
|  | GO:0044259 | Multicellular organismal macromolecule metabolic process | 0.00E+00 | 1.00E-04 |
|  | GO:0044236 | Multicellular organismal metabolic process | 0.00E+00 | 0.00E+00 |
|  | GO:0032501 | Multicellular organismal process | 0.00E+00 | 0.00E+00 |
|  | GO:0030239 | Myofibril assembly | 2.00E-04 | 2.00E-02 |
|  | GO:1900016 | Negative regulation of cytokine production involved in inflammatory response | 7.00E-04 | 4.64E-02 |
|  | GO:0002686 | Negative regulation of leukocyte migration | 7.00E-04 | 4.68E-02 |
|  | GO:0060588 | Negative regulation of lipoprotein lipid oxidation | 7.00E-04 | 4.64E-02 |
|  | GO:0050748 | Negative regulation of lipoprotein metabolic process | 7.00E-04 | 4.64E-02 |
|  | GO:0034443 | Negative regulation of lipoprotein oxidation | 7.00E-04 | 4.64E-02 |
|  | GO:2000402 | Negative regulation of lymphocyte migration | 7.00E-04 | 4.64E-02 |
|  | GO:0061060 | Negative regulation of peptidoglycan recognition protein signaling pathway | 0.00E+00 | 4.00E-04 |
|  | GO:2000405 | Negative regulation of T cell migration | 7.00E-04 | 4.64E-02 |
|  | GO:0050877 | Neurological system process | 0.00E+00 | 5.30E-03 |
|  | GO:0007218 | Neuropeptide signaling pathway | 0.00E+00 | 0.00E+00 |
|  | GO:0042135 | Neurotransmitter catabolic process | 6.00E-04 | 4.62E-02 |
|  | GO:0006836 | Neurotransmitter transport | 0.00E+00 | 0.00E+00 |
|  | GO:0009112 | Nucleobase metabolic process | 4.00E-04 | 3.41E-02 |
|  | GO:0016054 | Organic acid catabolic process | 0.00E+00 | 0.00E+00 |
|  | GO:0015849 | Organic acid transport | 2.00E-04 | 1.82E-02 |
|  | GO:0015711 | Organic anion transport | 2.00E-04 | 1.76E-02 |
|  | GO:1901616 | Organic hydroxy compound catabolic process | 1.00E-04 | 9.60E-03 |
|  | GO:1901615 | Organic hydroxy compound metabolic process | 0.00E+00 | 1.70E-03 |
|  | GO:1901565 | Organonitrogen compound catabolic process | 0.00E+00 | 0.00E+00 |
|  | GO:1901564 | Organonitrogen compound metabolic process | 1.00E-04 | 1.04E-02 |
|  | GO:0009253 | Peptidoglycan catabolic process | 0.00E+00 | 0.00E+00 |
|  | GO:0000270 | Peptidoglycan metabolic process | 0.00E+00 | 1.00E-04 |
|  | GO:0061057 | Peptidoglycan recognition protein signaling pathway | 0.00E+00 | 1.00E-04 |
|  | GO:0042811 | Pheromone biosynthetic process | 0.00E+00 | 6.00E-03 |
|  | GO:0042810 | Pheromone metabolic process | 0.00E+00 | 6.00E-03 |
|  | GO:0009395 | Phospholipid catabolic process | 1.00E-04 | 1.23E-02 |
|  | GO:0002807 | Positive regulation of antimicrobial peptide biosynthetic process | 5.00E-04 | 3.84E-02 |
|  | GO:0034250 | Positive regulation of cellular amide metabolic process | 5.00E-04 | 3.84E-02 |
|  | GO:0045297 | Post-mating behavior | 0.00E+00 | 0.00E+00 |
|  | GO:1901160 | Primary amino compound metabolic process | 6.00E-04 | 4.18E-02 |
|  | GO:0006693 | Prostaglandin metabolic Process | 0.00E+00 | 1.00E-04 |
|  | GO:0006692 | Prostanoid metabolic Process | 0.00E+00 | 1.00E-04 |
|  | GO:0015992 | Proton transport | 8.00E-04 | 4.82E-02 |
|  | GO:0006144 | Purine nucleobase metabolic process | 0.00E+00 | 2.60E-03 |
|  | GO:0002759 | Regulation of antimicrobial humoral response | 6.00E-04 | 4.23E-02 |
|  | GO:0002805 | Regulation of antimicrobial peptide biosynthetic process | 4.00E-04 | 3.01E-02 |
|  | GO:0002784 | Regulation of antimicrobial peptide production | 4.00E-04 | 3.01E-02 |
|  | GO:0034248 | Regulation of cellular amide metabolic process | 4.00E-04 | 3.61E-02 |
|  | GO:1900015 | Regulation of cytokine production involved in inflammatory response | 7.00E-04 | 4.64E-02 |
|  | GO:1900424 | Regulation of defense response to bacterium | 0.00E+00 | 1.00E-04 |
|  | GO:0010817 | Regulation of hormone levels | 8.00E-04 | 4.92E-02 |
|  | GO:0060587 | Regulation of lipoprotein lipid oxidation | 7.00E-04 | 4.64E-02 |
|  | GO:0034442 | Regulation of lipoprotein oxidation | 7.00E-04 | 4.64E-02 |
|  | GO:0001505 | Regulation of neurotransmitter levels | 0.00E+00 | 4.00E-03 |
|  | GO:0061058 | Regulation of peptidoglycan recognition protein signaling pathway | 0.00E+00 | 4.00E-04 |
|  | GO:0002700 | Regulation of production of molecular mediator of immune response | 4.00E-04 | 3.19E-02 |
|  | GO:0002831 | Regulation of response to biotic stimulus | 1.00E-04 | 1.41E-02 |
|  | GO:2000404 | Regulation of T cell migration | 7.00E-04 | 4.68E-02 |
|  | GO:0043555 | Regulation of translation in response to stress | 1.00E-04 | 6.70E-03 |
|  | GO:0010998 | Regulation of translational initiation by eIF2 alpha phosphorylation | 0.00E+00 | 3.00E-04 |
|  | GO:0043558 | Regulation of translational initiation in response to stress | 0.00E+00 | 4.40E-03 |
|  | GO:0019098 | Reproductive behavior | 0.00E+00 | 3.60E-03 |
|  | GO:0009617 | Response to bacterium | 1.00E-04 | 1.20E-02 |
|  | GO:0042493 | Response to drug | 1.00E-04 | 1.23E-02 |
|  | GO:0001895 | Retina homeostasis | 5.00E-04 | 3.74E-02 |
|  | GO:0007600 | Sensory perception | 0.00E+00 | 2.20E-03 |
|  | GO:0042428 | Serotonin metabolic process | 6.00E-04 | 4.18E-02 |
|  | GO:0044707 | Single-multicellular organism process | 0.00E+00 | 0.00E+00 |
|  | GO:0044708 | Single-organism behavior | 0.00E+00 | 2.60E-03 |
|  | GO:0044712 | Single-organism catabolic process | 0.00E+00 | 0.00E+00 |
|  | GO:0044767 | Single-organism developmental process | 0.00E+00 | 1.70E-03 |
|  | GO:0044699 | Single-organism process | 0.00E+00 | 0.00E+00 |
|  | GO:0030241 | Skeletal muscle myosin thick filament assembly | 4.00E-04 | 3.01E-02 |
|  | GO:0014866 | Skeletal myofibril assembly | 5.00E-04 | 4.02E-02 |
|  | GO:0044282 | Small molecule catabolic process | 0.00E+00 | 0.00E+00 |
|  | GO:0044281 | Small molecule metabolic process | 0.00E+00 | 5.00E-04 |
|  | GO:0006814 | Sodium ion transport | 0.00E+00 | 4.00E-04 |
|  | GO:0030149 | Sphingolipid catabolic process | 5.00E-04 | 4.02E-02 |
|  | GO:0051146 | Striated muscle cell differentiation | 7.00E-04 | 4.64E-02 |
|  | GO:0071688 | Striated muscle myosin thick filament assembly | 6.00E-04 | 4.43E-02 |
|  | GO:0007268 | Synaptic transmission | 0.00E+00 | 5.20E-03 |
|  | GO:0003008 | System process | 0.00E+00 | 1.00E-04 |
|  | GO:0072678 | T cell migration | 7.00E-04 | 4.68E-02 |
|  | GO:0042412 | Taurine biosynthetic process | 2.00E-04 | 1.58E-02 |
|  | GO:0019530 | Taurine metabolic process | 3.00E-04 | 2.69E-02 |
|  | GO:0070493 | Thrombin receptor signaling pathway | 3.00E-04 | 2.57E-02 |
|  | GO:0006569 | Tryptophan catabolic process | 1.00E-04 | 1.04E-02 |
|  | GO:0019441 | Tryptophan catabolic process to kynurenine | 7.00E-04 | 4.64E-02 |
|  | GO:0006568 | Tryptophan metabolic process | 1.00E-04 | 1.23E-02 |
|  | GO:0033559 | Unsaturated fatty acid metabolic process | 0.00E+00 | 4.10E-03 |
|  | GO:0042761 | Very long-chain fatty acid biosynthetic process | 1.00E-04 | 6.30E-03 |
|  | GO:0010025 | Wax biosynthetic process | 0.00E+00 | 0.00E+00 |
|  | GO:0010166 | Wax metabolic process | 0.00E+00 | 0.00E+00 |
| Cellular Component | GO:0031225 | Anchored component of membrane | 3.00E-04 | 2.77E-02 |
|  | GO:0005929 | Cilium | 0.00E+00 | 7.00E-04 |
|  | GO:0044430 | Cytoskeletal part | 4.00E-04 | 2.98E-02 |
|  | GO:0005576 | Extracellular region | 0.00E+00 | 0.00E+00 |
|  | GO:0044421 | Extracellular region part | 0.00E+00 | 0.00E+00 |
|  | GO:0005615 | Extracellular space | 0.00E+00 | 0.00E+00 |
|  | GO:0016021 | Integral component of membrane | 0.00E+00 | 0.00E+00 |
|  | GO:0031224 | Intrinsic component of membrane | 0.00E+00 | 0.00E+00 |
|  | GO:0005811 | Lipid particle | 3.00E-04 | 2.66E-02 |
|  | GO:0005764 | Lysosome | 4.00E-04 | 2.98E-02 |
|  | GO:0044425 | Membrane part | 0.00E+00 | 0.00E+00 |
|  | GO:0005859 | Muscle myosin complex | 4.00E-04 | 2.77E-02 |
|  | GO:0016459 | Myosin complex | 0.00E+00 | 6.80E-03 |
|  | GO:0032982 | Myosin filament | 0.00E+00 | 1.00E-04 |
|  | GO:0044459 | Plasma membrane part | 0.00E+00 | 3.00E-04 |
|  | GO:0030141 | Secretory granule | 3.00E-04 | 2.77E-02 |
|  | GO:0097223 | Sperm part | 5.00E-04 | 3.30E-02 |
|  | GO:0005863 | Striated muscle myosin thick filament | 1.00E-04 | 8.20E-03 |
|  | GO:0045202 | Synapse | 3.00E-04 | 2.77E-02 |
|  | GO:0044456 | Synapse part | 1.00E-04 | 8.20E-03 |
|  | GO:0008021 | Synaptic vesicle | 4.00E-04 | 2.77E-02 |
|  | GO:0030672 | Synaptic vesicle membrane | 0.00E+00 | 5.00E-04 |
| Molecular Function | GO:0004313 | [Acyl-carrier-protein] S-acetyltransferase activity | 0.00E+00 | 2.50E-03 |
|  | GO:0016404 | 15-Hydroxyprostaglandin dehydrogenase (NAD+) activity | 1.70E-03 | 4.86E-02 |
|  | GO:0019171 | 3-Hydroxyacyl-[acyl-carrier-protein] dehydratase activity | 0.00E+00 | 2.50E-03 |
|  | GO:0047451 | 3-Hydroxyoctanoyl-[acyl-carrier-protein] dehydratase activity | 0.00E+00 | 2.50E-03 |
|  | GO:0004317 | 3-Hydroxypalmitoyl-[acyl-carrier-protein] dehydratase activity | 0.00E+00 | 2.50E-03 |
|  | GO:0004316 | 3-Oxoacyl-[acyl-carrier-protein] reductase (NADPH) activity | 2.00E-04 | 7.00E-03 |
|  | GO:0004315 | 3-Oxoacyl-[acyl-carrier-protein] synthase activity | 1.70E-03 | 4.91E-02 |
|  | GO:0003990 | Acetylcholinesterase activity | 9.00E-04 | 2.88E-02 |
|  | GO:0022804 | Active transmembrane transporter activity | 0.00E+00 | 4.00E-04 |
|  | GO:0016297 | Acyl-[acyl-carrier-protein] hydrolase activity | 0.00E+00 | 2.50E-03 |
|  | GO:0017105 | Acyl-CoA delta11-desaturase activity | 4.00E-04 | 1.35E-02 |
|  | GO:0047372 | Acylglycerol lipase activity | 0.00E+00 | 0.00E+00 |
|  | GO:0015151 | Alpha-glucoside transmembrane transporter activity | 0.00E+00 | 0.00E+00 |
|  | GO:0004040 | Amidase activity | 0.00E+00 | 3.00E-04 |
|  | GO:0004177 | Aminopeptidase activity | 6.00E-04 | 1.97E-02 |
|  | GO:0070330 | Aromatase activity | 0.00E+00 | 0.00E+00 |
|  | GO:0005509 | Calcium ion binding | 0.00E+00 | 0.00E+00 |
|  | GO:0015144 | Carbohydrate transmembrane transporter activity | 0.00E+00 | 0.00E+00 |
|  | GO:1901476 | Carbohydrate transporter activity | 0.00E+00 | 0.00E+00 |
|  | GO:0052689 | Carboxylic ester hydrolase activity | 0.00E+00 | 0.00E+00 |
|  | GO:0004180 | Carboxypeptidase activity | 0.00E+00 | 1.00E-04 |
|  | GO:0005261 | Cation channel activity | 1.00E-04 | 3.90E-03 |
|  | GO:0008324 | Cation transmembrane transporter activity | 1.00E-04 | 6.30E-03 |
|  | GO:0015267 | Channel activity | 1.00E-04 | 3.90E-03 |
|  | GO:0008061 | Chitin binding | 0.00E+00 | 0.00E+00 |
|  | GO:0008812 | Choline dehydrogenase activity | 0.00E+00 | 5.00E-04 |
|  | GO:0004104 | Cholinesterase activity | 1.60E-03 | 4.65E-02 |
|  | GO:0015154 | Disaccharide transmembrane transporter activity | 0.00E+00 | 0.00E+00 |
|  | GO:0009055 | Electron carrier activity | 4.00E-04 | 1.59E-02 |
|  | GO:0004866 | Endopeptidase inhibitor activity | 1.10E-03 | 3.40E-02 |
|  | GO:0004319 | Enoyl-[acyl-carrier-protein] reductase (NADPH, B-specific) activity | 0.00E+00 | 2.50E-03 |
|  | GO:0008238 | Exopeptidase activity | 0.00E+00 | 1.00E-04 |
|  | GO:0004312 | Fatty acid synthase activity | 1.50E-03 | 4.40E-02 |
|  | GO:0080019 | Fatty-acyl-CoA reductase (alcohol-forming) activity | 0.00E+00 | 0.00E+00 |
|  | GO:0047714 | Galactolipase activity | 4.00E-04 | 1.58E-02 |
|  | GO:0022836 | Gated channel activity | 3.00E-04 | 1.19E-02 |
|  | GO:0004344 | Glucose dehydrogenase activity | 0.00E+00 | 0.00E+00 |
|  | GO:0042947 | Glucoside transmembrane transporter activity | 0.00E+00 | 0.00E+00 |
|  | GO:0004930 | G-protein coupled receptor activity | 0.00E+00 | 1.20E-03 |
|  | GO:0016787 | Hydrolase activity | 0.00E+00 | 3.00E-04 |
|  | GO:0016810 | Hydrolase activity, acting on carbon-nitrogen (but not peptide) bonds | 0.00E+00 | 3.00E-04 |
|  | GO:0016814 | Hydrolase activity, acting on carbon-nitrogen (but not peptide) bonds, in cyclic amidines | 1.70E-03 | 4.98E-02 |
|  | GO:0016811 | Hydrolase activity, acting on carbon-nitrogen (but not peptide) bonds, in linear amides | 2.00E-04 | 9.20E-03 |
|  | GO:0016798 | Hydrolase activity, acting on glycosyl bonds | 0.00E+00 | 2.10E-03 |
|  | GO:0004553 | Hydrolase activity, hydrolyzing O-glycosyl compounds | 1.00E-04 | 3.10E-03 |
|  | GO:0004953 | Icosanoid receptor activity | 4.00E-04 | 1.59E-02 |
|  | GO:0022890 | Inorganic cation transmembrane transporter activity | 2.00E-04 | 9.20E-03 |
|  | GO:0005520 | Insulin-like growth factor binding | 1.00E-03 | 3.18E-02 |
|  | GO:0005216 | Ion channel activity | 1.00E-04 | 3.00E-03 |
|  | GO:0015075 | Ion transmembrane transporter activity | 0.00E+00 | 1.00E-04 |
|  | GO:0005506 | Iron ion binding | 0.00E+00 | 6.00E-04 |
|  | GO:0016298 | Lipase activity | 0.00E+00 | 0.00E+00 |
|  | GO:0050062 | Long-chain-fatty-acyl-CoA reductase activity | 0.00E+00 | 0.00E+00 |
|  | GO:0004794 | L-threonine ammonia-lyase activity | 2.00E-04 | 9.20E-03 |
|  | GO:0045289 | Luciferin monooxygenase activity | 1.00E-04 | 3.60E-03 |
|  | GO:0046873 | Metal ion transmembrane transporter activity | 4.00E-04 | 1.37E-02 |
|  | GO:0004181 | Metallocarboxypeptidase activity | 0.00E+00 | 0.00E+00 |
|  | GO:0008235 | Metalloexopeptidase activity | 0.00E+00 | 2.00E-04 |
|  | GO:0008237 | Metallopeptidase activity | 3.00E-04 | 1.30E-02 |
|  | GO:0080030 | Methyl indole-3-acetate esterase activity | 0.00E+00 | 0.00E+00 |
|  | GO:0080032 | Methyl jasmonate esterase activity | 0.00E+00 | 0.00E+00 |
|  | GO:0080031 | Methyl salicylate esterase activity | 0.00E+00 | 0.00E+00 |
|  | GO:0004497 | Monooxygenase activity | 0.00E+00 | 0.00E+00 |
|  | GO:0015077 | Monovalent inorganic cation transmembrane transporter activity | 5.00E-04 | 1.66E-02 |
|  | GO:0016295 | Myristoyl-[acyl-carrier-protein] hydrolase activity | 0.00E+00 | 2.50E-03 |
|  | GO:0052899 | N(1), N(12)-Diacetylspermine:oxygen oxidoreductase (3-acetamidopropanal-forming) activity | 2.00E-04 | 6.70E-03 |
|  | GO:0052904 | N1-Acetylspermidine:oxygen oxidoreductase (3-acetamidopropanal-forming) activity | 5.00E-04 | 1.61E-02 |
|  | GO:0052903 | N1-Acetylspermine:oxygen oxidoreductase (3-acetamidopropanal-forming) activity | 5.00E-04 | 1.61E-02 |
|  | GO:0008745 | N-Acetylmuramoyl-L-alanine amidase activity | 0.00E+00 | 0.00E+00 |
|  | GO:0070403 | NAD+ binding | 1.60E-03 | 4.65E-02 |
|  | GO:0005326 | Neurotransmitter transporter activity | 1.20E-03 | 3.62E-02 |
|  | GO:0005549 | Odorant binding | 8.00E-04 | 2.74E-02 |
|  | GO:0004320 | Oleoyl-[acyl-carrier-protein] hydrolase activity | 0.00E+00 | 2.50E-03 |
|  | GO:0015157 | Oligosaccharide transmembrane transporter activity | 0.00E+00 | 0.00E+00 |
|  | GO:0016491 | Oxidoreductase activity | 0.00E+00 | 0.00E+00 |
|  | GO:0016614 | Oxidoreductase activity, acting on CH-OH group of donors | 0.00E+00 | 0.00E+00 |
|  | GO:0016705 | Oxidoreductase activity, acting on paired donors, with incorporation or reduction of molecular oxygen | 0.00E+00 | 1.00E-04 |
|  | GO:0016712 | Oxidoreductase activity, acting on paired donors, with incorporation or reduction of molecular oxygen, reduced flavin or flavoprotein as one donor, and incorporation of one atom of oxygen | 0.00E+00 | 0.00E+00 |
|  | GO:0016620 | Oxidoreductase activity, acting on the aldehyde or oxo group of donors, NAD or NADP as acceptor | 1.20E-03 | 3.62E-02 |
|  | GO:0016628 | Oxidoreductase activity, acting on the CH-CH group of donors, NAD or NADP as acceptor | 1.00E-04 | 5.50E-03 |
|  | GO:0016616 | Oxidoreductase activity, acting on the CH-OH group of donors, NAD or NADP as acceptor | 0.00E+00 | 3.00E-04 |
|  | GO:0016296 | Palmitoyl-[acyl-carrier-protein] hydrolase activity | 0.00E+00 | 2.50E-03 |
|  | GO:0022803 | Passive transmembrane transporter activity | 1.00E-04 | 3.90E-03 |
|  | GO:0070011 | Peptidase activity, acting on L-amino acid Peptides | 1.20E-03 | 3.77E-02 |
|  | GO:0030414 | Peptidase inhibitor activity | 3.00E-04 | 1.19E-02 |
|  | GO:0061134 | Peptidase regulator activity | 1.30E-03 | 3.80E-02 |
|  | GO:0042834 | Peptidoglycan binding | 0.00E+00 | 0.00E+00 |
|  | GO:0015114 | Phosphate ion transmembrane transporter activity | 1.00E-04 | 3.60E-03 |
|  | GO:0004620 | Phospholipase activity | 6.00E-04 | 1.92E-02 |
|  | GO:0047077 | Photinus-luciferin 4-monooxygenase (ATP-hydrolyzing) activity | 1.00E-04 | 3.60E-03 |
|  | GO:0005267 | Potassium channel activity | 3.00E-04 | 1.19E-02 |
|  | GO:0004957 | Prostaglandin E receptor activity | 2.00E-04 | 8.70E-03 |
|  | GO:0004955 | Prostaglandin receptor activity | 4.00E-04 | 1.59E-02 |
|  | GO:0004954 | Prostanoid receptor activity | 4.00E-04 | 1.59E-02 |
|  | GO:0004872 | Receptor activity | 0.00E+00 | 1.00E-04 |
|  | GO:0050253 | Retinyl-palmitate esterase activity | 0.00E+00 | 0.00E+00 |
|  | GO:0016418 | S-acetyltransferase activity | 7.00E-04 | 2.39E-02 |
|  | GO:0015291 | Secondary active transmembrane transporter activity | 0.00E+00 | 8.00E-04 |
|  | GO:0017171 | Serine hydrolase activity | 0.00E+00 | 0.00E+00 |
|  | GO:0004252 | Serine-type endopeptidase activity | 0.00E+00 | 0.00E+00 |
|  | GO:0004867 | Serine-type endopeptidase inhibitor activity | 8.00E-04 | 2.70E-02 |
|  | GO:0008236 | Serine-type peptidase activity | 0.00E+00 | 0.00E+00 |
|  | GO:0038023 | Signaling receptor activity | 1.00E-04 | 3.70E-03 |
|  | GO:0052902 | Spermidine:oxygen oxidoreductase (3-aminopropanal-forming) activity | 5.00E-04 | 1.61E-02 |
|  | GO:0004771 | Sterol esterase activity | 1.00E-03 | 3.18E-02 |
|  | GO:0042302 | Structural constituent of cuticle | 0.00E+00 | 0.00E+00 |
|  | GO:0008307 | Structural constituent of muscle | 2.00E-04 | 9.50E-03 |
|  | GO:0022838 | Substrate-specific channel activity | 1.00E-04 | 2.90E-03 |
|  | GO:0022891 | Substrate-specific transmembrane transporter activity | 0.00E+00 | 0.00E+00 |
|  | GO:0022892 | Substrate-specific transporter activity | 0.00E+00 | 0.00E+00 |
|  | GO:0051119 | Sugar transmembrane transporter activity | 0.00E+00 | 0.00E+00 |
|  | GO:0015293 | Symporter activity | 0.00E+00 | 0.00E+00 |
|  | GO:0019137 | Thioglucosidase activity | 0.00E+00 | 1.90E-03 |
|  | GO:0004888 | Transmembrane signaling receptor activity | 0.00E+00 | 1.00E-03 |
|  | GO:0022857 | Transmembrane transporter activity | 0.00E+00 | 0.00E+00 |
|  | GO:0005215 | Transporter activity | 0.00E+00 | 0.00E+00 |
|  | GO:0015574 | Trehalose transmembrane transporter activity | 0.00E+00 | 0.00E+00 |
|  | GO:0004806 | Triglyceride lipase activity | 0.00E+00 | 0.00E+00 |
|  | GO:0004835 | Tubulin-tyrosine ligase activity | 4.00E-04 | 1.35E-02 |
|  | GO:0022843 | Voltage-gated cation channel activity | 1.00E-03 | 3.08E-02 |
|  | GO:0005249 | Voltage-gated potassium channel activity | 1.70E-03 | 4.84E-02 |
