# Supplementary figures and images for: Differential gene expression patterns between the head and thorax of Gynaephora aureata are associated with high-altitude adaptation
Source: Front Genet. 2023 Apr 18;14:1137618. doi: 10.3389/fgene.2023.1137618 (PMC10151491; doi:10.3389/fgene.2023.1137618)

A

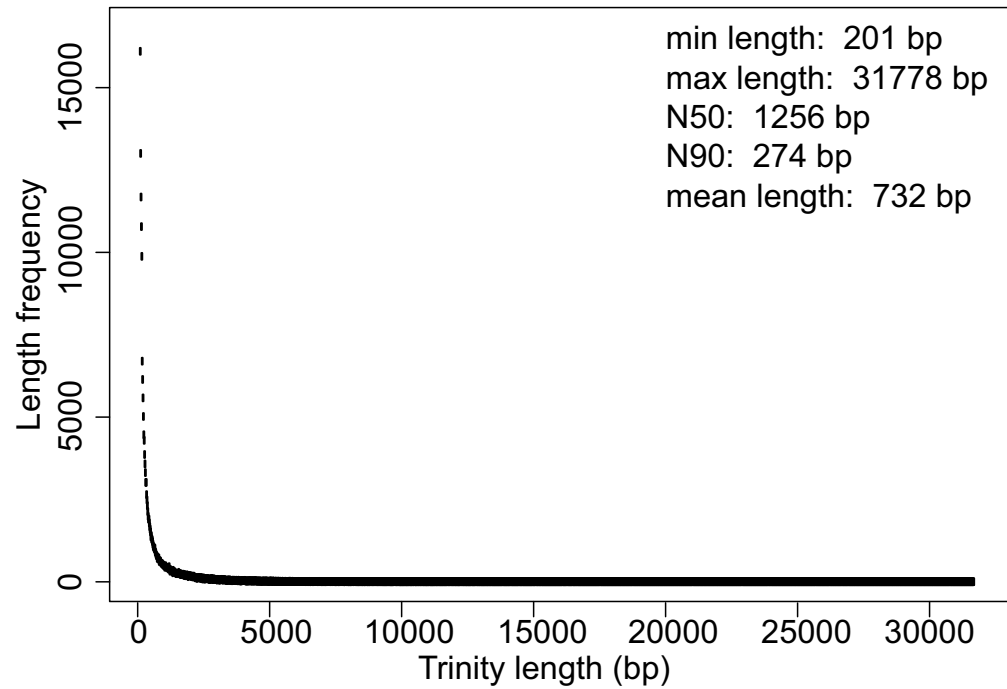

B

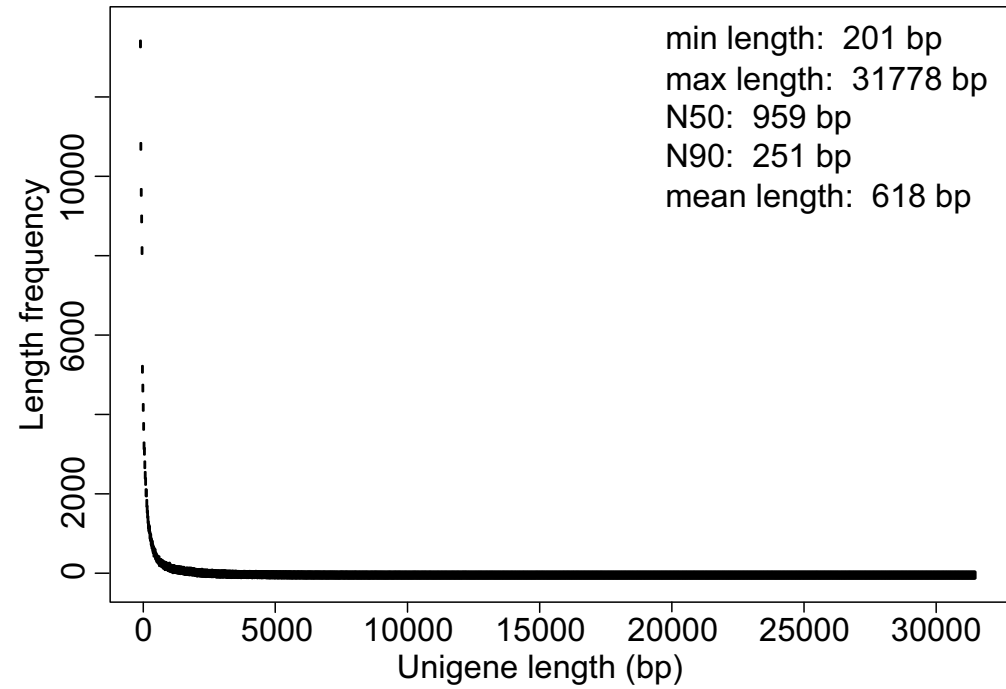

Supplement: Supplementary file 1 [file DataSheet1.zip › Figure S1.pdf]

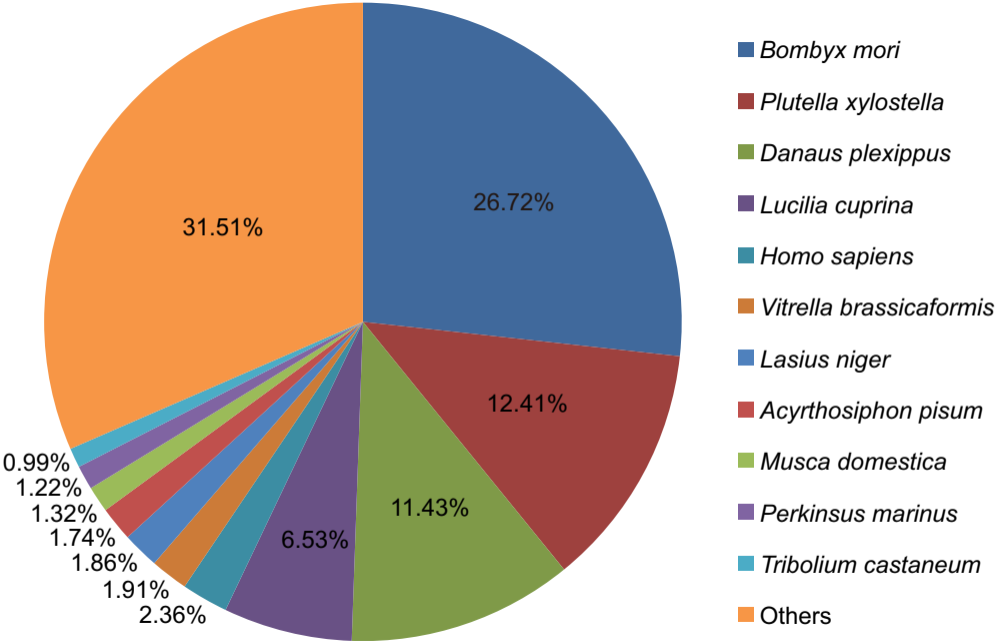

Supplement: Supplementary file 1 [file DataSheet1.zip › Figure S2.pdf]

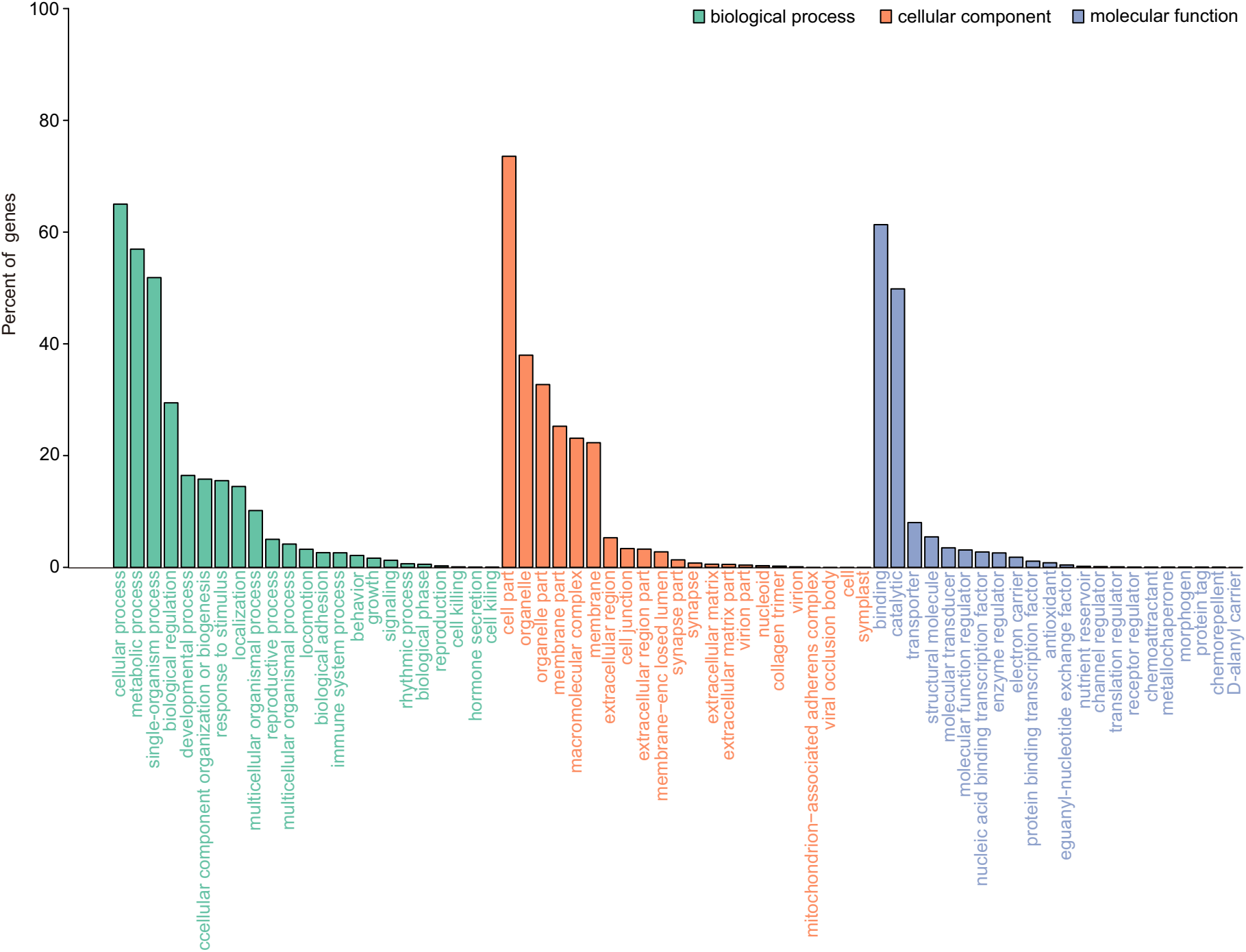

Supplement: Supplementary file 1 [file DataSheet1.zip › Figure S3.pdf]

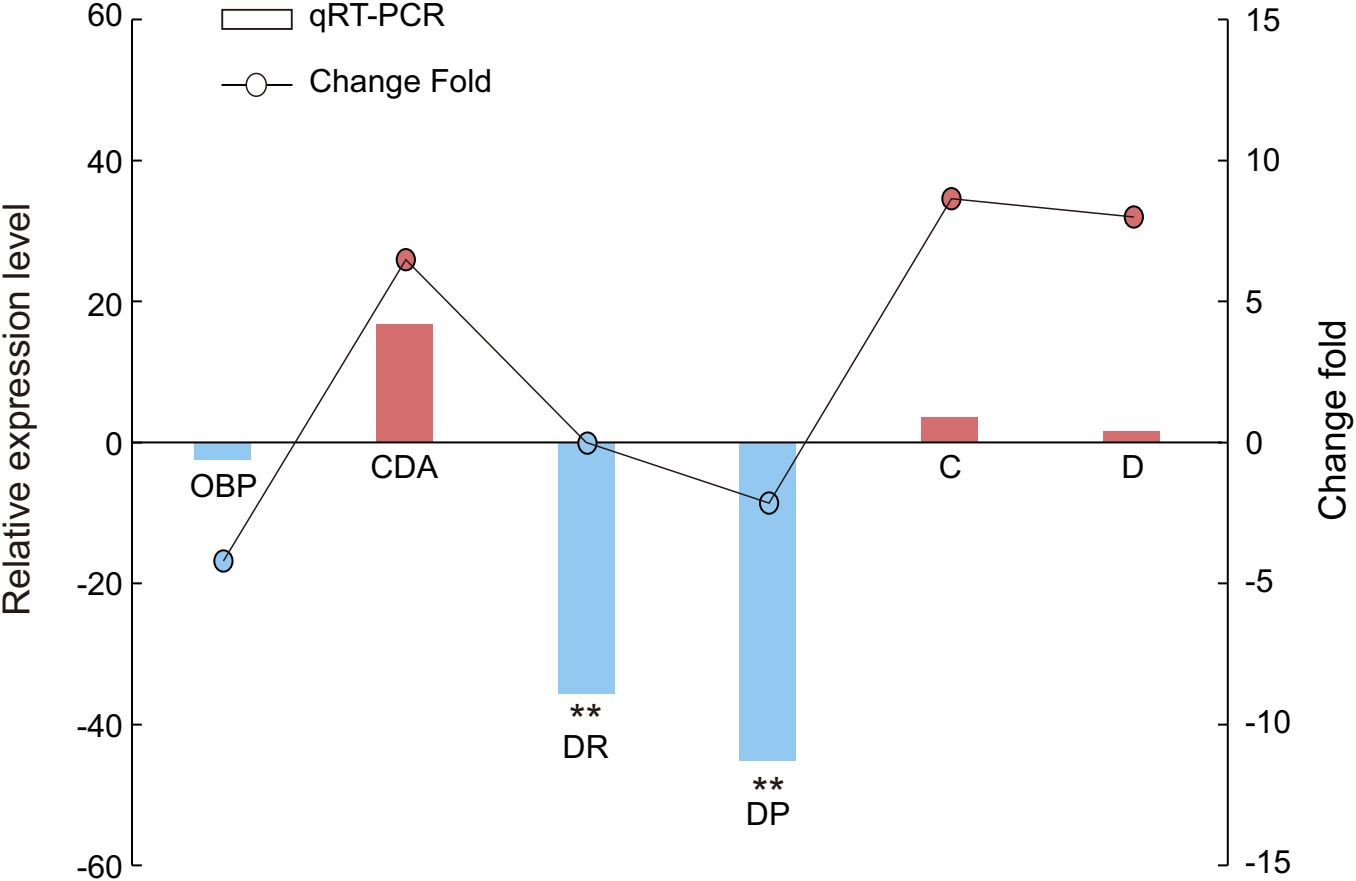

Supplement: Supplementary file 1 [file DataSheet1.zip › Figure S5.pdf]

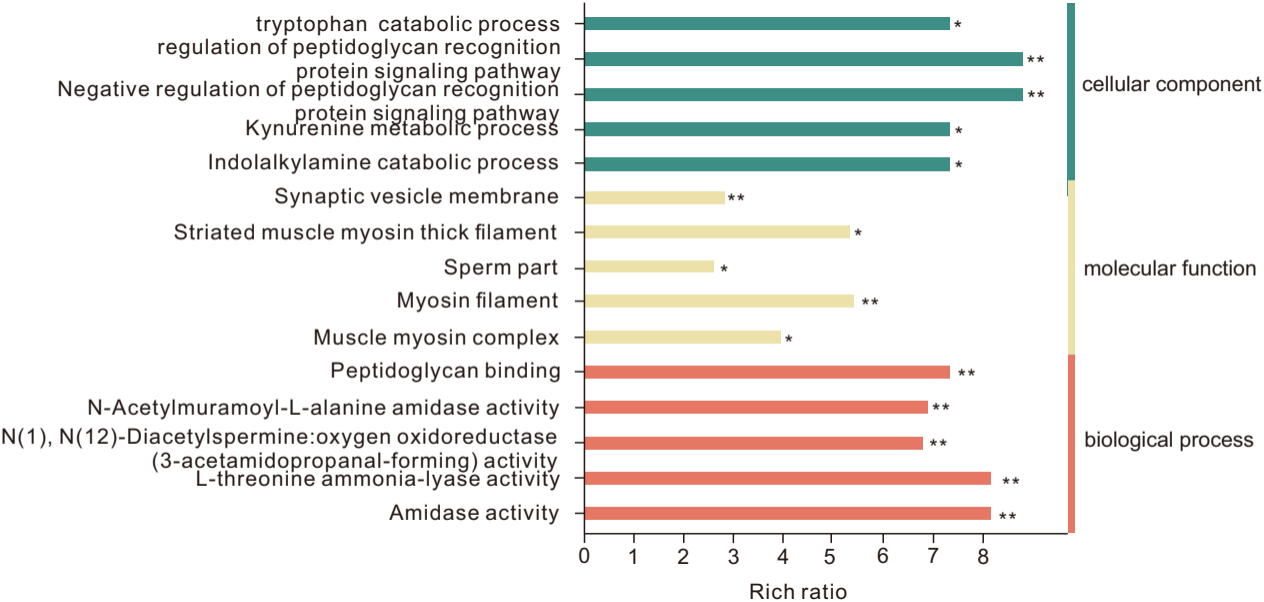

Supplement: Supplementary file 1 [file DataSheet1.zip › Figure S6.pdf]

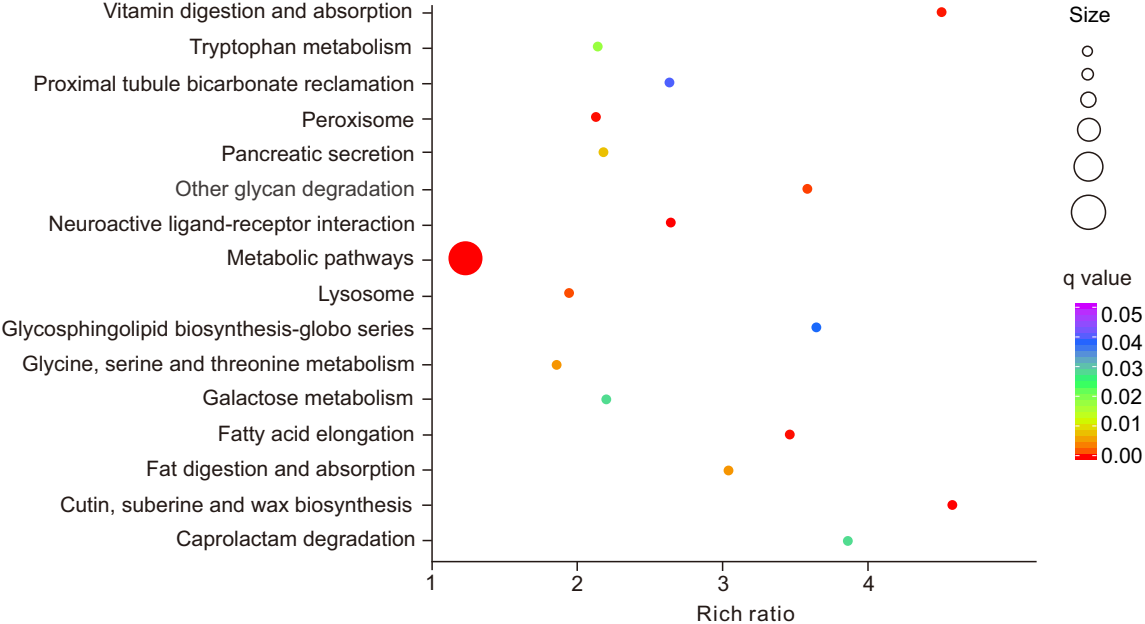

Supplement: Supplementary file 1 [file DataSheet1.zip › Figure S7.pdf]
